# Dietary Supplement Strategies During Conditioning Training in Athletes: A Network Meta‐Analysis of Peak and Mean Anaerobic Power, VO_2_max, and Endurance Performance

**DOI:** 10.1002/fsn3.71243

**Published:** 2025-11-29

**Authors:** Beiwang Deng, Haoran Li, Cheng Chen, Jiaxin He, Wenfeng Zhang, Hong Lin

**Affiliations:** ^1^ School of Athletic Training Guangzhou Sport University Guangzhou Guangdong China; ^2^ Key Laboratory of Human‐Computer Intelligent Interaction for Athletic Performance and HealthPromotion Guangzhou Sport University Guangzhou China

**Keywords:** ergogenic aids, sport nutrition, sport performance, sportsman, supplementation

## Abstract

Dietary supplements are often used during training to support energy provision, recovery, and adaptation, yet effects on peak and mean anaerobic power, VO_2_max, and endurance performance remain inconsistent. Unlike prior syntheses centred on neuromuscular outcomes (strength and hypertrophy), this study compared six common supplements—protein, creatine, β‐alanine, HMB, vitamin D, and nitrate—on energy‐system performance (peak anaerobic power, mean anaerobic power, VO_2_max, and endurance performance) using network meta‐analysis. PubMed, Web of Science, Cochrane Library, Embase, and SPORTDiscus were searched from inception to 15 March 2025 for RCTs examining dietary supplements and athletic performance. Risk of bias was assessed with the revised Cochrane tool. Random‐effects network meta‐analyses were conducted in R 4.3.1 and Stata 18. Thirty RCTs involving 693 athletes undergoing training programmes combined with supplements or placebo met the inclusion criteria. For peak anaerobic power, protein (SMD 0.85; 95% CI 0.27–1.44; SUCRA 82.9%; moderate certainty), creatine (0.62; 0.20–1.03; 62.5%; moderate), HMB (0.60; 0.28–0.94; 60.7%; moderate), and β‐alanine (0.58; 0.09–1.07; 57.4%; very low) were superior to placebo. In mean power, β‐alanine (0.75; 0.20–1.31; 74.1%; very low), protein (0.74; 0.27–1.20; 73.9%; very low), and creatine (0.74; 0.08–1.39; 71.0%; very low) showed consistent benefits, while HMB provided a moderate improvement (0.45; 0.05–0.86; 45.8%; very low). Endurance performance improved only with protein (0.99; 0.16–1.83; 94.3%; very low). No supplement affected VO_2_max. Protein most effectively enhances peak power and endurance performance; β‐alanine and creatine excel in mean power; creatine and HMB also aid peak power; none improve VO_2_max.

**Trial Registration:** PROSPERO identifier: CRD420251106522

## Graphical abstract

In this network meta‐analysis of 30 RCTs (*n* = 693), we compared six supplements during training on energy‐system outcomes, with certainty ranging from very low to moderate. Protein ranked highest for peak power and showed the most consistent benefit for endurance performance; β‐alanine, protein, and creatine tended to improve mean power, while creatine and HMB were also favorable for peak power. Effects on VO_2_max were imprecise and generally null; vitamin D and nitrate showed limited, non‐robust benefits.

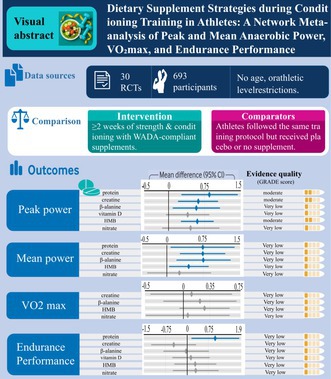

**Abbreviations**
BA: β‐alanine
CI: confidence interval
CINeMA: Confidence in Network Meta‐Analysis
CR: creatine
HMB: β‐hydroxy‐β‐methylbutyrate
IOC: International Olympic Committee
MD: mean difference
nitrate: NO_3_
NMA: network meta‐analysis
PCr: phosphocreatine
PL: placebo
PR: protein
RCTs: randomized controlled trials
S&C: strength and conditioning
SD: standard deviation
SE: standard error
SMD: standardized mean difference
SUCRA: surface under the cumulative ranking curve
VD: Vitamin D3
VO_2_max: maximal oxygen uptake

## 1 Introduction

Enhancing athletic performance has long been a central objective of sport science, and the synergistic interaction between physical training programmes and nutrition strategies has received increasing attention in recent years (Guest et al. 2021; Perez‐Schindler et al. 2015). Dietary supplementation, in particular, is now ubiquitous in elite sport—surveys indicate that between 40% and nearly 100% of athletes consume at least one supplement, with prevalence rising alongside competitive level (Daher et al. 2022). Despite the vast number of products on the market, only a few possess robust, evidence‐based support for ergogenic benefits (Zart and Fröhlich 2025). On this basis, international authorities such as the Australian Institute of Sport classify caffeine, creatine, β‐alanine, nitrate sources (e.g., beetroot juice), and sodium bicarbonate as Group A supplements, meaning there is strong evidence for their performance‐enhancing effects (Zart and Fröhlich 2025). Recent meta‐analyses further confirm that, when used correctly, these validated supplements can exert pronounced positive effects on athletic outcomes (Castell et al. 2015).

Athletic success depends on the balanced development of both aerobic and anaerobic capacities, which are critical across a wide range of sporting disciplines (Bellar et al. 2015). Aerobic capacity—typically quantified by maximal oxygen uptake (VO_2_max) or related endurance tests—is a key determinant of performance in endurance events (Bosquet et al. 2002). Conversely, anaerobic explosive ability, assessed by peak and mean power outputs during short‐duration sprint tasks, is decisive in strength‐ and speed‐oriented events (Cronin and Sleivert 2005). For coaches and athletes, simultaneously improving aerobic endurance and anaerobic power is a core training objective, because most competitive sports demand both durability and explosiveness (Sandford et al. 2021). Accordingly, VO_2_max and sprint power are routinely employed as key outcome measures for assessing the effectiveness of training or nutritional interventions in exercise‐training research (Viribay et al. 2020).

With respect to nutritional strategies, substantial evidence demonstrates that certain supplements significantly augment aerobic endurance performance (Aguiló et al. 2007; Rothschild and Bishop 2020). A series of randomized trials and meta‐analyses show caffeine to be one of the most effective supplements for enhancing endurance: appropriate doses markedly improve time‐trial performance and prolong time to exhaustion, yielding an overall effect size of approximately 0.4 (Zart and Fröhlich 2025). By increasing intramuscular carnosine, β‐alanine supplementation also benefits high‐intensity endurance exercise, albeit with a smaller but statistically significant effect (Zart and Fröhlich 2025). Moreover, dietary nitrate—popularized by nitrate‐rich beetroot juice—has been shown to enhance exercise economy and endurance performance, although its effect size is modest (Zart and Fröhlich 2025). Notably, foundational nutrients may likewise influence training adaptations; for instance, additional protein intake during endurance training has been associated with greater VO_2_max gains compared with non‐supplemented controls (Knuiman et al. 2019). Nutritional strategies targeting anaerobic performance have been equally well studied. Creatine is the classic example: supplementation enlarges skeletal‐muscle phosphocreatine stores, facilitating rapid ATP resynthesis and thereby improving short‐duration, high‐intensity performance (Wyss and Kaddurah‐Daouk 2000). Numerous studies report that combining creatine intake with resistance or high‐intensity training produces larger increases in muscular strength and peak power, as well as better repeated‐sprint outcomes, than training alone (Burke et al. 2023). Supplements that bolster the body's buffering capacity—such as sodium bicarbonate—also enhance high‐intensity performance (Requena et al. 2005). A recent meta‐analysis showed that, relative to placebo, exogenous buffers confer small‐to‐moderate overall performance benefits (effect size ≈0.17), with the greatest improvements observed during intense efforts lasting 0.5–10 min (de Oliveira et al. 2022). Collectively, these findings suggest that judicious supplementation can further potentiate training adaptations in VO_2_max and anaerobic power.

Despite these promising trends, the relative efficacy of different supplements across key performance metrics remains incompletely understood; existing studies are fragmented and heterogeneous (Zart and Fröhlich 2025). Most interventions evaluate a single supplement, leaving a paucity of systematic evidence for direct comparisons among multiple nutritional strategies. This gap presents a practical challenge: coaches and athletes struggle to determine which supplement, when combined with training, is most effective for improving VO_2_max, endurance performance, or anaerobic power. Consequently, a comprehensive, quantitative synthesis of recent research is warranted. To this end, the present study will employ systematic review and network meta‐analysis to integrate recent evidence on supplements co‐administered with physical training, focusing on four critical outcomes—VO_2_max, endurance performance, peak power, and mean power. By simultaneously comparing multiple nutritional interventions across randomized controlled trials, this analysis aims to provide evidence‐based guidance for supplement use in competitive training contexts.

## 2 Methods

### 2.1 Study Registration

This study was conducted as a systematic review and network meta‐analysis in accordance with the PRISMA‐NMA guidelines (Hutton et al. 2015) and was prospectively registered in PROSPERO. Given the methodological complexity of comparing multiple interventions, we adopted the PRISMA‐NMA framework to ensure methodological rigor.

### 2.2 Search Strategy

The literature search encompassed PubMed, Web of Science, Embase, SPORTDiscus, and the Cochrane Central Register of Controlled Trials. We additionally screened clinical trial registries to identify unpublished data and examined the reference lists of all included studies for missing citations, without imposing restrictions on region, publication year, or language. These databases were chosen for their comprehensive coverage of sport‐nutrition and exercise‐science literature. Searches spanned from database inception to 15 March 2025. Detailed search strategies are provided in Appendix S1.

### 2.3 Inclusion and Exclusion Criteria

The eligibility criteria were specified a priori using the PICOS framework. Population: we included only systematically trained athletes of any sex or age; studies involving non‐athletes, injured or clinical cohorts, or animals were excluded. Interventions: trials had to combine at least 2 weeks of structured strength‐and‐conditioning (Chandler et al. 2019) or sport‐specific training with one or more dietary supplements that contained no ingredient on the World Anti‐Doping Agency Prohibited List; supplements could be provided singly or in combination. Comparators: control groups were required to follow the identical training protocol—matching frequency, intensity, and supervision—while receiving a placebo or no supplement. Outcomes: eligible studies reported at least one post‐intervention performance measure related to peak anaerobic power, mean anaerobic power, VO_2_max, or endurance performance (Table 1). Study design: only randomized controlled trials (parallel or crossover) were considered, irrespective of blinding; crossover trials had to incorporate an adequate wash‐out and, where carry‐over effects were possible, contribute data from the first period only. Studies were excluded if they involved carbohydrate or caffeine as the sole supplement (or as the dominant component of a multi‐ingredient formula), failed to specify supplement dose or timing, were not peer‐reviewed full‐text articles in English, lacked full‐text availability, or were non‐original publications (e.g., reviews, commentaries, case reports, or conference abstracts).

**TABLE 1:** Performance measures.

Outcome indicators	Exercise test
Peak anaerobic power	Wingate test (30‐s all‐out sprint) peak power
Mean anaerobic power	Wingate test (30‐s all‐out sprint) mean power
VO_2_max	VO_2_max via an incremental graded exercise test (e.g., Bruce or Balke treadmill protocol, etc.)
Endurance performance	Time‐trial (e.g., 5 km run, 20 km cycle) or time‐to‐exhaustion protocol

### 2.4 Study Selection and Data Extraction

The retrieved records were imported into Zotero 7. Titles and abstracts were screened to identify potentially eligible studies, after which the full texts were downloaded and assessed against the inclusion criteria to determine final eligibility. Before data extraction, we developed a standardized extraction form that captured the study title, first author, publication year, study design, authors' country, intervention characteristics, intervention duration, participant number, sex, age, sport discipline, exercise test, and outcome measures. Two investigators (B.W. and R.X.) independently conducted the screening and data extraction and cross‐checked their results. Discrepancies were resolved by consensus or, when outcome measures were unclear, through consultation with a third investigator (H.R.).

### 2.5 Masures of Treatment Effect

In this meta‐analysis, intervention effects were expressed as the change in mean difference (MD) and standard deviation (SD). When an original study did not report the SD directly, we derived it from the standard error (SE), 95% confidence interval (CI), *p*‐value or *t*‐statistic, following published guidance (Chandler et al. 2019). For trials that lacked the SD of the pre–post change, we calculated it with the equation:

$$\begin{array}{c} SD_{\text{change}}=\sqrt{SD_{\text{baseline}}^{2}+SD_{\text{post}}^{2}-2\mathrm{rS}D_{\text{baseline}}{\mathrm{SD}}_{\text{post}}} \end{array}$$

assuming a correlation coefficient (*r*) of 0.5 between baseline and follow‐up measurements (Higgins et al. 2019). This moderate value, commonly adopted in the literature, balances potential variability between repeated measures and thus supports the robustness and reliability of the pooled estimates.

### 2.6 Quality Assessment of Evidence

We evaluated each trial with the Cochrane Risk of Bias tool for randomized controlled trials (RoB 2.0), covering random sequence generation, allocation concealment, blinding, missing outcome data and selective outcome reporting (Higgins et al. 2016). A study was classified as “high overall risk of bias” if at least one domain was rated as “high risk” (score = 1). A study was classified as having “some concerns” if no domain was rated “high risk” but at least one domain was rated as “some concerns” (score = 2). A study was classified as “low overall risk of bias” if all domains were rated as “low risk” (score = 3). Two reviewers (B.W. and R.X.) conducted the assessments independently and resolved disagreements through discussion or, when necessary, consultation with a third reviewer (H.R.).

Certainty of evidence for every network estimate was graded using the Confidence in Network Meta‐Analysis (CINeMA) framework (Nikolakopoulou et al. 2020). This approach evaluates six domains: (i) within‐study bias (based on RoB 2.0 assessments of contributing studies), (ii) reporting bias (assessed via funnel plots and Egger's test where appropriate), (iii) indirectness (considering PICO alignment and transitivity), (iv) imprecision (determined by the 95% CI width), (v) heterogeneity (statistical and clinical), and (vi) incoherence (consistency between direct and indirect evidence, assessed via node‐splitting) (Nikolakopoulou et al. 2020; Papakonstantinou et al. 2020). Detailed criteria for evaluating each CINeMA domain, specific conditions leading to rating concerns, and downgrading rules are provided in Appendix S1: Detailed CINeMA Assessment Protocol. Two reviewers (B.W. and R.X.) independently assessed certainty, with discrepancies resolved by discussion or a third reviewer (H.R.).

### 2.7 Statistical Analysis

This network meta‐analysis was conducted within a frequentist framework using R 4.3.1 and Stata 18 through a graph‐theoretical approach. The netmeta package in R handled primary model fitting, node‐splitting, SUCRA computation, and visualization, whereas the network and mvmeta modules in Stata supported sensitivity analyses and funnel‐plot construction. Effect sizes were estimated via weighted least‐squares regression solved with the Moore–Penrose generalized inverse, and random‐effects models were specified throughout to account for between‐study heterogeneity (Welton et al. 2012). When outcomes were reported on identical scales, mean differences (MDs) were calculated; where different instruments or units were used (e.g., peak power or endurance performance), standardized mean differences (SMDs) with 95% confidence intervals (CIs) were pooled to ensure comparability. The network structure was visualized with network plots in which node size was proportional to sample size and edge thickness reflected the volume of direct evidence. Global consistency was evaluated by comparing consistency and inconsistency models (*p* < 0.05 indicating overall inconsistency), while local consistency was tested with node‐splitting (*p* < 0.05 for the difference between direct and indirect estimates denoting local inconsistency) (Dias et al. 2013). Intervention rankings were summarized as surface under the cumulative ranking curve (SUCRA) values and displayed as rank‐heat plots, with higher SUCRA indicating greater efficacy (Mbuagbaw et al. 2017; Veroniki et al. 2016). Publication bias was assessed by funnel‐plot symmetry and confirmed with Egger's test. The magnitude of the SMD was interpreted using Cohen's (2013) conventional criteria, where an SMD of approximately 0.2 was considered a small effect, 0.5 a moderate effect, and 0.8 a large effect (Cohen 2013).

## 3 Results

### 3.1 Search Results

**FIGURE 1:**
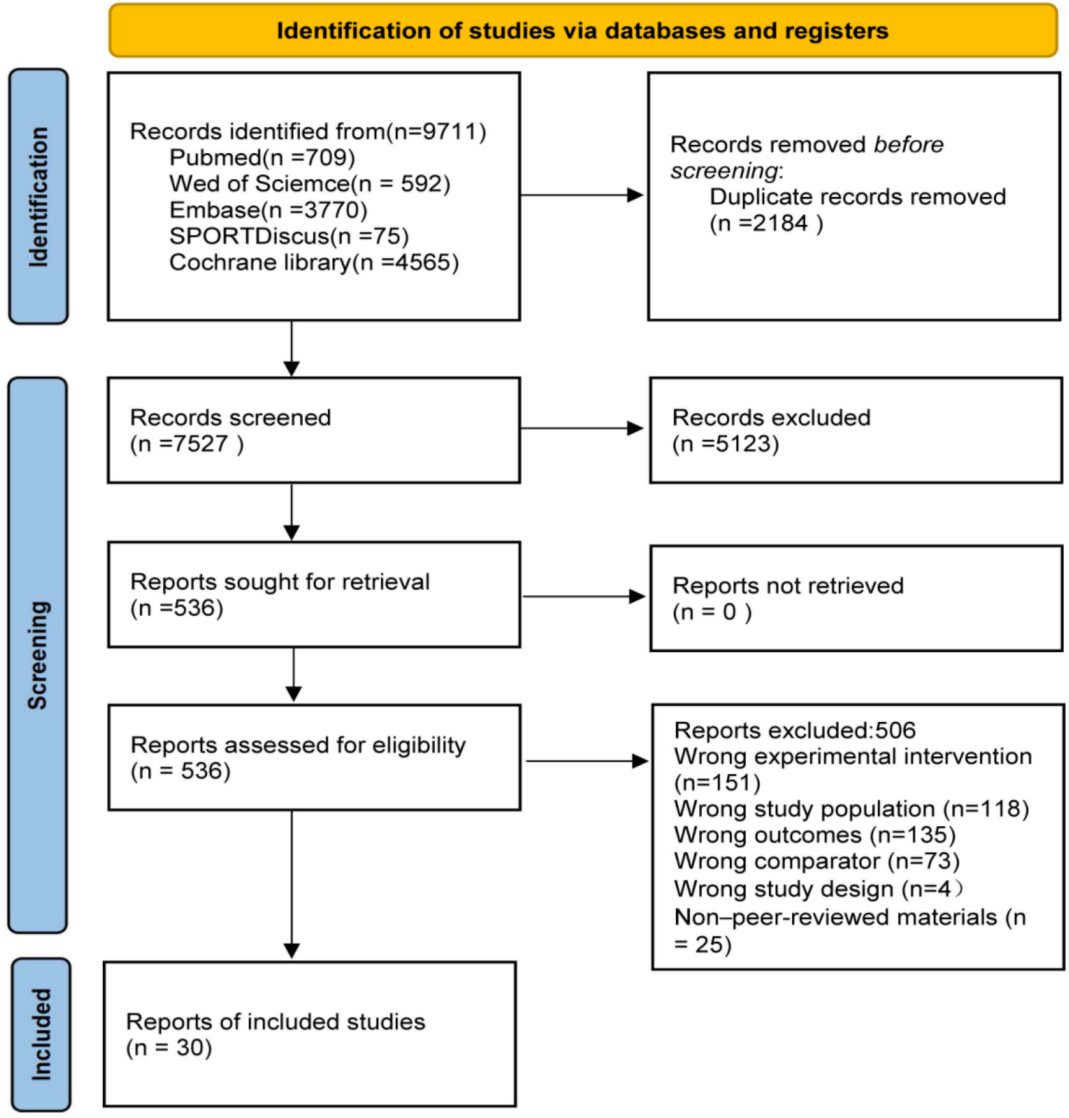
Flowchart of screening process.

A total of 9711 records were retrieved. After removing 2184 duplicates, 7527 unique records were screened by title and abstract, leading to 536 full‐text articles assessed for eligibility. Ultimately, 30 published studies met the inclusion criteria and were incorporated into the quantitative network meta‐analysis. The numbers of records included and excluded at each screening stage are summarized in Figure 1, and detailed characteristics of the included studies are presented in Appendix S2.

### 3.2 Rsk of Bias, Certainty of Evidence, and Consistency

**FIGURE 2:**
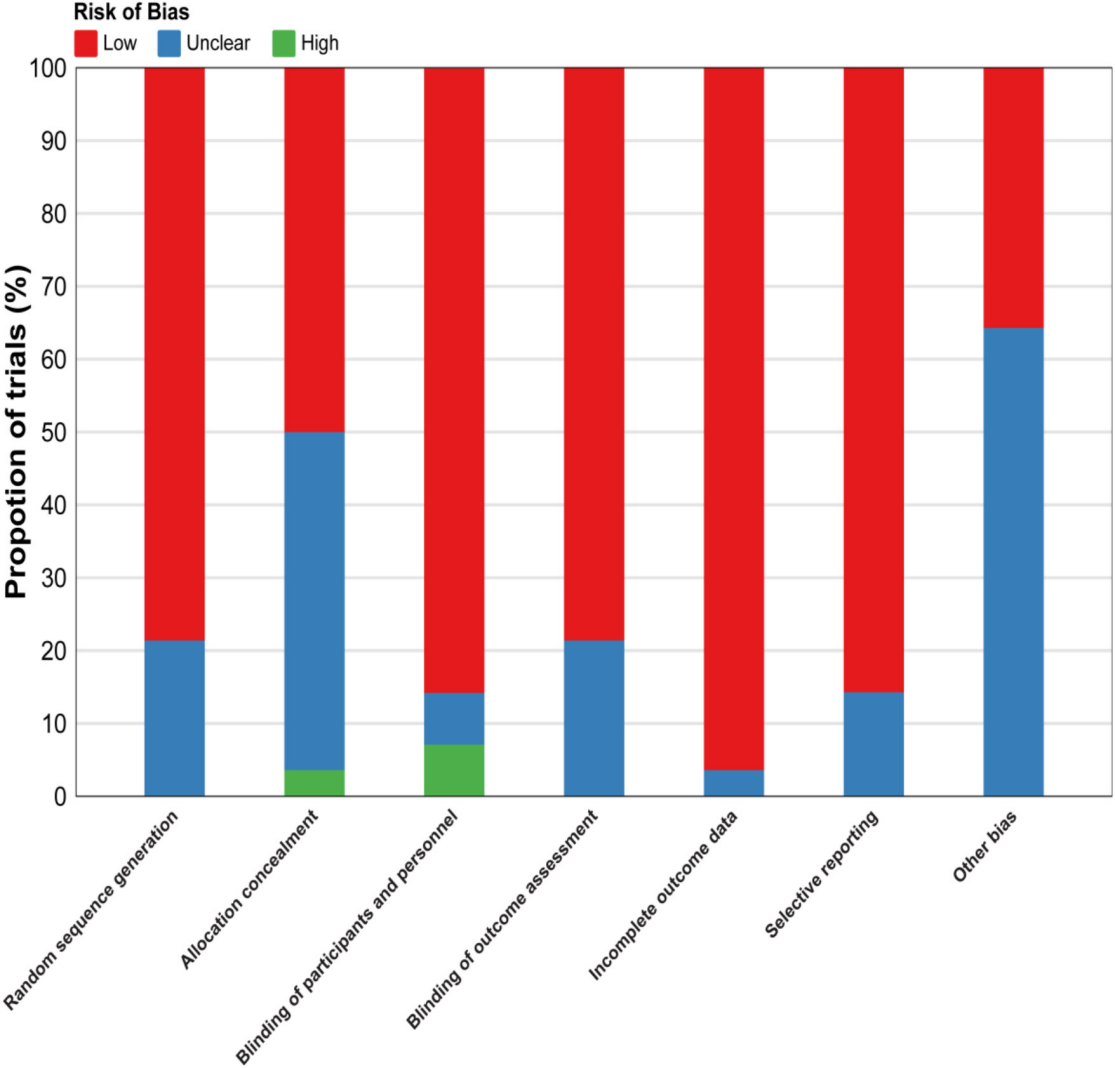
Overall risk of bias presented as percentage of each risk of bias item across all included.

Overall, three studies (10%) were judged to be at low risk of bias, 22 studies (80%) at unclear risk, and three studies (10%) at high risk (Figure 2). The risk‐of‐bias assessment for each trial is provided in Appendix S3. We also evaluated heterogeneity, transitivity, and inconsistency for the present network meta‐analysis (Appendix S4).

Consistency—that is, agreement between direct and indirect evidence—was examined for peak power, mean power, and VO_2_max using node‐splitting analysis; no significant global inconsistency was detected (*p* > 0.05), and local tests likewise revealed no evidence of conflict. For endurance performance, however, the network displayed a star‐shaped configuration with no closed loops, precluding formal consistency testing; accordingly, the results for these two outcomes should be interpreted with caution.

Assessment with the CINeMA framework indicated that most pairwise comparisons yielded evidence of very low to moderate certainty (Appendix S8). All networks satisfied the transitivity assumption, supporting the validity of indirect comparisons (Appendix S8). Funnel plot symmetry and Egger's test showed no signs of publication bias (Appendix S9).

### 3.3 Meta‐Analysis

#### 3.3.1 Peak Power

Seventeen studies encompassing 427 athletes assessed the influence of six dietary supplements on peak power. Moderate‐certainty evidence showed that protein produced the largest improvement relative to control (SMD = 0.85, 95% CI 0.27–1.44; SUCRA = 82.9%), ranking it as the most efficacious intervention. Moderate‐certainty evidence likewise indicated significant gains with creatine (SMD = 0.62, 95% CI 0.20–1.03; SUCRA = 62.5%), HMB (SMD = 0.60, 95% CI 0.28–0.94; SUCRA = 60.7%), and β‐alanine (SMD = 0.58, 95% CI 0.09–1.07; SUCRA = 57.4%).

**FIGURE 3:**
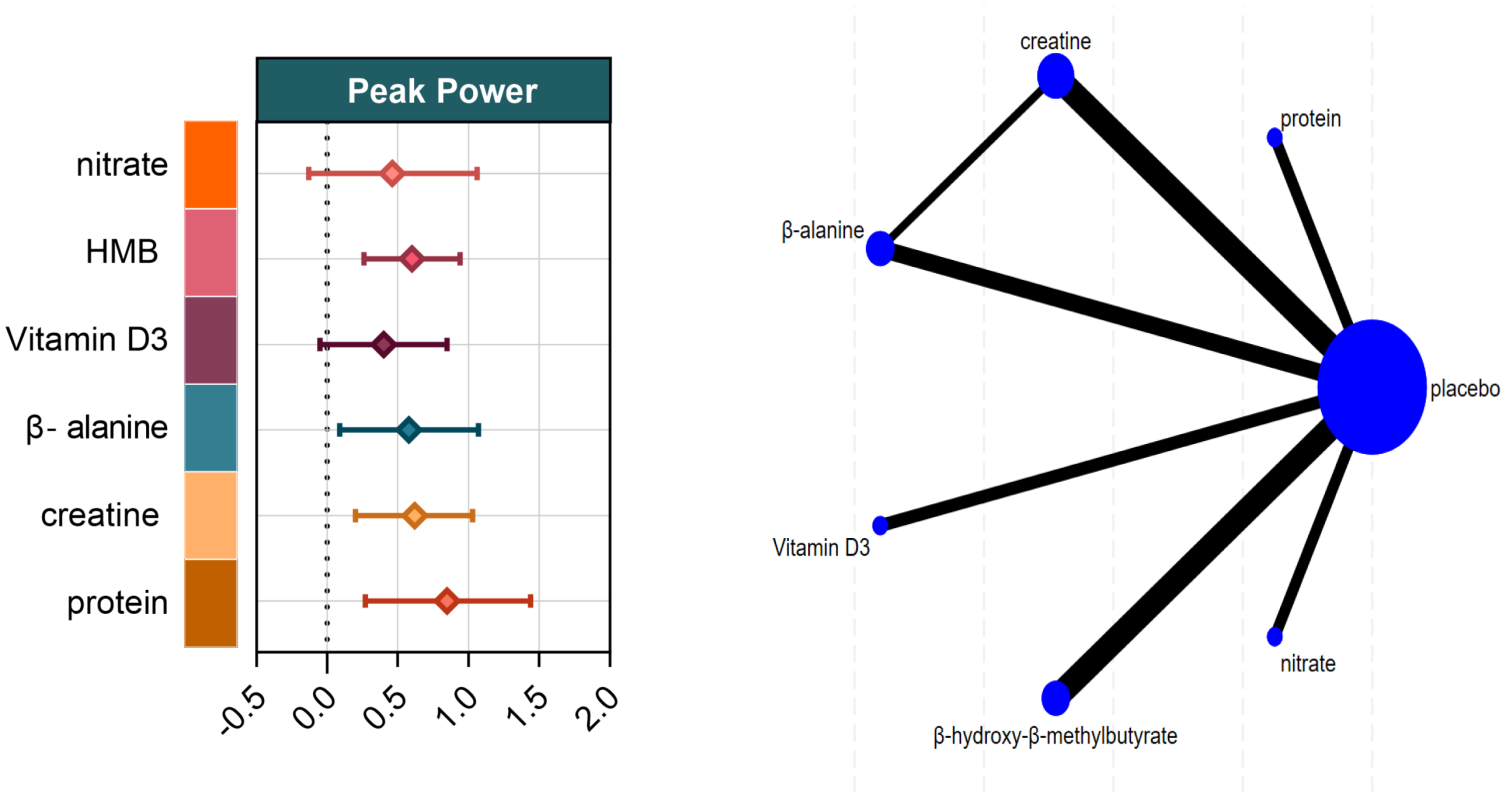
Network plot and forest plot of interventions for Peak power. BA, β‐alanine; HMB, β‐hydroxy‐β‐methylbutyrate.

In contrast, neither vitamin D (SMD = −0.45, 95% CI –1.19 to 0.29; SUCRA = 39.0%) nor nitrate (SMD = 0.46, 95% CI –0.13 to 0.16; SUCRA = 45.4%) exerted a significant effect on peak power (Figures 3 and 7).

#### 3.3.2 Mean Power

**FIGURE 4:**
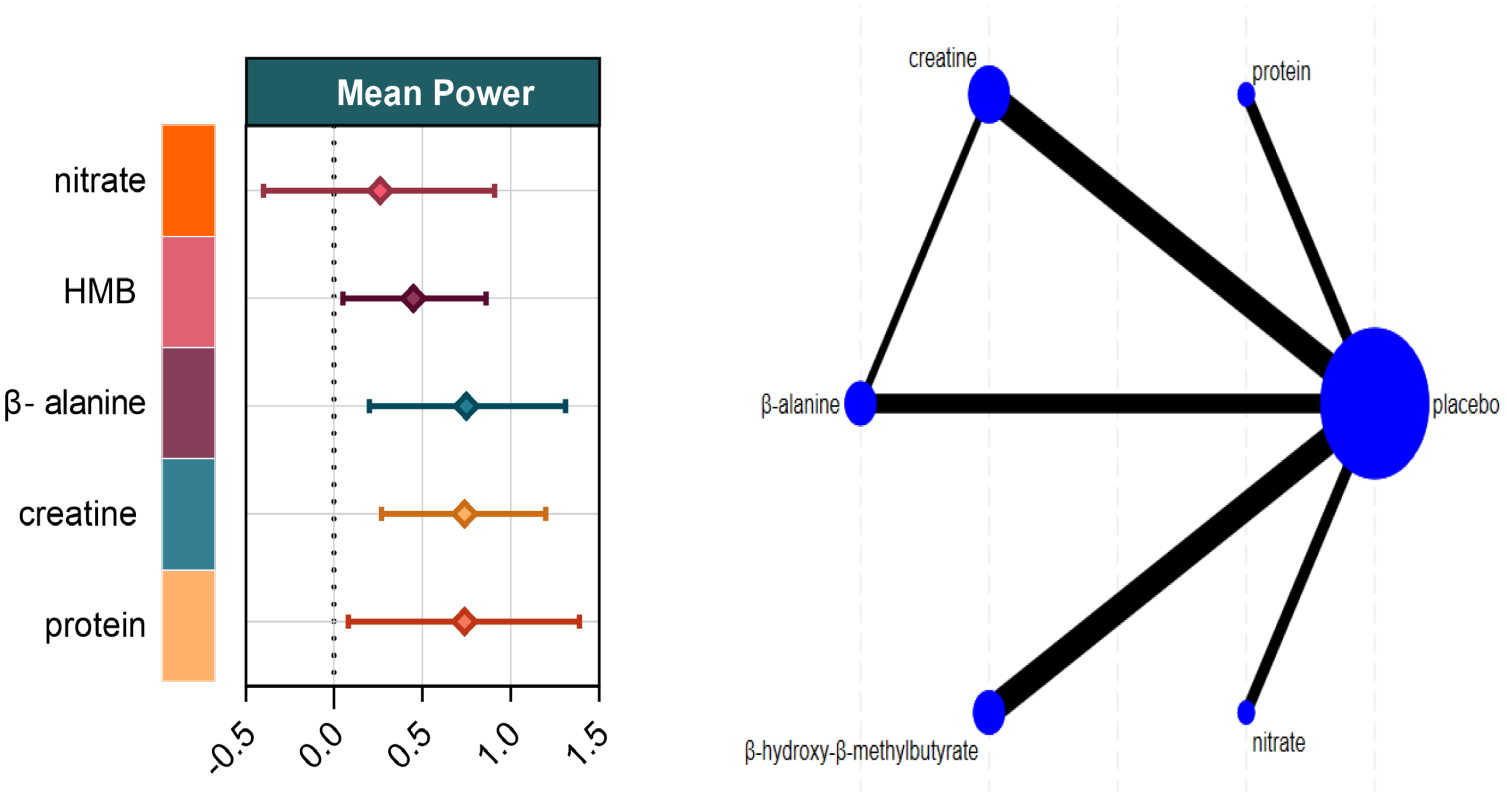
Network plot and forest plot of interventions for Mean Power. BA, β‐alanine; HMB, β‐hydroxy‐β‐methylbutyrate.

Fourteen studies involving 293 athletes investigated the effects of five dietary supplements on mean power. Very‐low‐certainty evidence showed that β‐alanine, protein, and creatine produced the largest improvements versus control (β‐alanine: SMD = 0.75, 95% CI 0.20–1.31; SUCRA = 74.1%; protein: SMD = 0.74, 95% CI 0.27–1.20; SUCRA = 73.9%; creatine: SMD = 0.74, 95% CI 0.08–1.39; SUCRA = 71.0%), ranking them as the most effective interventions. Very‐low‐certainty evidence also indicated that HMB conferred a significant, though smaller, benefit (SMD = 0.45, 95% CI 0.05–0.86; SUCRA = 45.8%). By contrast, nitrate did not significantly affect mean power (SMD = 0.26, 95% CI –0.40 to 0.91; SUCRA = 30.2%) (Figures 4 and 7).

#### 3.3.3 VO_2_max

**FIGURE 5:**
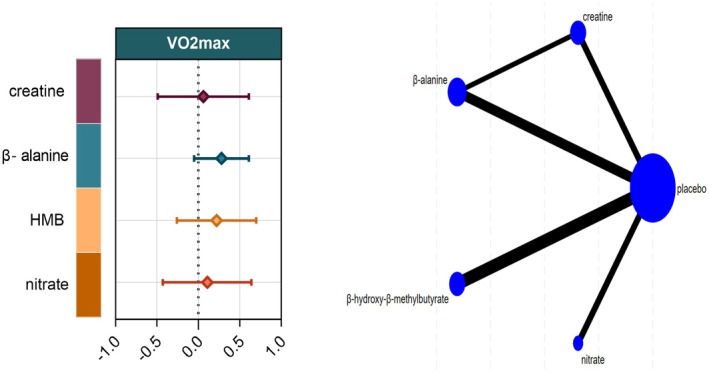
Network plot and forest plot of interventions for VO_2_max. BA, β‐alanine; HMB, β‐hydroxy‐β‐methylbutyrat.

Ten studies comprising 213 athletes examined the effects of four dietary supplements on maximal oxygen uptake. Compared with control, creatine (SMD = 0.11, 95% CI –0.43% to 0.64%), β‐alanine (SMD = 0.22, 95% CI –0.26% to 0.70%), HMB (SMD = 0.28, 95% CI –0.05% to 0.61%), and nitrate (SMD = 0.06, 95% CI –0.49% to 0.61%) produced no statistically significant change in VO₂max. SUCRA values ranked HMB highest (74.4%), followed by β‐alanine (64.2%), creatine (46.3%), and nitrate (39.9%) (Figures 5 and 7).

#### 3.3.4 Endurance Performance

**FIGURE 6:**
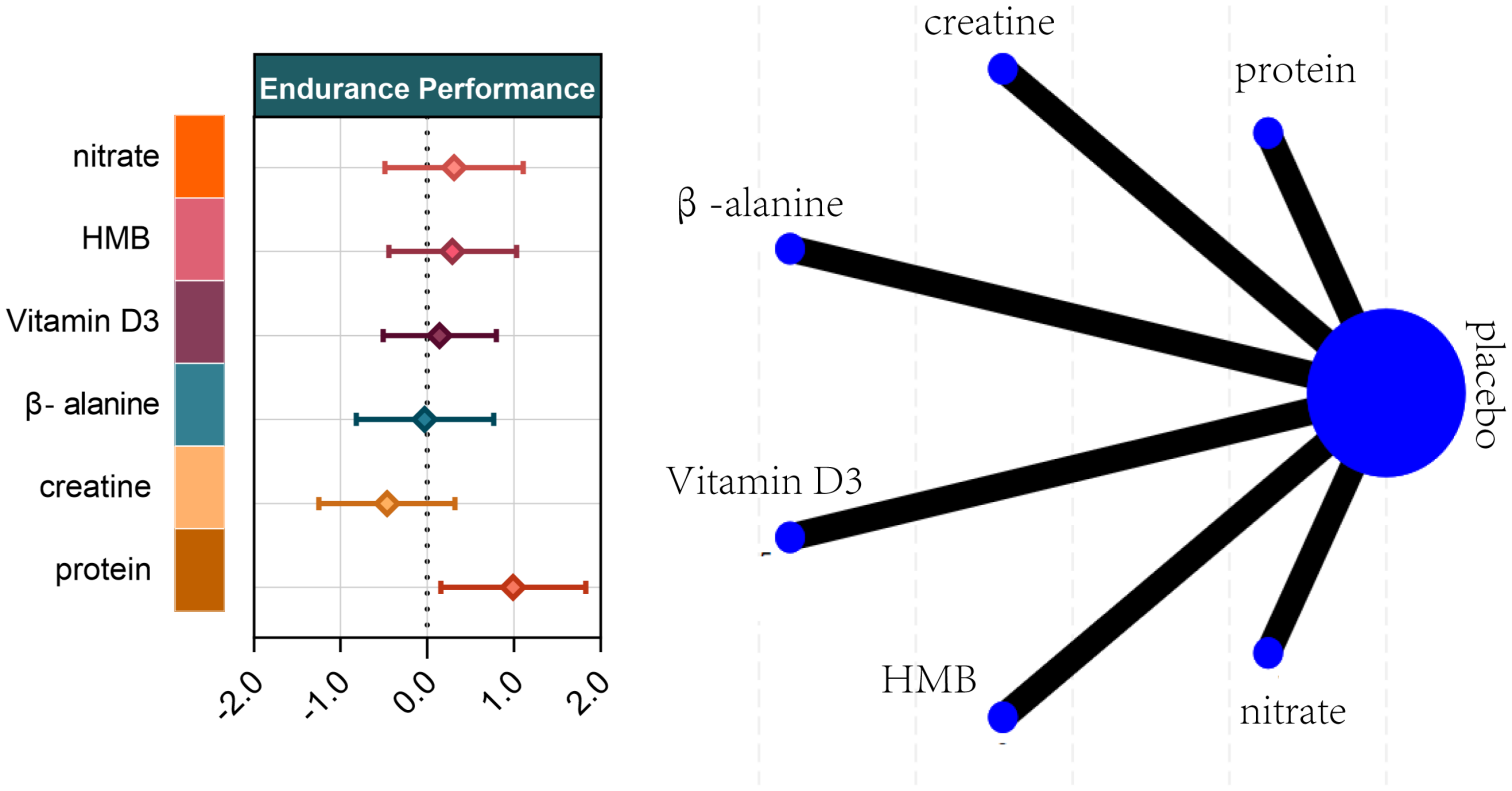
Network plot and forest plot of interventions for Endurance Performance. BA, β‐alanine; HMB, β‐hydroxy‐β‐methylbutyrate.

**FIGURE 7:**
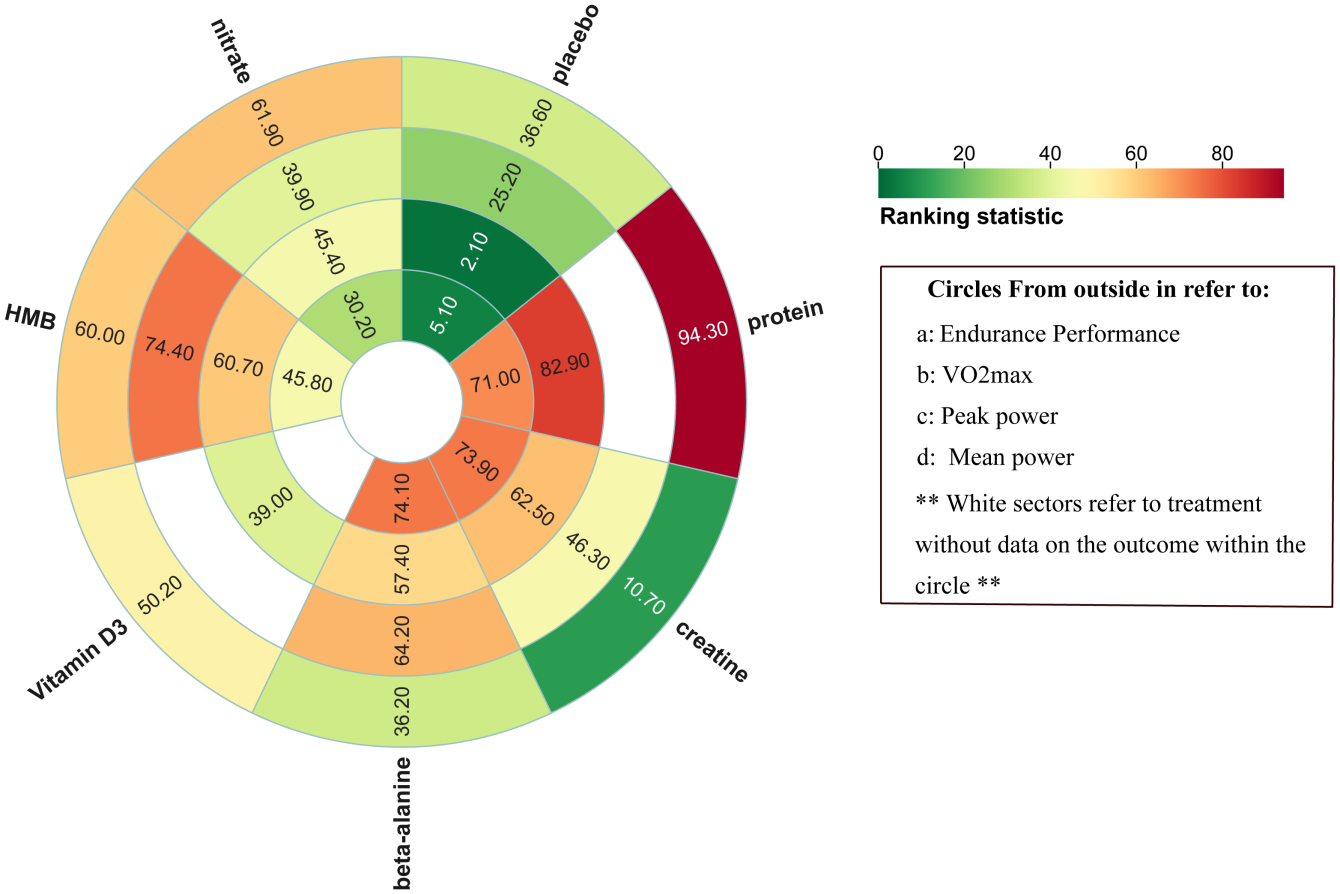
Rank‐heat plot obtained from treatment‐level network meta‐analysis. The rank heat plot presents a summary of P scores (range 0–100) for each intervention across outcomes, where darker shades of red represent more benefit and darker shades of green represent less benefit. β‐alanine, Beta‐alanine; HMB, β‐hydroxy‐β‐methylbutyrate.

Thirteen studies involving 319 athletes evaluated five dietary supplements in relation to endurance performance. Very‐low‐certainty evidence indicated that protein produced the greatest improvement versus control (SMD = 0.99, 95% CI 0.16–1.83; SUCRA = 94.3%), ranking it as the most effective intervention. By contrast, creatine (SMD = −0.46, 95% CI –1.25 to 0.32; SUCRA = 10.7%), vitamin D (SMD = 0.14, 95% CI –0.51 to 0.80; SUCRA = 50.2%), β‐alanine (SMD = −0.03, 95% CI –0.82 to 0.77; SUCRA = 36.2%), HMB (SMD = 0.29, 95% CI –0.44 to 1.03; SUCRA = 60.0%), and nitrate (SMD = 0.31, 95% CI –0.49 to 1.11; SUCRA = 61.9%) showed no significant effect on endurance performance (Figures 6 and 7).

## 4 Discussion

### 4.1 Summary of the Main Findings

Our network meta‐analysis revealed substantial differences in how various dietary supplements, when combined with training, influence different dimensions of athletic performance. In general, we found that protein supplementation yielded the greatest gains in peak anaerobic power (short‐term explosive output), whereas creatine and β‐alanine were most effective for enhancing mean anaerobic power (anaerobic capacity over ~30 s). Notably, none of the supplements produced a statistically significant improvement in maximal aerobic capacity (VO_2_max). For endurance performance outcomes (e.g., time trials or time‐to‐exhaustion tests), improvements were modest or inconsistent—with the exception of protein, which showed an unexpectedly large positive effect. Other supplements like HMB (β‐hydroxy‐β‐methylbutyrate) exhibited moderate benefits in certain anaerobic measures, while nitrate and vitamin D supplementation showed little to no meaningful impact on the performance metrics we examined. The certainty of evidence ranged from moderate to very low for different comparisons, so some results (especially surprisingly large effects) should be interpreted with caution.

### 4.2 Anaerobic Power

Peak and Mean Outputs: For high‐intensity, short‐duration performance, our findings highlight protein and creatine as particularly effective ergogenic aids. Peak power (typically measured by a short all‐out effort, reflecting explosive muscle power) was most improved in athletes receiving protein supplementation. This aligns with prior evidence that adequate protein availability optimizes muscle strength and power adaptations to training (Deng et al. 2025). Protein supports muscle repair and hypertrophy, which likely contributed to the greater gains in explosive power we observed in the protein‐supplemented group. Creatine also conferred a clear benefit for anaerobic power. By expanding intramuscular phosphocreatine stores, creatine enhances the rapid regeneration of ATP during maximal effort exercise, thereby boosting short‐term power output. This effect is well documented; creatine loading typically improves performance in high‐intensity or repetitive sprint tasks by roughly 10%–20% (Kreider 2003). Our results are consistent with this consensus, as creatine‐supplemented athletes showed superior anaerobic performance compared to controls. We also found that HMB had a moderate positive effect on peak power (standardized mean difference ~0.6 in our network). This finding contrasts with the traditional view that HMB offers limited benefit for well‐trained athletes. Indeed, earlier studies in trained populations often reported negligible effects of HMB, especially under non‐overload training conditions (Kraemer et al. 2009). However, more recent research indicates HMB can improve power output when the training stimulus is sufficiently intense or muscle‐damaging. For example, one trial in highly trained athletes reported that 7 weeks of HMB supplementation increased Wingate anaerobic test peak power by a few percent relative to placebo (Zadik et al. 2009). HMB's anticatabolic and recovery‐enhancing properties—reducing exercise‐induced muscle breakdown and soreness—might help athletes maintain higher power output during repeated high‐intensity efforts (Rathmacher et al. 2025). Our findings suggest HMB could be beneficial in scenarios of heavy training stress, although its performance effects are not as pronounced or consistent as those of creatine or protein. In contrast, supplements like nitrate and vitamin D did not improve peak power in our analysis. Nitrate (such as beetroot juice) primarily acts on the nitric oxide pathway to improve muscle efficiency and blood flow, which may not significantly influence instantaneous power generation. Vitamin D is more relevant for muscle function when a deficiency exists; in vitamin D‐replete athletes, extra supplementation is not expected to boost power output. Thus, for maximizing anaerobic peak power, protein and creatine appear to be the most practical and evidence‐supported supplements, with HMB as a potential adjunct, whereas nitrate and vitamin D appear largely ineffectual in this context.

Improvements in mean anaerobic power (e.g., average power over a 30‐s Wingate test, reflecting anaerobic capacity) were observed with β‐alanine, creatine, and protein supplementation. All three showed similar moderate efficacy (SMD ~0.6–0.7 versus placebo). These results make sense given the known mechanisms. β‐Alanine increases muscle carnosine, which buffers intramuscular pH decline during high‐intensity exercise. This helps delay fatigue in efforts lasting from ~30 s up to a few minutes (Mph 2024). While the performance benefit of β‐alanine for a single 30‐s Wingate sprint can be subtle (some studies have found no significant effect on a one‐off sprint; Rathmacher et al. 2025), our aggregated evidence suggests a modest overall improvement in mean power output. It is possible that β‐alanine's buffering becomes more important in the later stages of a 30‐s all‐out test, mitigating the drop‐off in power. Creatine, as discussed, improves the rapid energy supply and is well‐known to increase work done over repeated sprints or short‐duration, maximal efforts (Kreider 2003). Athletes supplementing with creatine can sustain higher power over multiple bouts, which would manifest as a higher mean output in a Wingate test. Similarly, protein supplementation may indirectly enhance anaerobic capacity by facilitating better training recovery and muscle adaptations. Sufficient protein intake supports increases in Type II muscle fiber size and glycolytic enzyme concentrations, potentially allowing athletes to produce and sustain greater power during anaerobic work (Lin et al. 2021). It is worth noting that protein is often thought of in the context of strength and hypertrophy, but our results and other studies indicate it can also augment high‐intensity endurance. For instance, a meta‐analysis on endurance training adaptations found that adding protein led to extra gains in peak power output and faster time‐trial performance, beyond the improvements from endurance training alone (Cintineo et al. 2018). HMB showed a smaller yet statistically significant improvement in mean power in our analysis (SMD ~0.4–0.5). This corresponds with some reports that chronic HMB supplementation can increase total work output in repeated‐sprint tests (Rathmacher et al. 2025). HMB's role in attenuating muscle damage might allow athletes to train harder or recover faster, thereby yielding slight performance benefits in anaerobic capacity over time. Finally, consistent with its limited effect on peak power, dietary nitrate did not significantly affect 30‐s mean power. Anaerobic capacity in a test like the Wingate is predominantly fueled by phosphagens and anaerobic glycolysis, processes which dietary nitrate (working via improved oxygen delivery and efficiency) does not directly enhance. Taken together, to boost short‐duration anaerobic performance, our findings support the use of β‐alanine and creatine as effective options, along with ensuring robust protein intake to maximize training‐mediated gains. HMB may offer additional help in high‐load training scenarios, whereas nitrate is not particularly useful for these specific performance outcomes.

### 4.3 VO_2_max

In contrast to the anaerobic metrics, none of the supplements we evaluated produced a significant improvement in VO_2_max compared to placebo. VO_2_max is strongly determined by cardiovascular capacity, oxygen transport, and mitochondrial density—attributes that are predominantly improved through endurance training and genetic predisposition, rather than acute nutritional interventions. Our results reinforce this principle. Even protein—a supplement that has been reported to modestly enhance VO_2_max gains when combined with endurance training in some studies—showed no significant effect on VO_2_max in our network analysis. This finding underscores that any influence of nutrition on maximal aerobic capacity is likely subtle and context‐dependent, and a robust endurance training stimulus remains the primary driver of VO_2_max improvement. Similarly, ergogenic aids that help with high‐intensity exercise performance failed to move the needle on VO_2_max. For example, creatine, despite its benefits for sprints and strength, has minimal influence on oxidative metabolism and in some cases may slightly impede VO_2_max gains by increasing body mass (an extra weight burden can reduce mass‐normalized oxygen uptake) (Forbes et al. 2023). We observed a small negative effect of creatine on VO_2_max (SMD below zero, though non‐significant), which is consistent with some studies noting that creatine‐loaded athletes sometimes perform worse on endurance measures due to weight (van Loon et al. 2003). Similarly, β‐alanine, by mechanism, targets performance in the 1–4 min range and would not be expected to directly elevate maximal oxygen uptake—our analysis indeed found no VO_2_max benefit from β‐alanine. HMB also did not show a significant impact on VO_2_max in our network, although interestingly it ranked relatively highly in the SUCRA ordering for this outcome. This hints that HMB might have some positive effect that our analysis was underpowered to detect, or that evidence is mixed. Some individual studies have in fact reported improvements in aerobic capacity with HMB. For instance, a placebo‐controlled trial in elite rowers found that 12 weeks of HMB supplementation increased VO_2_max by approximately 2.7 mL·kg^−1^·min^−1^ relative to controls (Durkalec‐Michalski and Jeszka 2015, 2016). Another study noted HMB improved time‐to‐exhaustion and ventilatory threshold in competitive cyclists (Vukovich and Dreifort 2001). Moreover, a recent meta‐analysis (2024) aggregating data from multiple trials concluded that HMB supplementation significantly increases VO_2_max in active individuals, with a pooled SMD around 0.3–0.6 (Holland et al. 2022). These findings suggest that HMB's effect on aerobic fitness might be context‐dependent—potentially more evident in untrained or moderately trained individuals, or under specific training regimens that benefit from HMB's muscle protective effects. In well‐trained athletes (like many in our included studies), the incremental benefit of HMB on VO_2_max may be harder to realize, which could explain why our analysis did not show a clear advantage. In summary, maximal aerobic capacity appears to derive little direct benefit from chronic supplementation with the studied ergogenic aids. When it comes to raising VO_2_max, a sound endurance training program remains irreplaceable, and no supplement in our analysis functioned as a shortcut to a higher VO_2_max (Gao et al. 2021).

### 4.4 Endurance Performance

We examined practical endurance outcomes (such as time trial performance and exercise time‐to‐exhaustion) to gauge real‐world effects on endurance sports. Interestingly, the only supplement that conferred a statistically significant benefit in endurance performance compared to placebo was protein. Athletes who supplemented with protein performed better in endurance tests (e.g., achieving faster time trial times) than those who did not. The magnitude of this effect was relatively large (pooled SMD ~0.9–1.0, favoring protein), which is somewhat surprising in the context of endurance sports where carbohydrates and acute aids are traditionally emphasized more than protein. This result should be interpreted cautiously—the high point estimate might be driven by one or two small studies with large effects—but it does highlight the often underappreciated role of protein in endurance training adaptation. Endurance athletes have substantial protein needs for muscle repair and remodeling, especially given the muscle damage and oxidative stress that can occur with high‐volume training. Sufficient protein availability could improve recovery between sessions and help preserve lean mass, thereby indirectly boosting endurance performance over time (Moore et al. 2014). In fact, recent evidence supports this notion: a 2021 systematic review and meta‐analysis found that adding protein to an endurance training regimen led to greater increases in VO_2_peak and lean mass, and significantly improved time‐trial performance by ~29 s on average, compared to training without extra protein (Lin et al. 2021). Our findings are consistent with those results—protein supplementation modestly but meaningfully enhanced endurance exercise outcomes, likely by augmenting training‐induced adaptations (e.g., mitochondrial enzymes, capillarization, or simply allowing for higher training quality via better recovery) (Kumar et al. 2009). By contrast, most other supplements did not significantly improve endurance performance in our analysis. For example, creatine showed a trivial or slightly negative effect on endurance outcomes (SMD ~–0.4, *p* > 0.05). This aligns with the consensus that creatine, while excellent for power, tends not to help endurance exercise and may even be detrimental for events where a high power‐to‐weight ratio is critical (Forbes et al. 2023). β‐Alanine, despite clear benefits for medium‐duration high‐intensity efforts, did not translate into detectable improvements in longer endurance performance (e.g., sustained efforts of many minutes to an hour). Marathon runners or cyclists likely see more benefit from β‐alanine in the context of a final sprint or high‐intensity surge rather than an overall time trial outcome—any minor advantage in the latter is hard to capture, and indeed our results found no significant effect. HMB supplementation, as noted, had no significant aggregate effect on endurance performance in our network, even though its point estimate was slightly positive. While HMB could theoretically improve endurance by enhancing recovery (thus enabling better training consistency) or by increasing the use of fat as fuel (as some mechanistic studies suggest; Stancliffe 2012; Vukovich and Dreifort 2001), the current evidence in athletes is mixed. Some studies have observed that HMB can prolong time to fatigue and improve endurance capacity under specific conditions (Vukovich and Dreifort 2001), but others show minimal impact, especially in well‐trained subjects. A 2024 meta‐analysis reported a moderate improvement in endurance performance (pooled SMD ~0.38) with HMB (Holland et al. 2022), suggesting there is potential ergogenic value. Nevertheless, given that our NMA did not show a clear benefit and considering the variability in literature, we infer that HMB is not a reliably effective endurance aid for trained athletes, except perhaps in scenarios of nutritional deficit or very intense training stress where its anti‐catabolic effects prevent performance decrements. Lastly, dietary nitrates deserve discussion. Nitrate supplementation (commonly via beetroot juice) has been a popular strategy to improve endurance exercise efficiency by boosting nitric oxide. Mechanistically, nitrates can reduce the oxygen cost of submaximal exercise and improve muscle blood flow, which in some studies leads to prolonged time‐to‐exhaustion at a given workload (Holland et al. 2022). However, our analysis found no significant improvement from chronic nitrate supplementation on measured endurance performance outcomes. Nitrates ranked second in SUCRA for endurance, hinting at a possible moderate benefit, but the effect was not statistically significant. This outcome is in line with prior meta‐analyses that have noted a discrepancy in nitrate's effects: while nitrates consistently extend time‐to‐exhaustion tests (on average by ~25–30 s) (Gao et al. 2021), they often do not significantly improve actual time‐trial results (timed performance over a set distance) (Gao et al. 2021). Our results echo this pattern—nitrates help an athlete push a bit longer at a given intensity, but may not translate into faster race times, especially for well‐trained athletes. One reason is that highly trained endurance athletes are thought to be less responsive to nitrates; their nitric oxide pathways might already be near‐optimized from training, leaving less room for improvement (Bescós et al. 2011; Bond et al. 2012; Wickham and Spriet 2019). Additionally, many of the studies in our analysis involved daily nitrate supplementation over weeks; some evidence suggests the performance gains from nitrates are more pronounced in acute, pre‐competition use rather than as a continuous training supplement (possibly due to the development of tolerance with prolonged use) (Domínguez et al. 2017). In summary, endurance performance appears most improved by ensuring adequate protein intake, whereas supplements like creatine, β‐alanine, HMB, and chronic nitrate loading did not show robust efficacy for endurance outcomes in the aggregated data. Endurance athletes should thus focus on training and nutrition fundamentals, using supplements strategically rather than expecting drastic performance changes from any single supplement.

### 4.5 Practical Implications

From a practical perspective, our findings underscore the importance of targeted nutrition strategies aligned with specific athletic goals (Deng et al. 2025). There is no one‐size‐fits‐all supplement for performance; instead, athletes and coaches should choose supplements that address the particular demands of their sport or training phase. For athletes who rely on explosive power and anaerobic performance—for example, sprinters, weightlifters, or team‐sport athletes who perform repeated high‐intensity bursts—creatine supplementation stands out as a highly effective aid. It can reliably increase peak power, repeated sprint ability, and strength gains, thereby enabling greater training quality and performance in explosive tasks (Deng et al. 2025; Kreider et al. 2017). High‐quality protein intake is equally crucial for these athletes, as it maximizes muscle repair and adaptation from resistance or sprint training. Our results suggest that ample protein (through diet or supplementation) helps translate training into improved power and possibly even endurance, making it a foundational component of any athlete's nutrition plan (Lin et al. 2021). In periods of very intense training or when muscle recovery is a limiting factor, athletes might consider adding HMB as a recovery aid. HMB could be beneficial during training blocks that carry a high risk of muscle damage (such as preseason strength conditioning or overreaching cycles), as some research indicates it can reduce muscle protein breakdown and soreness, allowing for better maintenance of power output over successive sessions (Wilson et al. 2008). However, because HMB's performance benefits are not universally observed, athletes should individually assess its effectiveness. For those engaged in middle‐distance or repeated‐sprint sports—activities requiring a blend of anaerobic power and capacity (e.g., 400–800 m runners, swimmers, cyclists in sprint events, or field athletes who perform intermittent sprints)—a combination of β‐alanine and creatine may be particularly useful. These two supplements work through complementary mechanisms: β‐alanine improves the muscle's intracellular buffering capacity, delaying fatigue in high‐intensity efforts beyond ~60 s (Mph 2024), while creatine amplifies the immediate energy supply for short bursts of effort (Kreider et al. 2017). Using them in tandem (along with a solid training program) can help an athlete sustain higher power outputs for longer during repeated high‐intensity bouts. Indeed, practitioners have suggested periodizing β‐alanine and creatine together in training phases aimed at boosting anaerobic capacity and explosive endurance (Deng et al. 2025). Again, protein underpins all these efforts by supporting muscle function and recovery, so athletes in these events should not neglect protein intake amidst their focus on performance supplements. On the other hand, for athletes whose primary focus is aerobic endurance—such as long‐distance runners, cyclists, triathletes, or rowers—our findings emphasize that core training and nutrition are paramount. No chronic supplement in our analysis produced a dramatic improvement in endurance race performance or VO_2_max, which means athletes should first ensure they are optimizing well‐established factors: appropriate endurance training intensity and volume, sufficient rest, and a diet rich in carbohydrates for fuel and balanced in micronutrients. Within that context, protein remains important to facilitate recovery from training and to help prevent losses of muscle mass during heavy endurance workloads (Lin et al. 2021). Endurance athletes might not traditionally prioritize protein as much as strength athletes, but the evidence suggests that doing so (e.g., through protein supplements or protein‐rich foods) can further enhance endurance performance gains over time (Lin et al. 2021). Addressing specific nutritional deficiencies is also a key practical point—for instance, iron deficiency or vitamin D deficiency can impair endurance capacity, and correcting those via supplementation can thus indirectly improve performance (even though our results showed that supplementing an already sufficient athlete with vitamin D had no added benefit). When it comes to supplements like nitrates, endurance athletes should consider how and when to use them. Our analysis indicates that routine daily nitrate supplementation during training had limited impact on performance outcomes. However, this does not discount the value of nitrates as an acute ergogenic aid. Many athletes and coaches report success with loading nitrates in the days leading up to, or hours before, a competition to gain a small edge in exercise efficiency (Gao et al. 2021). The lack of chronic effect in our study suggests that if an athlete is going to use nitrates, they may derive more benefit from timing the intake strategically around key events or hard workouts rather than taking it continuously. Similarly, other well‐supported acute aids like caffeine and sodium bicarbonate were outside the scope of our review (since we focused on ≥ 2‐week interventions), but they remain important options in the sports nutrition toolkit for competition day. Ultimately, the practical message is that nutrition and supplementation should be periodized and individualized. Athletes should periodically re‐evaluate their supplement regimen to match their training cycle (as also advocated by recent comprehensive reviews; Deng et al. 2025), and any supplement should be tested in training first to ensure it confers the expected benefit without adverse effects. The principle of “train high, compete higher” can be applied to nutrition: integrate supplements in training to enhance adaptations or acute performance as needed, but rely on the fundamentals of training adaptations as the primary driver of improvement. By aligning the choice of supplement with the specific performance goal—be it increasing sprint power, buffering fatigue in anaerobic repeats, or recovering from back‐to‐back training sessions—athletes can maximize the positive impact of nutrition on their performance while avoiding superfluous or counterproductive supplementation.

### 4.6 Strengths and Limitations

This review provides, to our knowledge, the most comprehensive comparative analysis to date of how different supplements, alongside training, affect multiple performance facets in trained athletes. Using a network meta‐analytic approach, we were able to integrate direct and indirect evidence across trials and even rank the effectiveness of supplements that have rarely been compared head‐to‐head. This approach yields a broad, practice‐oriented perspective that is highly valuable for coaches and sports nutritionists making decisions across many potential interventions. We also improved real‐world relevance by including only multi‐week interventions in trained participants, reflecting how athletes typically use supplements during training (as opposed to acute one‐off usage). Our methodology was rigorous—we preregistered the review, performed an exhaustive literature search, and assessed risk of bias for each study. Additionally, by grading the confidence in estimates via the CINeMA/GRADE framework, we have transparently conveyed the strength of evidence for each outcome. These strengths enhance the robustness and practical utility of our conclusions. That said, there are important limitations to acknowledge. The overall quality and consistency of evidence were variable and often low. Only a small fraction of the included RCTs were at low risk of bias (roughly 9%); the majority had at least some methodological concerns or modest sample sizes. Consequently, many of our network estimates are based on low or very low certainty evidence, and true effects might be smaller or differ in direction (Deng et al. 2025). For example, the surprisingly large effect of protein on endurance performance might be an overestimate driven by a couple of small studies—future research could find a more modest benefit. We urge caution not to overgeneralize singular findings without considering their evidence certainty. Heterogeneity among the included studies is another concern. The trials encompassed a wide range of sports (endurance, strength, team sports), training protocols (from resistance training to interval training), and participant characteristics (from collegiate athletes to military personnel). They also differed in supplement dosages and durations. While we used statistical models to account for some of these differences, such diversity inevitably introduces noise and may limit the applicability of any one finding to a specific sport scenario. In some cases, it may explain why certain supplements were beneficial in some studies but not in others. For instance, elite endurance athletes responded differently to nitrate than recreational athletes in some analyses (Bescós et al. 2011; Bond et al. 2012; Wickham and Spriet 2019), and HMB's effect varied with training intensity—nuances that a broad analysis can only partially capture. Outcome definitions also varied; what one study defined as “endurance performance” (e.g., a timed trial) another defined as “time to exhaustion,” which, while related, are not identical measures. This variability adds uncertainty to the pooled effects and SUCRA rankings we reported. Another limitation is that we focused on chronic supplementation and thus excluded many studies on acute supplementation strategies (e.g., a single dose of caffeine before exercise). As a result, our conclusions apply to supplements taken regularly during training periods, not necessarily to in‐competition or acute use of ergogenics. Practitioners should therefore interpret our findings as guidance for training‐enhancement nutrition, and recognize that separate evidence exists for acute performance aids. Furthermore, we did not explicitly examine dose–response relationships for each supplement due to insufficient data; it is possible that higher or lower doses than those typically used in studies could yield different effects or side‐effect profiles. We also did not delve into the long‐term safety of these supplementation practices—all the supplements we included are generally regarded as safe at recommended doses, but athletes should always be mindful of quality control and any sport‐specific anti‐doping regulations. Lastly, although we covered many common supplements, we did not include certain ergogenic aids (such as caffeine, bicarbonate, tart cherry, branched‐chain amino acids, etc.) either because they did not meet our inclusion criteria or there were not enough consistent data in the literature for our analysis. These exclusions mean our comparative rankings are not exhaustive. Future network meta‐analyses should incorporate a broader array of supplements as new studies become available, and should also strive for more high‐quality, large‐sample trials—especially for supplements like HMB or vitamin D where current evidence is limited—to increase confidence in the findings. Despite these limitations, we believe our review offers valuable insights by synthesizing a vast body of research into a cohesive comparative framework.

In summary, this systematic review and network meta‐analysis maps out the landscape of popular training‐phase supplements and their effects on athletic performance. The evidence reinforces several well‐established concepts—for example, creatine's robust ability to enhance anaerobic power, and the fact that dietary nitrate, while improving exercise efficiency, does not boost VO_2_max or reliably speed up time trials. At the same time, our findings reveal some notable patterns that add nuance to sports nutrition knowledge. Most prominently, protein supplementation emerged as a broadly beneficial strategy, not only for strength and muscle mass gains but also for improving endurance outcomes—underscoring protein's role as a cornerstone for all athletes, including endurance. Meanwhile, targeted supplements like β‐alanine and HMB showed value primarily under specific conditions (high‐intensity efforts and heavy training stress, respectively), and proved less universally effective than staples like protein or creatine. Overall, our results provide evidence‐informed guidance for athletes and practitioners: supplements such as protein and creatine appear most effective when aligned with specific performance objectives, use other supplements selectively where a clear mechanism and need exist, and maintain realistic expectations about the magnitude of performance changes. It is also critical to apply these findings in context—individual responses can vary, and factors such as an athlete's training status, baseline diet, and particular sport demands should inform supplement choices. Lastly, we emphasize that current evidence is still evolving. There remain gaps and controversies (for instance, the true extent of HMB's benefits, or the best ways to deploy nitrates for competition). As new research continues to emerge, especially well‐controlled trials on diverse athlete populations, our understanding of how to optimally combine training and nutrition will sharpen. In the pursuit of athletic excellence, a tailored, evidence‐informed approach to supplementation—one that respects both the proven fundamentals and the individual athlete's context—will likely yield the best performance outcomes.

## 5 Conclusion

This network meta‐analysis indicates that the ergogenic effects of training‐phase supplements are outcome‐specific: protein is consistently associated with meaningful improvements in peak anaerobic power and endurance performance; β‐alanine and creatine show comparable, statistically significant gains in mean anaerobic power; creatine (and HMB in some comparisons) also enhances peak power; none of the supplements show a clear effect on VO_2_max. In practice, protein can be prioritized during strength‐oriented or high‐load phases; β‐alanine is a practical option when sustaining high‐intensity output is the goal; and creatine remains a strong choice for sprint‐focused work. Supplement use should be periodized and individualized, aligned with sport demands and phase of training. These inferences primarily reflect multi‐week (typically ≤ 8–12 weeks) interventions in young, trained adults; generalization to other populations or longer interventions should be made cautiously and supported by larger, well‐controlled trials with standardized outcomes.

## Author Contributions

**Beiwang Deng:** conceptualization, methodology, investigation, formal analysis, data curation, writing – original draft, writing – review and editing. **Haoran Li:** investigation, data curation, formal analysis, visualization, writing – review and editing. **Cheng Chen:** resources, investigation, methodology, software, writing – review and editing. **Jiaxin He:** supervision, project administration, validation, writing – review and editing. **Wenfeng Zhang:** supervision, resources, validation, funding acquisition, writing – review and editing. **Hong Lin:** supervision, project administration, resources, funding acquisition, writing – review and editing.

## Funding

The authors have nothing to report.

## Ethics Statement

This review was conducted according to the PRISMA (Preferred Reporting Items for Systematic Reviews and Meta‐Analyses) guidelines.

## Consent

The authors have nothing to report.

## Conflicts of Interest

The authors declare no conflicts of interest.

## Supporting information


**Appendixes S1–S9:** fsn371243‐sup‐0001‐AppendixesS1‐S9.docx.

## Data Availability

The data that support the findings of this study are available from the corresponding authors upon reasonable request.

## References

[fsn371243-bib-0001] Aguiló, A. , P. Tauler , A. Sureda , N. Cases , J. Tur , and A. Pons . 2007. “Antioxidant Diet Supplementation Enhances Aerobic Performance in Amateur Sportsmen.” Journal of Sports Sciences 25, no. 11: 1203–1210. 10.1080/02640410600951597. 17654232

[fsn371243-bib-0002] Bellar, D. , A. Hatchett , L. Judge , M. Breaux , and L. Marcus . 2015. “The Relationship of Aerobic Capacity, Anaerobic Peak Power and Experience to Performance in CrossFit Exercise.” Biology of Sport 32, no. 4: 315–320. 10.5604/20831862.1174771. 26681834 PMC4672163

[fsn371243-bib-0003] Bescós, R. , F. A. Rodríguez , X. Iglesias , M. D. Ferrer , E. Iborra , and A. Pons . 2011. “Acute Administration of Inorganic Nitrate Reduces VO (2peak) in Endurance Athletes.” Medicine and Science in Sports and Exercise 43, no. 10: 1979–1986. 10.1249/MSS.0b013e318217d439. 21407132

[fsn371243-bib-0004] Bond, H. , L. Morton , and A. J. Braakhuis . 2012. “Dietary Nitrate Supplementation Improves Rowing Performance in Well‐Trained Rowers.” International Journal of Sport Nutrition and Exercise Metabolism 22, no. 4: 251–256. 10.1123/ijsnem.22.4.251. 22710356

[fsn371243-bib-0005] Bosquet, L. , L. Léger , and P. Legros . 2002. “Methods to Determine Aerobic Endurance.” Sports Medicine 32, no. 11: 675–700. 10.2165/00007256-200232110-00002. 12196030

[fsn371243-bib-0006] Burke, R. , A. Piñero , M. Coleman , et al. 2023. “The Effects of Creatine Supplementation Combined With Resistance Training on Regional Measures of Muscle Hypertrophy: A Systematic Review With Meta‐Analysis.” Nutrients 15, no. 9: 2116. 10.3390/nu15092116. 37432300 PMC10180745

[fsn371243-bib-0007] Castell, L. M. , S. J. Stear , and L. M. Burke . 2015. Nutritional Supplements in Sport, Exercise and Health: An AZ Guide. Routledge. https://books.google.com/books?hl=zh‐CN&lr=&id=U4BKCAAAQBAJ&oi=fnd&pg=PP1&dq=Nutritional+supplements+in+Sport,+exercise+and+Health:+an+A‐Z+guide&ots=CKIFbTwFwR&sig=cZJnoDJcGTzEsQGJAQF7fiBiYpU.

[fsn371243-bib-0008] Chandler, J. , M. Cumpston , T. Li , M. J. Page , and V. Welch . 2019. Cochrane Handbook for Systematic Reviews of Interventions. 4th ed. Wiley. https://dariososafoula.wordpress.com/wp‐content/uploads/2017/01/cochrane‐handbook‐for‐systematic‐reviews‐of‐interventions‐2019‐1.pdf. 10.1002/14651858.ED000142 PMC10284251 31643080

[fsn371243-bib-0009] Cintineo, H. P. , M. A. Arent , J. Antonio , and S. M. Arent . 2018. “Effects of Protein Supplementation on Performance and Recovery in Resistance and Endurance Training.” Frontiers in Nutrition 5: 83. 10.3389/fnut.2018.00083. 30255023 PMC6142015

[fsn371243-bib-0010] Cohen, J. 2013. Statistical Power Analysis for the Behavioral Sciences. Routledge. https://www.taylorfrancis.com/books/mono/10.4324/9780203771587/statistical‐power‐analysis‐behavioral‐sciences‐jacob‐cohen.

[fsn371243-bib-0011] Cronin, J. , and G. Sleivert . 2005. “Challenges in Understanding the Influence of Maximal Power Training on Improving Athletic Performance.” Sports Medicine 35, no. 3: 213–234. 10.2165/00007256-200535030-00003. 15730337

[fsn371243-bib-0012] Daher, J. , M. Mallick , and D. El Khoury . 2022. “Prevalence of Dietary Supplement Use Among Athletes Worldwide: A Scoping Review.” Nutrients 14, no. 19: 4109. 10.3390/nu14194109. 36235761 PMC9570738

[fsn371243-bib-0013] de Oliveira, L. F. , E. Dolan , P. A. Swinton , et al. 2022. “Extracellular Buffering Supplements to Improve Exercise Capacity and Performance: A Comprehensive Systematic Review and Meta‐Analysis.” Sports Medicine 52, no. 3: 505–526. 10.1007/s40279-021-01575-x. 34687438

[fsn371243-bib-0014] Deng, B. , R. Yan , T. He , et al. 2025. “Effects of Different Dietary Supplements Combined With Conditioning Training on Muscle Strength, Jump Performance, Sprint Speed, and Muscle Mass in Athletes: A Systematic Review and Network Meta‐Analysis.” Frontiers in Nutrition 12: 1636970. 10.3389/fnut.2025.1636970. 40717998 PMC12295849

[fsn371243-bib-0015] Dias, S. , N. J. Welton , A. J. Sutton , D. M. Caldwell , G. Lu , and A. E. Ades . 2013. “Evidence Synthesis for Decision Making 4: Inconsistency in Networks of Evidence Based on Randomized Controlled Trials.” Medical Decision Making 33, no. 5: 641–656. 10.1177/0272989X12455847. 23804508 PMC3704208

[fsn371243-bib-0016] Domínguez, R. , M. V. Garnacho‐Castaño , E. Cuenca , et al. 2017. “Effects of Beetroot Juice Supplementation on a 30‐s High‐Intensity Inertial Cycle Ergometer Test.” Nutrients 9, no. 12: 1360. 10.3390/nu9121360. 29244746 PMC5748810

[fsn371243-bib-0017] Durkalec‐Michalski, K. , and J. Jeszka . 2015. “The Efficacy of a β‐Hydroxy‐β‐Methylbutyrate Supplementation on Physical Capacity, Body Composition and Biochemical Markers in Elite Rowers: A Randomised, Double‐Blind, Placebo‐Controlled Crossover Study.” Journal of the International Society of Sports Nutrition 12, no. 1: 31. 10.1186/s12970-015-0092-9. 26225130 PMC4518594

[fsn371243-bib-0018] Durkalec‐Michalski, K. , and J. Jeszka . 2016. “The Effect of HMB on Aerobic Capacity and Body Composition in Trained Athletes.” Journal of Strength and Conditioning Research 30, no. 9: 2617–2626. 10.1519/JSC.0000000000001361. 26849784

[fsn371243-bib-0019] Forbes, S. C. , D. G. Candow , J. H. F. Neto , et al. 2023. “Creatine Supplementation and Endurance Performance: Surges and Sprints to Win the Race.” Journal of the International Society of Sports Nutrition 20, no. 1: 2204071. 10.1080/15502783.2023.2204071. 37096381 PMC10132248

[fsn371243-bib-0020] Gao, C. , S. Gupta , T. Adli , et al. 2021. “The Effects of Dietary Nitrate Supplementation on Endurance Exercise Performance and Cardiorespiratory Measures in Healthy Adults: A Systematic Review and Meta‐Analysis.” Journal of the International Society of Sports Nutrition 18, no. 1: 55. 10.1186/s12970-021-00450-4. 34243756 PMC8268374

[fsn371243-bib-0021] Guest, N. S. , T. A. VanDusseldorp , M. T. Nelson , et al. 2021. “International Society of Sports Nutrition Position Stand: Caffeine and Exercise Performance.” Journal of the International Society of Sports Nutrition 18: 1. 10.1186/s12970-020-00383-4. 33388079 PMC7777221

[fsn371243-bib-0022] Higgins, J. P. , J. Savović , M. J. Page , R. G. Elbers , and J. A. Sterne . 2019. “Assessing Risk of Bias in a Randomized Trial.” In Cochrane Handbook for Systematic Reviews of Interventions, 205–228. John Wiley & Sons, Ltd. 10.1002/9781119536604.ch8.

[fsn371243-bib-0023] Higgins, J. P. , J. A. Sterne , J. Savovic , et al. 2016. “A Revised Tool for Assessing Risk of Bias in Randomized Trials.” Cochrane Database of Systematic Reviews 10, no. Suppl 1: 29–31.

[fsn371243-bib-0024] Holland, B. M. , B. M. Roberts , J. W. Krieger , and B. J. Schoenfeld . 2022. “Does HMB Enhance Body Composition in Athletes? A Systematic Review and Meta‐Analysis.” Journal of Strength & Conditioning Research 36, no. 2: 585–592. 10.1519/JSC.0000000000003461. 31868817

[fsn371243-bib-0025] Hutton, B. , G. Salanti , D. M. Caldwell , et al. 2015. “The PRISMA Extension Statement for Reporting of Systematic Reviews Incorporating Network Meta‐Analyses of Health Care Interventions: Checklist and Explanations.” Annals of Internal Medicine 162, no. 11: 777–784. 10.7326/M14-2385. 26030634

[fsn371243-bib-0026] Knuiman, P. , L. J. C. van Loon , J. Wouters , M. Hopman , and M. Mensink . 2019. “Protein Supplementation Elicits Greater Gains in Maximal Oxygen Uptake Capacity and Stimulates Lean Mass Accretion During Prolonged Endurance Training: A Double‐Blind Randomized Controlled Trial.” American Journal of Clinical Nutrition 110, no. 2: 508–518. 10.1093/ajcn/nqz093. 31240303

[fsn371243-bib-0027] Kraemer, W. J. , D. L. Hatfield , J. S. Volek , et al. 2009. “Effects of Amino Acids Supplement on Physiological Adaptations to Resistance Training.” Medicine and Science in Sports and Exercise 41, no. 5: 1111–1121. 10.1249/MSS.0b013e318194cc75. 19346975

[fsn371243-bib-0028] Kreider, R. B. 2003. “Effects of Creatine Supplementation on Performance and Training Adaptations.” Molecular and Cellular Biochemistry 244, no. 1: 89–94. 10.1023/A:1022465203458. 12701815

[fsn371243-bib-0029] Kreider, R. B. , D. S. Kalman , J. Antonio , et al. 2017. “International Society of Sports Nutrition Position Stand: Safety and Efficacy of Creatine Supplementation in Exercise, Sport, and Medicine.” Journal of the International Society of Sports Nutrition 14, no. 1: 1–18. 10.1186/s12970-017-0173-z. 28615996 PMC5469049

[fsn371243-bib-0030] Kumar, V. , P. Atherton , K. Smith , and M. J. Rennie . 2009. “Human Muscle Protein Synthesis and Breakdown During and After Exercise.” Journal of Applied Physiology 106, no. 6: 2026–2039. 10.1152/japplphysiol.91481.2008. 19164770

[fsn371243-bib-0031] Lin, Y.‐N. , T.‐T. Tseng , P. Knuiman , et al. 2021. “Protein Supplementation Increases Adaptations to Endurance Training: A Systematic Review and Meta‐Analysis.” Clinical Nutrition 40, no. 5: 3123–3132. 10.1016/j.clnu.2020.12.012. 33358231

[fsn371243-bib-0032] Mbuagbaw, L. , B. Rochwerg , R. Jaeschke , et al. 2017. “Approaches to Interpreting and Choosing the Best Treatments in Network Meta‐Analyses.” Systematic Reviews 6, no. 1: 79. 10.1186/s13643-017-0473-z. 28403893 PMC5389085

[fsn371243-bib-0033] Moore, D. R. , D. M. Camera , J. L. Areta , and J. A. Hawley . 2014. “Beyond Muscle Hypertrophy: Why Dietary Protein Is Important for Endurance Athletes.” Applied Physiology, Nutrition and Metabolism 39, no. 9: 987–997. 10.1139/apnm-2013-0591. 24806440

[fsn371243-bib-0034] Mph, P. E. 2024. “Does Beta‐Alanine Improve Exercise Performance ?” Training121. https://www.training121.com/post/does‐beta‐alanine‐improve‐exercise‐performance.

[fsn371243-bib-0035] Nikolakopoulou, A. , J. P. T. Higgins , T. Papakonstantinou , et al. 2020. “CINeMA: An Approach for Assessing Confidence in the Results of a Network Meta‐Analysis.” PLoS Medicine 17, no. 4: e1003082. 10.1371/journal.pmed.1003082. 32243458 PMC7122720

[fsn371243-bib-0036] Papakonstantinou, T. , A. Nikolakopoulou , J. P. T. Higgins , M. Egger , and G. Salanti . 2020. “CINeMA: Software for Semiautomated Assessment of the Confidence in the Results of Network Meta‐Analysis.” Campbell Systematic Reviews 16, no. 1: e1080. 10.1002/cl2.1080. 37131978 PMC8356302

[fsn371243-bib-0037] Perez‐Schindler, J. , D. L. Hamilton , D. R. Moore , K. Baar , and A. Philp . 2015. “Nutritional Strategies to Support Concurrent Training.” European Journal of Sport Science 15: 41–52. 10.1080/17461391.2014.950345. 25159707

[fsn371243-bib-0038] Rathmacher, J. A. , L. M. Pitchford , J. R. Stout , et al. 2025. “International Society of Sports Nutrition Position Stand: β‐Hydroxy‐β‐Methylbutyrate (HMB).” Journal of the International Society of Sports Nutrition 22, no. 1: 2434734. 10.1080/15502783.2024.2434734. 39699070 PMC11740297

[fsn371243-bib-0039] Requena, B. , M. Zabala , P. Padial , and B. Feriche . 2005. “Sodium Bicarbonate and Sodium Citrate: Ergogenic Aids?” Journal of Strength & Conditioning Research 19, no. 1: 213. 15705037 10.1519/13733.1

[fsn371243-bib-0040] Rothschild, J. A. , and D. J. Bishop . 2020. “Effects of Dietary Supplements on Adaptations to Endurance Training.” Sports Medicine 50, no. 1: 25–53. 10.1007/s40279-019-01185-8. 31531769

[fsn371243-bib-0053] Sandford, G. N. , P. B. Laursen , and M. Buchheit . 2021. “Anaerobic Speed/Power Reserve and Sport Performance: Scientific Basis, Current Applications and Future Directions.” Sports Medicine 51, no. 10: 2017–2028. 10.1007/s40279-021-01523-9. 34398445

[fsn371243-bib-0041] Stancliffe, R. A. 2012. “Role of Beta‐Hydroxy‐Beta‐Methylbutyrate (HMB) in Leucine Stimulation of Mitochondrial Biogenesis and Fatty Acid Oxidation.” Masters Theses. https://trace.tennessee.edu/utk_gradthes/1398.

[fsn371243-bib-0042] van Loon, L. J. C. , A. M. Oosterlaar , F. Hartgens , M. K. C. Hesselink , R. J. Snow , and A. J. M. Wagenmakers . 2003. “Effects of Creatine Loading and Prolonged Creatine Supplementation on Body Composition, Fuel Selection, Sprint and Endurance Performance in Humans.” Clinical Science 104, no. 2: 153–162. 10.1042/cs1040153. 12546637

[fsn371243-bib-0043] Veroniki, A. A. , S. E. Straus , A. Fyraridis , and A. C. Tricco . 2016. “The Rank‐Heat Plot Is a Novel Way to Present the Results From a Network Meta‐Analysis Including Multiple Outcomes.” Journal of Clinical Epidemiology 76: 193–199. 10.1016/j.jclinepi.2016.02.016. 26939929

[fsn371243-bib-0054] Viribay, A. , J. Burgos , J. Fernández‐Landa , J. Seco‐Calvo , and J. Mielgo‐Ayuso . 2020. “Effects of Arginine Supplementation on Athletic Performance Based on Energy Metabolism: A Systematic Review and Meta‐Analysis.” Nutrients 12, no. 5: 1300. 10.3390/nu12051300. 32370176 PMC7282262

[fsn371243-bib-0044] Vukovich, M. D. , and G. D. Dreifort . 2001. “Effect of β‐Hydroxy β‐Methylbutyrate on the Onset of Blood Lactate Accumulation and Vo2peak in Endurance‐Trained Cyclists.” Journal of Strength & Conditioning Research 15, no. 4: 491–497. 11726262

[fsn371243-bib-0045] Welton, N. J. , A. J. Sutton , N. Cooper , K. R. Abrams , and A. E. Ades . 2012. Evidence Synthesis for Decision Making in Healthcare. John Wiley & Sons.

[fsn371243-bib-0046] Wickham, K. A. , and L. L. Spriet . 2019. “No Longer Beeting Around the Bush: A Review of Potential Sex Differences With Dietary Nitrate Supplementation.” Applied Physiology, Nutrition and Metabolism 44, no. 9: 915–924. 10.1139/apnm-2019-0063. 31348674

[fsn371243-bib-0047] Wilson, G. J. , J. M. Wilson , and A. H. Manninen . 2008. “Effects of Beta‐Hydroxy‐Beta‐Methylbutyrate (HMB) on Exercise Performance and Body Composition Across Varying Levels of Age, Sex, and Training Experience: A Review.” Nutrition & Metabolism 5: 1. 10.1186/1743-7075-5-1. 18173841 PMC2245953

[fsn371243-bib-0048] Wyss, M. , and R. Kaddurah‐Daouk . 2000. “Creatine and Creatinine Metabolism.” Physiological Reviews 80, no. 3: 1107–1213. 10.1152/physrev.2000.80.3.1107. 10893433

[fsn371243-bib-0049] Zadik, Z. , D. Nemet , and A. Eliakim . 2009. “Hormonal and Metabolic Effects of Nutrition in Athletes.” Journal of Pediatric Endocrinology and Metabolism 22, no. 9: 769–778. 10.1515/JPEM.2009.22.9.769. 19960886

[fsn371243-bib-0050] Zart, S. , and M. Fröhlich . 2025. “Ergogenic Effects of Supplement Combinations on Endurance Performance: A Systematic Review and Meta‐Analysis of Randomized Controlled Trials.” Journal of the International Society of Sports Nutrition 22, no. 1: 2524033. 10.1080/15502783.2025.2524033. 40619880 PMC12239112

