# Supplementary material for: Dietary Supplement Strategies During Conditioning Training in Athletes: A Network Meta‐Analysis of Peak and Mean Anaerobic Power, VO 2max, and Endurance Performance
Source: Food Sci Nutr. 2025 Nov 29;13(12):e71243. doi: 10.1002/fsn3.71243 (PMC12663695; doi:10.1002/fsn3.71243)

Supplementary Appendix

# Dietary Supplement Strategies during Conditioning Training in Athletes: A Network Meta-analysis of Peak and Mean Anaerobic Power, VO₂max, and Endurance Performance

**Table of contents**

[List of Abbreviations 2](#_Toc20238)

[Appendix 1: Search strategy 3](#_Toc17788)

[Appendix 2: Characteristics of included studies 13](#_Toc1311)

[Appendix 3: Risk of bias of randomized clinical trials 16](#_Toc3484)

[Appendix 4: Evaluation of inconsistency and heterogeneity 18](#_Toc19816)

[Appendix 5: Network maps and forest plots of secondary outcomes 20](#_Toc13297)

[Appendix 6: SUCRA and cumulative probability plots 24](#_Toc14716)

[Appendix 7: League Table of Summary Estimates for Dietary Supplementation Combined with Strength and Conditioning on Athletic Performance from on Network Meta-Analysis 26](#_Toc15513)

[Appendix 8: CINeMA Assessment 28](#_Toc31351)

[Figure S8.3: Risk of bias contribution by intervention group in jumping performance 30](#_Toc9195)

[Appendix 9: Funnel plots 38](#_Toc5208)

# List of Abbreviations

The following abbreviations are used in this manuscript:

| **Abbreviation** | **Full Term** |
| --- | --- |
| HMB | β-hydroxy-β-methylbutyrate |
| S&C | strength and conditioning |
| CINeMA | Confidence in Network Meta-Analysis |
| SUCRA | surface under the cumulative ranking curve |
| PL | placebo |
| PR | protein |
| CR | creatine |
| BA | β-alanine |
| VD | Vitamin D3 |
| nitrate | NO3 |
| RCTs | randomised controlled trials |
| VO₂max | maximal oxygen uptake |

# Appendix 1: Search strategy

**Table S1.** Search strategy of Pubmed

| **#** | **Searches** |
| --- | --- |
| 1 | ((((((((((((((((((((((((((((((((((((((((((((((((((Athlete[MeSH Terms]) OR (athlete[Title/Abstract])) OR (Professional Athletes[Title/Abstract])) OR (Athlete, Professional[Title/Abstract])) OR (Athletes, Professional[Title/Abstract])) OR (Professional Athlete[Title/Abstract])) OR (Elite Athletes[Title/Abstract])) OR (Athlete, Elite[Title/Abstract])) OR (Athletes, Elite[Title/Abstract])) OR (Elite Athlete[Title/Abstract])) OR (College Athletes[Title/Abstract])) OR (Athlete, College[Title/Abstract])) OR (Athletes, College[Title/Abstract])) OR (College Athlete[Title/Abstract])) OR (sports playe[Title/Abstract])) OR (Sportsperson[Title/Abstract])) OR (trained individuals[Title/Abstract])) OR (Sports[Title/Abstract])) OR (Football[Title/Abstract])) OR (Soccer[Title/Abstract])) OR (hockey[Title/Abstract])) OR (basketball[Title/Abstract])) OR (Netball[Title/Abstract])) OR (Volleyball[Title/Abstract])) OR (track and field[Title/Abstract])) OR (Cycli[Title/Abstract])) OR (running[Title/Abstract])) OR (Runner[Title/Abstract])) OR (Swim[Title/Abstract])) OR (Handball[Title/Abstract])) OR (Softball[Title/Abstract])) OR (Tennis[Title/Abstract])) OR (baseball[Title/Abstract])) OR (cross country[Title/Abstract])) OR (cricket[Title/Abstract])) OR (Surf[Title/Abstract])) OR (Skiing[Title/Abstract])) OR (Golf[Title/Abstract])) OR (Hurdling[Title/Abstract])) OR (Bicycling[Title/Abstract])) OR (Boxing[Title/Abstract])) OR (Gymnast[Title/Abstract])) OR (martial arts[Title/Abstract])) OR (racquet sports[Title/Abstract])) OR (Badminton[Title/Abstract])) OR (Jogg[Title/Abstract])) OR (Walk[Title/Abstract])) OR (weight lifting[Title/Abstract])) OR (Lift[Title/Abstract])) OR (Weights[Title/Abstract])) OR (wrestling[Title/Abstract]) |
| 2 | ((((((((((((((((((((((((((((((((((((((((((((((((((((Dietary Supplement[MeSH Terms]) OR (nutrition intervention,[Title/Abstract])) OR (nutrition supplement[Title/Abstract])) OR (nutrition support[Title/Abstract])) OR (nutrition therapy[Title/Abstract])) OR (nutrition method[Title/Abstract])) OR (nutrition technique[Title/Abstract])) OR (nutrition treatment[Title/Abstract])) OR (nutrition modification[Title/Abstract])) OR (multinutrient supplement[Title/Abstract])) OR (multinutrient intervention[Title/Abstract])) OR (nutritional supplementation[Title/Abstract])) OR (Supplements, Dietary[Title/Abstract])) OR (Dietary Supplementations[Title/Abstract])) OR (Supplementations, Dietary[Title/Abstract])) OR (Nutrition Therapy[Title/Abstract])) OR (Food Supplementations[Title/Abstract])) OR (Food Supplements[Title/Abstract])) OR (Food Supplement[Title/Abstract])) OR (Supplement, Food[Title/Abstract])) OR (Supplements, Food[Title/Abstract])) OR (Nutraceuticals[Title/Abstract])) OR (Nutraceutical[Title/Abstract])) OR (Nutriceuticals[Title/Abstract])) OR (Nutriceutical[Title/Abstract])) OR (Neutraceuticals[Title/Abstract])) OR (Neutraceutical[Title/Abstract])) OR (Herbal Supplements[Title/Abstract])) OR (Herbal Supplement[Title/Abstract])) OR (Supplement, Herbal[Title/Abstract])) OR (Supplements, Herbal[Title/Abstract])) OR (nutritional intake[Title/Abstract])) OR (protein supplement[Title/Abstract])) OR (Creatine[Title/Abstract])) OR (beta Alanine[Title/Abstract])) OR (β-alanine[Title/Abstract])) OR (Leucine[Title/Abstract])) OR (amino acid[Title/Abstract])) OR (carbohydrate loading[Title/Abstract])) OR (branched chain amino acid[Title/Abstract])) OR (proteins[Title/Abstract])) OR (protein[Title/Abstract])) OR (amino acids[Title/Abstract])) OR (amino[Title/Abstract])) OR (acids[Title/Abstract])) OR (leucine[Title/Abstract])) OR (whey proteins[Title/Abstract])) OR (whey protein[Title/Abstract])) OR (whey[Title/Abstract])) OR (vitamin[Title/Abstract])) OR (beetroot juice[Title/Abstract])) OR (caffeine[Title/Abstract])) OR (HMB[Title/Abstract]) |
| 3 | ((((((((((((((((((((((((((((((((Physical Conditioning, Human[MeSH Terms]) OR (Fitness Training[Title/Abstract])) OR (Athletic Conditioning[Title/Abstract])) OR (Strength AND Conditioning[Title/Abstract])) OR (Sport Training[Title/Abstract])) OR (Motor Control Training[Title/Abstract])) OR (Functional Training[Title/Abstract])) OR (Functional Training[Title/Abstract])) OR (Resistance Training[Title/Abstract])) OR (Resistance Training[Title/Abstract])) OR (Resistance Training[Title/Abstract])) OR (Resistance Training[Title/Abstract])) OR (Training, Resistance[Title/Abstract])) OR (Weight Training[Title/Abstract])) OR (Weight-Lifting Strengthening Program[Title/Abstract])) OR (Strengthening Program, Weight-Lifting[Title/Abstract])) OR (Weight Lifting Strengthening Program[Title/Abstract])) OR (Weight-Lifting Strengthening Programs[Title/Abstract])) OR (Weight-Lifting Exercise Program[Title/Abstract])) OR (Exercise Programs, Weight-Lifting[Title/Abstract])) OR (Exercise Program, Weight-Lifting[Title/Abstract])) OR (Weight Lifting Exercise Program[Title/Abstract])) OR (Weight-Lifting Exercise Programs[Title/Abstract])) OR (Weight-Bearing Strengthening Program[Title/Abstract])) OR (Strengthening Programs, Weight-Bearing[Title/Abstract])) OR (Strengthening Program, Weight-Bearing[Title/Abstract])) OR (Weight Bearing Strengthening Program[Title/Abstract])) OR (Weight-Bearing Strengthening Programs[Title/Abstract])) OR (Weight-Bearing Exercise Program[Title/Abstract])) OR (Exercise Programs, Weight-Bearing[Title/Abstract])) OR (Exercise Program, Weight-Bearing[Title/Abstract])) OR (Weight Bearing Exercise Program[Title/Abstract])) OR (Weight-Bearing Exercise Programs[Title/Abstract]) |
| 4 | ((((((((((((((((((((((((((((((((((((((Plyometric Training[Title/Abstract]) OR (Plyometric Exercises[Title/Abstract])) OR (Plyometric Drills[Title/Abstract])) OR (Exercise, Plyometric[Title/Abstract])) OR (Exercises, Plyometric[Title/Abstract])) OR (Training, Plyometric[Title/Abstract])) OR (Trainings, Plyometric[Title/Abstract])) OR (Stretch-Shortening Cycle[Title/Abstract])) OR (Stretch-Shortening Exercises[Title/Abstract])) OR (Stretch-Shortening Drills[Title/Abstract])) OR (Stretch-Shortening Exercise[Title/Abstract])) OR (Stretch-Shortening Cycle Exercise[Title/Abstract])) OR (Stretch-Shortening Cycle Exercise[Title/Abstract])) OR (Stretch Shortening Exercise[Title/Abstract])) OR (Stretch Shortening Drill[Title/Abstract])) OR (Drill, Plyometric[Title/Abstract])) OR (Drills, Plyometric[Title/Abstract])) OR (Drill, Stretch-Shortening[Title/Abstract])) OR (Drills, Stretch-Shortening[Title/Abstract])) OR (Endurance Training[Title/Abstract])) OR (Aerobic Training[Title/Abstract])) OR (Sprint Interval Training[Title/Abstract])) OR (Sprint Training[Title/Abstract])) OR (Sprint Training[Title/Abstract])) OR (Agility Training[Title/Abstract])) OR (High-Intensity Interval Training[Title/Abstract])) OR (HIIT[Title/Abstract])) OR (Interval Training, High-Intensity[Title/Abstract])) OR (Interval Trainings, High-Intensity[Title/Abstract])) OR (Training, High-Intensity Interval[Title/Abstract])) OR (Training, High-Intensity Interval[Title/Abstract])) OR (Training, High-Intensity Interval[Title/Abstract])) OR (Exercise, High-Intensity Intermittent[Title/Abstract])) OR (Exercises, High-Intensity Intermittent[Title/Abstract])) OR (High-Intensity Intermittent Exercises[Title/Abstract])) OR (Anaerobic Training[Title/Abstract])) OR (Anaerobic Exercise[Title/Abstract])) OR (Anaerobic Capacity[Title/Abstract])) OR (Sprint Exercise[Title/Abstract]) |
| 5 | ((((((randomized controlled trial[Publication Type]) OR (randomized)) OR (clinical trials)) OR (placebo)) OR (randomly)) OR (trial)) OR (RCT) |
| 6 | #3 OR #4 |
| 7 | #1 AND #2 AND #5 AND #6 |

**Table S2.** Search strategy of Web of Science

| **#** | **Searches** |
| --- | --- |
| 1 | TS=("Athlete" OR "athlete" OR "Professional Athletes" OR "Athlete, Professional" OR "Athletes, Professional" OR "Professional Athlete" OR "Elite Athletes" OR "Athlete, Elite" OR "Athletes, Elite" OR "Athletes, Elite" OR "College Athletes" OR "College Athletes" OR "Athlete, College" OR "Athletes, College" OR "College Athlete" OR "sports playe" OR "Sportsperson" OR "trained individuals" OR "Sports" OR "Football" OR "Soccer" OR "hockey" OR "basketball" OR "Netball" OR "Volleyball" OR "track and field" OR "Cycli" OR "running" OR "Runner" OR "Swim" OR "Handball" OR "Softball" OR "Tennis" OR "baseball" OR "cross country" OR "cricket" OR "Surf" OR "Skiing" OR "Golf" OR "Hurdling" OR "Bicycling" OR "Boxing" OR "Gymnast" OR "martial arts" OR "racquet sports" OR "Badminton" OR "Jogg" OR "Walk" OR "weight lifting" OR "Lift" OR "Weights" OR "wrestling") |
| 2 | TS=("Dietary Supplement" OR "nutrition intervention" OR "nutrition supplement" OR "nutrition support" OR "nutrition therapy" OR "nutrition method" OR "nutrition technique" OR "nutrition treatment" OR "nutrition modification" OR "multinutrient supplement" OR "multinutrient intervention" OR "nutritional supplementation" OR "Supplements, Dietary" OR "Dietary Supplementations" OR "Supplementations, Dietary" OR "Nutrition Therapy" OR "Food Supplementations" OR "Food Supplements" OR "Food Supplement" OR "Supplement, Food" OR "Supplements, Food" OR "Nutraceuticals" OR "Nutraceutical" OR "Nutriceuticals" OR "Nutriceutical" OR "Neutraceuticals" OR "Neutraceutical" OR "Herbal Supplements" OR "Herbal Supplement" OR "Supplement, Herbal" OR "Supplements, Herbal" OR "nutritional intake" OR "protein supplement" OR "Creatine" OR "beta Alanine" OR "β-alanine" OR "Leucine" OR "amino acid" OR "carbohydrate loading" OR "branched chain amino acid" OR "proteins" OR "protein" OR "amino acids" OR "amino" OR "acids" OR "leucine" OR "whey proteins" OR "whey protein" OR "whey" OR "vitamin" OR "beetroot juice" OR "caffeine" OR "HMB") |
| 3 | TS=("Physical Conditioning, Human" OR "Fitness Training" OR "Athletic Conditioning" OR "Strength AND Conditioning" OR "Sport Training" OR "Motor Control Training" OR "Functional Training" OR "Resistance Training" OR "Training, Resistance" OR "Weight Training" OR "Weight-Lifting Strengthening Program" OR "Strengthening Program, Weight-Lifting" OR "Weight Lifting Strengthening Program" OR "Weight-Lifting Strengthening Programs" OR "Weight-Lifting Exercise Program" OR "Exercise Programs, Weight-Lifting" OR "Exercise Program, Weight-Lifting" OR "Weight Lifting Exercise Program" OR "Weight-Lifting Exercise Programs" OR "Weight-Bearing Strengthening Program" OR "Strengthening Programs, Weight-Bearing" OR "Strengthening Program, Weight-Bearing" OR "Weight Bearing Strengthening Program" OR "Weight-Bearing Strengthening Programs" OR "Weight-Bearing Exercise Program" OR "Exercise Programs, Weight-Bearing" OR "Exercise Program, Weight-Bearing" OR "Weight Bearing Exercise Program" OR "Weight-Bearing Exercise Programs") |
| 4 | TS=("Plyometric Training" OR "Plyometric Exercises" OR "Plyometric Drills" OR "Exercise, Plyometric" OR "Exercises, Plyometric" OR "Training, Plyometric" OR "Trainings, Plyometric" OR "Stretch-Shortening Cycle" OR "Stretch-Shortening Exercises" OR "Stretch-Shortening Drills" OR "Stretch-Shortening Exercise" OR "Stretch-Shortening Cycle Exercise" OR "Stretch Shortening Exercise" OR "Stretch Shortening Drill" OR "Drill, Plyometric" OR "Drills, Plyometric" OR "Drill, Stretch-Shortening" OR "Drills, Stretch-Shortening" OR "Endurance Training" OR "Aerobic Training" OR "Sprint Interval Training" OR "Sprint Training" OR "Agility Training" OR "High-Intensity Interval Training" OR "HIIT" OR "Interval Training, High-Intensity" OR "Interval Trainings, High-Intensity" OR "Training, High-Intensity Interval" OR "Exercise, High-Intensity Intermittent" OR "Exercises, High-Intensity Intermittent" OR "High-Intensity Intermittent Exercises" OR "Anaerobic Training" OR "Anaerobic Exercise" OR "Anaerobic Capacity" OR "Sprint Exercise") |
| 5 | ALL=("randomized controlled trial" OR "randomized" OR "clinical trials" OR "placebo" OR "randomly" OR "trial" OR "RCT") |
| 6 | #3 AND #4 |
| 7 | #1 AND #2 AND #5 AND #6 |

**Table S3.** Search strategy of Embase

| **#** | **Searches** |
| --- | --- |
| 1 | 'athlete'/exp |
| 2 | athlete:ti,ab,kw OR 'professional athlete':ti,ab,kw OR 'elite athlete':ti,ab,kw OR 'collegiate athlete':ti,ab,kw OR 'sports player':ti,ab,kw OR 'sportsperson':ti,ab,kw OR 'trained individuals':ti,ab,kw OR 'resistance-trained':ti,ab,kw OR 'strength-trained':ti,ab,kw OR 'power-trained':ti,ab,kw OR 'weightlifter':ti,ab,kw OR 'powerlifter':ti,ab,kw OR 'strength athlete':ti,ab,kw OR 'bodybuilder':ti,ab,kw OR 'endurance athlete':ti,ab,kw OR 'team-sport athlete':ti,ab,kw OR 'high-performance athlete':ti,ab,kw OR 'physically trained individuals':ti,ab,kw OR 'competitive athlete':ti,ab,kw OR 'football':ti,ab,kw OR 'soccer':ti,ab,kw OR 'hockey':ti,ab,kw OR 'basketball':ti,ab,kw OR 'netball':ti,ab,kw OR 'volleyball':ti,ab,kw OR 'track and field':ti,ab,kw OR 'cycling':ti,ab,kw OR 'running':ti,ab,kw OR 'runner':ti,ab,kw OR 'swimming':ti,ab,kw OR 'swimmer':ti,ab,kw OR 'handball':ti,ab,kw OR 'softball':ti,ab,kw OR 'tennis':ti,ab,kw OR 'baseball':ti,ab,kw OR 'cross country':ti,ab,kw OR 'cricket':ti,ab,kw OR 'surfing':ti,ab,kw OR 'skiing':ti,ab,kw OR 'golf':ti,ab,kw OR 'hurdling':ti,ab,kw OR 'bicycling':ti,ab,kw OR 'boxing':ti,ab,kw OR 'gymnastics':ti,ab,kw OR 'martial arts':ti,ab,kw OR 'racquet sports':ti,ab,kw OR 'badminton':ti,ab,kw OR 'jogging':ti,ab,kw OR 'walking':ti,ab,kw OR 'weight lifting':ti,ab,kw OR 'weight training':ti,ab,kw OR 'wrestling':ti,ab,kw |
| 3 | 'dietary supplement'/exp |
| 4 | 'nutrition intervention':ti,ab,kw OR 'nutrition supplement':ti,ab,kw OR 'nutrition support':ti,ab,kw OR 'nutrition method':ti,ab,kw OR 'nutrition technique':ti,ab,kw OR 'nutrition treatment':ti,ab,kw OR 'nutrition modification':ti,ab,kw OR 'multinutrient supplement':ti,ab,kw OR 'multinutrient intervention':ti,ab,kw OR 'nutritional supplementation':ti,ab,kw OR 'supplements, dietary':ti,ab,kw OR 'dietary supplementations':ti,ab,kw OR 'supplementations, dietary':ti,ab,kw OR 'nutrition therapy':ti,ab,kw OR 'food supplementations':ti,ab,kw OR 'food supplements':ti,ab,kw OR 'food supplement':ti,ab,kw OR 'supplement, food':ti,ab,kw OR 'supplements, food':ti,ab,kw OR 'nutraceuticals':ti,ab,kw OR 'nutraceutical':ti,ab,kw OR 'nutriceuticals':ti,ab,kw OR 'nutriceutical':ti,ab,kw OR 'neutraceuticals':ti,ab,kw OR 'neutraceutical':ti,ab,kw OR 'herbal supplements':ti,ab,kw OR 'herbal supplement':ti,ab,kw OR 'supplement, herbal':ti,ab,kw OR 'supplements, herbal':ti,ab,kw OR 'nutritional intake':ti,ab,kw OR 'protein supplement':ti,ab,kw OR 'creatine':ti,ab,kw OR 'beta alanine':ti,ab,kw OR 'β-alanine':ti,ab,kw OR 'leucine':ti,ab,kw OR 'amino acid':ti,ab,kw OR 'carbohydrate loading':ti,ab,kw OR 'branched chain amino acid':ti,ab,kw OR 'proteins':ti,ab,kw OR 'protein':ti,ab,kw OR 'amino acids':ti,ab,kw OR 'amino':ti,ab,kw OR 'acids':ti,ab,kw OR 'whey proteins':ti,ab,kw OR 'whey protein':ti,ab,kw OR 'whey':ti,ab,kw OR 'vitamin':ti,ab,kw OR 'beetroot juice':ti,ab,kw OR 'caffeine':ti,ab,kw OR 'hmb':ti,ab,kw |
| 5 | 'physical conditioning, human'/exp |
| 6 | 'physical conditioning, human':ti,ab,kw OR 'fitness training':ti,ab,kw OR 'athletic conditioning':ti,ab,kw OR ('strength':ti,ab,kw AND 'conditioning':ti,ab,kw) OR 'sport training':ti,ab,kw OR 'motor control training':ti,ab,kw OR 'functional training':ti,ab,kw OR 'resistance training':ti,ab,kw OR 'training, resistance':ti,ab,kw OR 'weight training':ti,ab,kw OR 'weight-lifting strengthening program':ti,ab,kw OR 'strengthening program, weight-lifting':ti,ab,kw OR 'weight lifting strengthening program':ti,ab,kw OR 'weight-lifting strengthening programs':ti,ab,kw OR 'weight-lifting exercise program':ti,ab,kw OR 'exercise programs, weight-lifting':ti,ab,kw OR 'exercise program, weight-lifting':ti,ab,kw OR 'weight lifting exercise program':ti,ab,kw OR 'weight-lifting exercise programs':ti,ab,kw OR 'weight-bearing strengthening program':ti,ab,kw OR 'strengthening programs, weight-bearing':ti,ab,kw OR 'strengthening program, weight-bearing':ti,ab,kw OR 'weight bearing strengthening program':ti,ab,kw OR 'weight-bearing strengthening programs':ti,ab,kw OR 'weight-bearing exercise program':ti,ab,kw OR 'exercise programs, weight-bearing':ti,ab,kw OR 'exercise program, weight-bearing':ti,ab,kw OR 'weight bearing exercise program':ti,ab,kw OR 'weight-bearing exercise programs':ti,ab,kw |
| 7 | 'plyometric training':ti,ab,kw OR 'plyometric exercises':ti,ab,kw OR 'plyometric drills':ti,ab,kw OR 'exercise, plyometric':ti,ab,kw OR 'exercises, plyometric':ti,ab,kw OR 'training, plyometric':ti,ab,kw OR 'trainings, plyometric':ti,ab,kw OR 'stretch-shortening cycle':ti,ab,kw OR 'stretch-shortening exercises':ti,ab,kw OR 'stretch-shortening drills':ti,ab,kw OR 'stretch-shortening exercise':ti,ab,kw OR 'stretch-shortening cycle exercise':ti,ab,kw OR 'stretch shortening exercise':ti,ab,kw OR 'stretch shortening drill':ti,ab,kw OR 'drill, plyometric':ti,ab,kw OR 'drills, plyometric':ti,ab,kw OR 'drill, stretch-shortening':ti,ab,kw OR 'drills, stretch-shortening':ti,ab,kw OR 'endurance training':ti,ab,kw OR 'aerobic training':ti,ab,kw OR 'sprint interval training':ti,ab,kw OR 'sprint training':ti,ab,kw OR 'agility training':ti,ab,kw OR 'high-intensity interval training':ti,ab,kw OR 'hiit':ti,ab,kw OR 'interval training, high-intensity':ti,ab,kw OR 'interval trainings, high-intensity':ti,ab,kw OR 'training, high-intensity interval':ti,ab,kw OR 'exercise, high-intensity intermittent':ti,ab,kw OR 'exercises, high-intensity intermittent':ti,ab,kw OR 'high-intensity intermittent exercises':ti,ab,kw OR 'anaerobic training':ti,ab,kw OR 'anaerobic exercise':ti,ab,kw OR 'anaerobic capacity':ti,ab,kw OR 'sprint exercise':ti,ab,kw |
| 8 | 'randomized controlled trial'/exp |
| 9 | 'randomized controlled trial':ti,ab,kw OR 'randomized':ti,ab,kw OR 'clinical trials':ti,ab,kw OR 'placebo':ti,ab,kw OR 'randomly':ti,ab,kw OR 'trial':ti,ab,kw OR 'rct':ti,ab,kw |
| 10 | #1 OR #2 |
| 11 | #3 OR #4 |
| 12 | #5 OR #6 OR #7 |
| 13 | #8 OR #9 |
| 14 | #10 AND #11 AND #12 AND #13 |

**Table S4.** Search strategy of SPORTDiscus

| **#** | **Searches** |
| --- | --- |
| 1 | SU "Athlete" OR "athlete" OR "Professional Athletes" OR "Athlete, Professional" OR "Athletes, Professional" OR "Professional Athlete" OR "Elite Athletes" OR "Athlete, Elite" OR "Athletes, Elite" OR "Athletes, Elite" OR "College Athletes" OR "College Athletes" OR "Athlete, College" OR "Athletes, College" OR "College Athlete" OR "sports playe" OR "Sportsperson" OR "trained individuals" OR "Sports" OR "Football" OR "Soccer" OR "hockey" OR "basketball" OR "Netball" OR "Volleyball" OR "track and field" OR "Cycli" OR "running" OR "Runner" OR "Swim" OR "Handball" OR "Softball" OR "Tennis" OR "baseball" OR "cross country" OR "cricket" OR "Surf" OR "Skiing" OR "Golf" OR "Hurdling" OR "Bicycling" OR "Boxing" OR "Gymnast" OR "martial arts" OR "racquet sports" OR "Badminton" OR "Jogg" OR "Walk" OR "weight lifting" OR "Lift" OR "Weights" OR "wrestling" |
| 2 | SU "Dietary Supplement" OR "nutrition intervention" OR "nutrition supplement" OR "nutrition support" OR "nutrition therapy" OR "nutrition method" OR "nutrition technique" OR "nutrition treatment" OR "nutrition modification" OR "multinutrient supplement" OR "multinutrient intervention" OR "nutritional supplementation" OR "Supplements, Dietary" OR "Dietary Supplementations" OR "Supplementations, Dietary" OR "Nutrition Therapy" OR "Food Supplementations" OR "Food Supplements" OR "Food Supplement" OR "Supplement, Food" OR "Supplements, Food" OR "Nutraceuticals" OR "Nutraceutical" OR "Nutriceuticals" OR "Nutriceutical" OR "Neutraceuticals" OR "Neutraceutical" OR "Herbal Supplements" OR "Herbal Supplement" OR "Supplement, Herbal" OR "Supplements, Herbal" OR "nutritional intake" OR "protein supplement" OR "Creatine" OR "beta Alanine" OR "β-alanine" OR "Leucine" OR "amino acid" OR "carbohydrate loading" OR "branched chain amino acid" OR "proteins" OR "protein" OR "amino acids" OR "amino" OR "acids" OR "leucine" OR "whey proteins" OR "whey protein" OR "whey" OR "vitamin" OR "beetroot juice" OR "caffeine" OR "HMB" |
| 3 | SU "Physical Conditioning, Human" OR "Fitness Training" OR "Athletic Conditioning" OR "Strength and Conditioning" OR "Sport Training" OR "Motor Control Training" OR "Functional Training" OR "Resistance Training" OR "Training, Resistance" OR "Weight Training" OR "Weight-Lifting Strengthening Program" OR "Strengthening Programs, Weight-Lifting" OR "Strengthening Program, Weight-Lifting" OR "Weight Lifting Strengthening Program" OR "Weight-Lifting Strengthening Programs" OR "Weight-Lifting Exercise Program" OR "Exercise Programs, Weight-Lifting" OR "Exercise Program, Weight-Lifting" OR "Weight Lifting Exercise Program" OR "Weight-Lifting Exercise Programs" OR "Weight-Bearing Strengthening Program" OR "Strengthening Programs, Weight-Bearing" OR "Strengthening Program, Weight-Bearing" OR "Weight Bearing Strengthening Program" OR "Weight-Bearing Strengthening Programs" OR "Weight-Bearing Exercise Program" OR "Exercise Programs, Weight-Bearing" OR "Exercise Program, Weight-Bearing" OR "Weight Bearing Exercise Program" OR "Weight-Bearing Exercise Programs" |
| 4 | "Plyometric Training" OR "Plyometric Exercises" OR "Plyometric Drills" OR "Exercise, Plyometric" OR "Exercises, Plyometric" OR "Training, Plyometric" OR "Trainings, Plyometric" OR "Stretch-Shortening Cycle" OR "Stretch-Shortening Exercises" OR "Stretch-Shortening Drills" OR "Stretch-Shortening Exercise" OR "Stretch-Shortening Cycle Exercise" OR "Stretch Shortening Exercise" OR "Stretch Shortening Drill" OR "Drill, Plyometric" OR "Drills, Plyometric" OR "Drill, Stretch-Shortening" OR "Drills, Stretch-Shortening" OR "Endurance Training" OR "Aerobic Training" OR "Sprint Interval Training" OR "Sprint Training" OR "Agility Training" OR "High-Intensity Interval Training" OR "HIIT" OR "Interval Training, High-Intensity" OR "Interval Trainings, High-Intensity" OR "Exercise, High-Intensity Intermittent" OR "Exercises, High-Intensity Intermittent" OR "High-Intensity Intermittent Exercises" OR "Anaerobic Training" OR "Anaerobic Exercise" OR "Anaerobic Capacity" OR "Sprint Exercise" |
| 5 | TX "randomized controlled trial" OR "randomized" OR "clinical trials" OR "placebo" OR "randomly" OR "trial" OR "RCT" |
| 6 | #3 OR #4 |
| 7 | #1 AND #2 AND #5 AND #6 |

**Table S5.** Search strategy of Cochrane library

| **#** | **Searches** |
| --- | --- |
| 1 | MeSH descriptor: [Athletes] explode all trees |
| 2 | ('athlete' OR 'professional athlete' OR 'elite athlete' OR 'collegiate athlete' OR 'sports player' OR 'sportsperson' OR 'trained individuals' OR 'resistance-trained' OR 'strength-trained' OR 'power-trained' OR 'weightlifter' OR 'powerlifter' OR 'strength athlete' OR 'bodybuilder' OR 'endurance athlete' OR 'team-sport athlete' OR 'high-performance athlete' OR 'physically trained individuals' OR 'competitive athlete' OR 'football' OR 'soccer' OR 'hockey' OR 'basketball' OR 'netball' OR 'volleyball' OR 'track and field' OR 'cycling' OR 'running' OR 'runner' OR 'swimming' OR 'swimmer' OR 'handball' OR 'softball' OR 'tennis' OR 'baseball' OR 'cross country' OR 'cricket' OR 'surfing' OR 'skiing' OR 'golf' OR 'hurdling' OR 'bicycling' OR 'boxing' OR 'gymnastics' OR 'martial arts' OR 'racquet sports' OR 'badminton' OR 'jogging' OR 'walking' OR 'weight lifting' OR 'weight training' OR 'wrestling'):ti,ab,kw |
| 3 | MeSH descriptor: [Dietary Supplements] explode all trees |
| 4 | ('dietary supplement' OR 'nutrition supplement' OR 'nutrition support' OR 'nutrition method' OR 'nutrition technique' OR 'nutrition treatment' OR 'nutrition modification' OR 'multinutrient supplement' OR 'multinutrient intervention' OR 'nutrition intervention' OR 'supplements, dietary' OR 'dietary supplementations' OR 'supplementations, dietary' OR 'nutrition therapy' OR 'food supplementations' OR 'food supplements' OR 'food supplement' OR 'supplement, food' OR 'supplements, food' OR 'nutraceuticals' OR 'nutraceutical' OR 'nutriceuticals' OR 'nutriceutical' OR 'neutraceuticals' OR 'neutraceutical' OR 'herbal supplements' OR 'herbal supplement' OR 'supplement, herbal' OR 'supplements, herbal' OR 'nutritional intake' OR 'protein supplement' OR 'creatine' OR 'beta alanine' OR 'β-alanine' OR 'leucine' OR 'amino acid' OR 'carbohydrate loading' OR 'branched chain amino acid' OR 'proteins' OR 'protein' OR 'amino acids' OR 'amino' OR 'acids' OR 'whey proteins' OR 'whey protein' OR 'whey' OR 'vitamin' OR 'beetroot juice' OR 'caffeine' OR 'hmb'):ti,ab,kw |
| 5 | MeSH descriptor: [Physical Conditioning, Human] explode all trees |
| 6 | ('Physical Conditioning, Human' OR 'Fitness Training' OR 'Athletic Conditioning' OR 'Strength AND Conditioning' OR 'Sport Training' OR 'Motor Control Training' OR 'Functional Training' OR 'Resistance Training' OR 'Training, Resistance' OR 'Weight Training' OR 'Weight-Lifting Strengthening Program' OR 'Strengthening Program, Weight-Lifting' OR 'Weight Lifting Strengthening Program' OR 'Weight-Lifting Strengthening Programs' OR 'Weight-Lifting Exercise Program' OR 'Exercise Programs, Weight-Lifting' OR 'Exercise Program, Weight-Lifting' OR 'Weight Lifting Exercise Program' OR 'Weight-Lifting Exercise Programs' OR 'Weight-Bearing Strengthening Program' OR 'Strengthening Programs, Weight-Bearing' OR 'Strengthening Program, Weight-Bearing' OR 'Weight Bearing Strengthening Program' OR 'Weight-Bearing Strengthening Programs' OR 'Weight-Bearing Exercise Program' OR 'Exercise Programs, Weight-Bearing' OR 'Exercise Program, Weight-Bearing' OR 'Weight Bearing Exercise Program' OR 'Weight-Bearing Exercise Programs') |
| 7 | ('Plyometric Training' OR 'Plyometric Exercises' OR 'Plyometric Drills' OR 'Exercise, Plyometric' OR 'Exercises, Plyometric' OR 'Training, Plyometric' OR 'Trainings, Plyometric' OR 'Stretch-Shortening Cycle' OR 'Stretch-Shortening Exercises' OR 'Stretch-Shortening Drills' OR 'Stretch-Shortening Exercise' OR 'Stretch-Shortening Cycle Exercise' OR 'Stretch Shortening Exercise' OR 'Stretch Shortening Drill' OR 'Drill, Plyometric' OR 'Drills, Plyometric' OR 'Drill, Stretch-Shortening' OR 'Drills, Stretch-Shortening' OR 'Endurance Training' OR 'Aerobic Training' OR 'Sprint Interval Training' OR 'Sprint Training' OR 'Agility Training' OR 'High-Intensity Interval Training' OR 'HIIT' OR 'Interval Training, High-Intensity' OR 'Interval Trainings, High-Intensity' OR 'Training, High-Intensity Interval' OR 'Exercise, High-Intensity Intermittent' OR 'Exercises, High-Intensity Intermittent' OR 'High-Intensity Intermittent Exercises' OR 'Anaerobic Training' OR 'Anaerobic Exercise' OR 'Anaerobic Capacity' OR 'Sprint Exercise'):ti,ab,kw |
| 8 | MeSH descriptor: [Randomized Controlled Trial] explode all trees |
| 9 | ('randomized controlled trial' OR 'randomized' OR 'clinical trials' OR 'placebo' OR 'randomly' OR 'trial' OR 'rct'):ti,ab,kw |
| 10 | #1 OR #2 |
| 11 | #3 OR #4 |
| 12 | #5 OR #6 OR #7 |
| 13 | #9 OR #9 |
| 14 | #10 AND #11 AND #12 AND #13 |

# Appendix 2: Characteristics of included studies

**Table S2.1:** Baseline of characteristics of included studies

| Author  Year | Country | Group | Sample Size  (M/F) | Age  (Mean ± SD) | Exercise requency Frequency | dietary supplements | | Period | Results |
| --- | --- | --- | --- | --- | --- | --- | --- | --- | --- |
|  |  |  |  |  |  | categories | Exercise requency Frequency |  |  |
| Jones et al. 1999(Jones, Atter, & Georg, 1999) | Britain | RT | 8/0 | 27.0 ± 4 | 1-2/w | creatine | 20g/d for five days, 5g/d for the following days | 10W | peak power; Average power |
|  |  | c | 8/0 | 27.0 ± 4 | 1-2/w | glucose | Same dose |  |  |
| Brisola et al. 2016(Brisola, Artioli, Papoti, & Zagatto, 2016) | Brazil | RT | 11/0 | 19±5 | 2/d | β-alanine | First 10 days: 4.8 g/day, Last 18 days: 6.4 g/day | 4w | Endurance performance |
|  |  | C | 11/0 | 18±3 | 2/d | glucose | Same dose |  |  |
| Fairbairn et al. 2018a(Fairbairn, Ceelen, Skeaff, Cameron, & Perry, 2018) | New Zealand | RT | 28/0 | 21.5 |  | Vitamin D3 | 1.25mg，1/2w | 6w | Endurance performance |
|  |  | C | 29/0 | 20.9 |  | placebo | Same dose |  |  |
| Fairbairn et al. 2018b(Fairbairn et al., 2018) | New Zealand | RT | 28/0 | 21.5 |  | Vitamin D3 | 1.25mg，1/2w | 12w | Endurance performance |
|  |  | C | 29/0 | 20.9 |  | placebo | Same dose |  |  |
| Guo et al. 2024(Guo & Wang, 2024) | Korea | SSIT | 3/11 | 24.6 ± 2.5 | 3/w | β-alanine | 4.8g/d | 8w | maximal oxygen uptake; peak power; Average power |
|  |  | C | 4/8 | 23.8 ± 2.7 | 3/w | glucose | Same dose |  |  |
| Li et al.2005a(M. Li & Sheykhlouvand, 2025) | China | SSIT | 8/0 | 20.4 ± 1.2 | 3/w | creatine | 0.1 g·kg/d | 4w | maximal oxygen uptake; peak power; Average power |
|  |  | SSIT | 8/0 | 20.1 ± 1.6 | 3/w | β-alanine | 4.8 g/d |  |  |
|  |  | C | 8/0 | 20.5 ± 1.4 | 3/w | glucose | Same dose |  |  |
| Li et al.2005b(M. Li & Sheykhlouvand, 2025) | China | SSIT | 8/0 | 20.4 ± 1.2 | 3/w | creatine | 0.1 g·kg/d | 4w | maximal oxygen uptake; peak power; Average power |
|  |  | SSIT | 8/0 | 20.1 ± 1.6 | 3/w | β-alanine | 4.8 g/d |  |  |
|  |  | C | 8/0 | 20.5 ± 1.4 | 3/w | glucose | Same dose |  |  |
| Jastrzębska et al.2016(Jastrzębska, Kaczmarczyk, & Jastrzębski, 2016) | Poland | HIIT | 20/0 | 17.5 ± 0.6 | 4/w | Vitamin D | 5000 IU/d | 8w | peak power |
|  |  | C | 16/0 | 17.5 ± 0.6 | 4/w | placebo | Same dose |  |  |
| McIntosh et al. 2018(McIntosh, Love, Haszard, Osborne, & Black, 2018) | New Zealand | RT | 13/0 | 20.3 ± 1.2 | 4/w | HMB | 3g/d | 11w | Endurance performance |
|  |  | C | 14/0 | 21.9 ± 2.8 | 4/w | placebo | Same dose |  |  |
| Jung et al. 2018(Jung, Seo, Lee, Jung, & Song, 2018) | Korea | MMT | 12/18 | 20.1±0.15 | 5/w | Vitamin D₃ | 5000 IU/d | 4/w | peak power; Endurance performance |
|  |  | C | 9/6 | 20.1±0.15 | 5/w | placebo | Same dose |  |  |
| Bemben et al. 2001a(Bemben, Bemben, Loftiss, & Knehans, 2001) | America | MMT | 9/0 | 19.4±0.1 | 4/w | creatine | .1–5d：20 g/d,6d-9w：5 g/d | 9w | peak power; Average power |
|  |  | C | 8/0 | 19.3±0.5 | 4/w | glucose | Same dose |  |  |
| Bemben et al. 2001b(Bemben et al., 2001) | America | MMT | 9/0 | 19.4±0.1 | 4/w | creatine | .1–5d：20 g/d,6d-9w：5 g/d | 9w | peak power; Average power |
|  |  | C | 8/0 | 19.0±0.3 | 4/w | placebo | Same dose |  |  |
| Hoffman et al. 2007(J. R. Hoffman, Ratamess, Kang, Falvo, & Faigenbaum, 2007) | America | RT | 11/0 | 20.3±1.6 | 4/w | protein | 84/d | 10w | peak power; Average power |
|  |  | C | 10/0 | 21.0±1.2 | 4/w | maltodextrin | Same dose |  |  |
| Rosas et al. 2017(Rosas et al., 2017) | Argentina | PT | 0/8 | 24.3±2.5 | 2/w | β-alanine | 2.4g/d | 6w | Endurance performance |
|  |  | C | 0/8 | 22.8±2.1 | 2/w | placebo | Same dose |  |  |
| Ramírez-Campillo et al.2016(Ramírez-Campillo et al., 2016) | Chile | PT | 0/10 | 23.1±3.4 | 2/w | creatine | 1w:20g/d,2-6w:5g/d | 6w | Endurance performance |
|  |  | C | 0/10 | 22.9±1.7 | 2/w | glucose | Same dose |  |  |
| Hoffman et al. 2006(J. Hoffman et al., 2006) | America | RT | 11/0 | 21.5±2.3 | 4/w | creatine | 10.5 g/d | 10w | peak power;Average power |
|  |  | C | 11/0 | 21.5±2.3 | 4/w | glucose | Same dose |  |  |
| Murphy et al. 2005(Murphy, Watsford, Coutts, & Richards, 2005) | Australia | CSE | 9/0 | 24±3 |  | creatine | .1–7d：20 g/d,8d-4w：10 g/d | 4w | maximal oxygen uptake; Endurance performance |
|  |  | C | 9/0 | 24±3 |  | placebo | Same dose |  |  |
| Li et al. 2007(S.-C. Li & Zhao, 2007) | China | CSE | 8/0 | 22±1 | 6/w | protein | 50/d | 12w | peak power; Average power |
|  |  | C | 8/0 | 22±1 | 6/w | water |  |  |  |
| Mhamed et al. 20024(Mhamed et al., 2024) | Tunisia | CSE | 20/0 |  | 6/w | protein | 30/d | 8w | peak power; Average power |
|  |  | C | 9/0 |  | 6/w | placebo |  |  |  |
| Norberto et al. 2020(Norberto et al., 2020) | Brazil | RT | 13（6/7） | M: 20.25±1.98  W: 20.0± 2.92 | 2/w | β-alanine | 4.8 g/d | 6w | maximal oxygen uptake; Endurance performance |
|  |  | C |  |  | 2/w | starch | Same dose |  |  |
| Alabsi et al. 2023(Alabsi, Rashidlamir, & Dokht, 2023) | Iran | RT | 9/0 | 24.44±5.76 | 3/w | β-alanine | 0.3g/kg/d | 4w | peak power; Average power |
|  |  | C | 9/0 | 22.00±4.69 | 3/w | placebo | Same dose |  |  |
| Durkalec-Michalski et al(Durkalec-Michalski, Jeszka, & Podgórski, 2017) | Poland | MMT | 20/0 | 22.8±6.1 | 10/w | HMB | 3 g/d | 12w | maximal oxygen uptake; peak power; Average power |
|  |  | C | 20//0 | 22.8±6.1 | 10/w | placebo | Same dose |  |  |
| Portal et al. 2011(Portal et al., 2011) | Israel | MMT | 7/7 | 16.1±1.3 | 4-5/w | HMB | 3 g/d | 7w | maximal oxygen uptake; peak power; Average power |
|  |  | C | 7/7 | 16.2±1.3 | 4-5/w | placebo | Same dose |  |  |
| (Durkalec-Michalski et al. 2015(Durkalec-Michalski & Jeszka, 2015) | Poland | ET | 10/0 | 19.5 ± 1.4 | 5/w | HMB | 3 g/d | 12w | maximal oxygen uptake; peak power; Average power; Endurance performance |
|  |  | C | 10/0 | 19.5 ± 1.4 | 5/w | placebo | Same dose |  |  |
| Marley et al. 2021(Marley, Grant, & Babraj, 2021) | United Kingdom | SSIT | 13/0 | 25±5 | 2/w | Vitamin D₃ | 50 000 IU/w | 6w | maximal oxygen uptake; peak power; Average power |
|  |  | C | 14/0 | 25±5 | 2/w | olive oil | 5g/w |  |  |
| Burgos et al. 2022(Burgos et al., 2022) | Spain | MMT | 8/0 | 32.17±4.87 | 7/w | nitrate | 2.1 g/d | 9w | maximal oxygen uptake; |
|  |  | C | 8/0 | 32.17±4.87 | 7/w | placebo | Same dose |  |  |
| Giv et al. 2024(Giv, Aminaei, & Nikoei, 2024) | Iran | SSIT | 10/0 | 23.83 ±2.44 | 3/w | nitrate | 300mg/d | 8w | maximal oxygen uptake; peak power; Average power |
|  |  | C | 10/0 | 24.32±2.44 | 3/w | placebo | Same dose |  |  |
| Tirkey et al. 2021a(Tirkey, Anant, & Venugopal, 2021) | India | ET | 8/0 | 26.3 ± 1.52 |  | nitrate | Beetroot juice 250 ml/day | 15d | Endurance performance |
|  |  | C | 7/0 | 26.3±1.52 |  | placebo |  |  |  |
| Tirkey et al. 2021b(Tirkey et al., 2021) | India | ET | 0/8 | 26.3 ± 1.52 |  | nitrate | Beetroot juice 250 ml/day | 15d | Endurance performance |
|  |  | C | 0/7 | 26.3±1.52 |  | placebo |  |  |  |
| Adji et al. 2022(Adji, Sofro, & Hapsari, 2022) | Indonesia | MMT | 8/0 | 16.25±1.04 | 3/w | nitrate | Beetroot juice 250 ml/day | 2w | maximal oxygen uptake; |
|  |  | C | 8/0 | 15.38±0.74 | 3/w | placebo | 250 ml/d |  |  |
| Townsend et al. 2022(Townsend et al., 2022) | America | RT | 8/0 | 20.5±1.7 | 2-3/w | nitrate | 80mg/d | 11w | peak power; Average power |
|  |  | C | 8/0 | 20.5±1.7 | 2-3/w | placebo | 500mg/d |  |  |
| Chilibeck et al. 2007(Chilibeck, Magnus, & Anderson, 2007) | Canada | MMT | 9/0 | 27.2±2.8 | 2/w | creatine | 0.1 g/kg/day | 8w | Endurance performance |
|  |  | C | 9/0 | 26.4±3.0 | 2/w | glucose | 0.8 g/kg/day |  |  |
| Huang et al. 2017(Huang et al., 2017) | Taiwan, China | ET | 6/0 | 21.7 ± 2.7 | 7/w | protein | 33.5 g/d | 5w | Endurance performance |
|  |  | C | 6/0 | 21.7 ± 2.0 | 7/w | placebo |  |  |  |
| Roberson et al. 2018(Roberson et al., 2018) | America | ET | 5/4 | 33±7 | 3/w | protein | 84 g/d | 10w | Endurance performance |
|  |  | C | 5/3 | 28±10 | 3/w | Sugar pills | 1 g/d |  |  |

Note:RT,Resistance Training;PT,Plyometric;SSIT,Short-Sprint Interval;HIIT,High-Intensity Interval Training;CSE,Concurrent Strength + Endurance;MMT, Multi-Modal Training; ET: endurance training; M, Male; F, Female; w, Week; 　, None.

# References for Table S2.1:

Adji, F. R., Sofro, Z. M., & Hapsari, M. (2022). The effect of beetroot juice (beta vulgaris L.) supplemen-tation on ṼO2max of youth soccer athletes. *Journal of Public Health in Africa*, *13*(s2). https://doi.org/10.4081/jphia.2022.2406

Alabsi, K., Rashidlamir, A., & Dokht, E. H. (2023). The effect of 4 weeks of strength training and beta-alanine supplementation on anaerobic power and carnosine level in boxer players. *Journal of Science in Sport and Exercise*, *5*(1), 62–69. https://doi.org/10.1007/s42978-021-00151-z

Bemben, M., Bemben, D., Loftiss, D., & Knehans, A. (2001). Creatine supplementation during resistance training in college football athletes. *Medicine & Science in Sports & Exercise*, *33*(10), 1667–1673. https://doi.org/10.1097/00005768-200110000-00009

Brisola, G. M. P., Artioli, G. G., Papoti, M., & Zagatto, A. M. (2016). Effects of four weeks of β-alanine supplementation on repeated sprint ability in water polo players. *PLOS One*, *11*(12). https://doi.org/10.1371/journal.pone.0167968

Burgos, J., Viribay, A., Fernández-Lázaro, D., Calleja-González, J., González-Santos, J., & Mielgo-Ayuso, J. (2022). Combined effects of citrulline plus nitrate-rich beetroot extract co-supplementation on maximal and endurance-strength and aerobic power in trained male triathletes: A randomized double-blind, placebo-controlled trial. *Nutrients*, *14*(1). https://doi.org/10.3390/nu14010040

Chilibeck, P. D., Magnus, C., & Anderson, M. (2007). Effect of in-season creatine supplementation on body composition and performance in rugby union football players. *Applied Physiology, Nutrition and Metabolism*, *32*(6), 1052–1057. https://doi.org/10.1139/H07-072

Durkalec-Michalski, K., & Jeszka, J. (2015). The efficacy of a β-hydroxy-β-methylbutyrate supplementation on physical capacity, body composition and biochemical markers in elite rowers: A randomised, double-blind, placebo-controlled crossover study. *Journal of the International Society of Sports Nutrition*, *12*(1), 31. https://doi.org/10.1186/s12970-015-0092-9

Durkalec-Michalski, K., Jeszka, J., & Podgórski, T. (2017). The effect of a 12-week beta-hydroxy-beta-methylbutyrate (HMB) supplementation on highly-trained combat sports athletes: A randomised, double-blind, placebo-controlled crossover study. *Nutrients*, *9*(7), 753. https://doi.org/10.3390/nu9070753

Fairbairn, K. A., Ceelen, I. J. M., Skeaff, C. M., Cameron, C. M., & Perry, T. L. (2018). Vitamin D3 supplementation does not improve sprint performance in professional rugby players: A randomized, placebo-controlled, double-blind intervention study. *International Journal of Sport Nutrition and Exercise Metabolism*, *28*(1), 1–9. https://doi.org/10.1123/ijsnem.2017-0157

Giv, V., Aminaei, M., & Nikoei, R. (2024). The effect of eight weeks beetroot juice supplement on aerobic, anaerobic power, and field performance of soccer players. *Research in Sports Medicine (Print)*, *32*(1), 132–144. https://doi.org/10.1080/15438627.2022.2090250

Guo, W., & Wang, S. (2024). Physiological and performance adaptations to beta alanine supplementation and short sprint interval training in volleyball players. *Scientific Reports*, *14*(1), 16833. https://doi.org/10.1038/s41598-024-67974-y

Hoffman, J. R., Ratamess, N. A., Kang, J., Falvo, M. J., & Faigenbaum, A. D. (2007). Effects of protein supplementation on muscular performance and resting hormonal changes in college football players. *Journal of Sports Science and Medicine*, *6*(1), 85–92.

Hoffman, J., Ratamess, N., Kang, J., Mangine, G., Faigenbaum, A., & Stout, J. (2006). Effect of creatine and β-alanine supplementation on performance and endocrine responses in strength/power athletes. *International Journal of Sport Nutrition and Exercise Metabolism*, *16*(4), 430–446. https://doi.org/10.1123/ijsnem.16.4.430

Huang, W.-C., Chang, Y.-C., Chen, Y.-M., Hsu, Y.-J., Huang, C.-C., Kan, N.-W., & Chen, S.-S. (2017). Whey protein improves marathon-induced injury and exercise performance in elite track runners. *International Journal of Medical Sciences*, *14*(7), 648–654. https://doi.org/10.7150/ijms.19584

Jastrzębska, M., Kaczmarczyk, M., & Jastrzębski, Z. (2016). Effect of vitamin D supplementation on training adaptation in well-trained soccer players. *Journal of Strength & Conditioning Research*, *30*(9), 2648‐2655. https://doi.org/10.1519/JSC.0000000000001337

Jones, A. M., Atter, T., & Georg, K. P. (1999). Oral creatine supplementation improves multiple sprint performance in elite ice-hockey players. *Journal of Sports Medicine and Physical Fitness*, *39*(3), 189–196.

Jung, H. C., Seo, M. W., Lee, S., Jung, S. W., & Song, J. K. (2018). Correcting vitamin D insufficiency improves some but not all aspects of physical performance during winter training in taekwondo athletes. *International Journal of Sport Nutrition and Exercise Metabolism*, *28*(6), 635–643. https://doi.org/10.1123/ijsnem.2017-0412

Li, M., & Sheykhlouvand, M. (2025). Effects of combined versus single supplementation of creatine, beta-alanine, and L-citrulline during short sprint interval training on basketball players’ performance: A double-blind randomized placebo-controlled trial. *International Journal of Sports Physiology and Performance*, ((Li M.) School of Sport Education, Tianjin University of Sports, Tianjin, China), 1–9. https://doi.org/10.1123/ijspp.2024-0310

Li, S.-C., & Zhao, Y.-F. (2007). Effects of carbohydrate and whey protein supplement at appropriate time on physical performance during football game. *Journal of Clinical Rehabilitative Tissue Engineering Research*, *11*(51), 10304–10307.

Marley, A., Grant, M. C., & Babraj, J. (2021). Weekly vitamin D3 supplementation improves aerobic performance in combat sport athletes. *European Journal of Sport Science*, *21*(3), 379–387. https://doi.org/10.1080/17461391.2020.1744736

McIntosh, N., Love, T., Haszard, J., Osborne, H., & Black, K. (2018). β-hydroxy β-methylbutyrate (HMB) supplementation effects on body mass and performance in elite male rugby union players. *Journal of Strength & Conditioning Research*, *32*(1), 19‐26. https://doi.org/10.1519/JSC.0000000000001695

Mhamed, M. B., Zarrouk, F., Mrad, M., Methnani, J., Bahlous, A., Zaouali, M., … Bouhlel, E. (2024). Effects of whey protein on body composition, biochemical profile, and high intensity physical performances in well-trained endurance runners. *Science & Sports*, *39*(7), 588–598. https://doi.org/10.1016/j.scispo.2024.02.001

Murphy, A. J., Watsford, M. L., Coutts, A. J., & Richards, D. A. B. (2005). Effects of creatine supplementation on aerobic power and cardiovascular structure and function. *Journal of Science and Medicine in Sport*, *8*(3), 305–313. https://doi.org/10.1016/S1440-2440(05)80041-6

Norberto, M. S., Barbieri, R. A., Bertucci, D. R., Gobbi, R. B., Campos, E. Z., Zagatto, A. M., … Papoti, M. (2020). Beta alanine supplementation effects on metabolic contribution and swimming performance. *Journal of the International Society of Sports Nutrition*, *17*(1), 40. https://doi.org/10.1186/s12970-020-00365-6

Portal, S., Zadik, Z., Rabinowitz, J., Pilz-Burstein, R., Adler-Portal, D., Meckel, Y., … Nemet, D. (2011). The effect of HMB supplementation on body composition, fitness, hormonal and inflammatory mediators in elite adolescent volleyball players: A prospective randomized, double-blind, placebo-controlled study. *European Journal of Applied Physiology*, *111*(9), 2261–2269. https://doi.org/10.1007/s00421-011-1855-x

Ramírez-Campillo, R., González-Jurado, J. A., Martínez, C., Nakamura, F. Y., Peñailillo, L., Meylan, C. M. P., … Izquierdo, M. (2016). Effects of plyometric training and creatine supplementation on maximal-intensity exercise and endurance in female soccer players. *Journal of Science and Medicine in Sport*, *19*(8), 682–687. https://doi.org/10.1016/j.jsams.2015.10.005

Roberson, P. A., Romero, M. A., Mumford, P. W., Osburn, S. C., Haun, C. T., Vann, C. G., … Roberts, M. D. (2018). Protein supplementation throughout 10 weeks of progressive run training is not beneficial for time trial improvement. *Frontiers in Nutrition*, *5*, 97. https://doi.org/10.3389/fnut.2018.00097

Rosas, F., Ramírez-Campillo, R., Martínez, C., Caniuqueo, A., Cañas-Jamet, R., McCrudden, E., … Izquierdo, M. (2017). Effects of plyometric training and beta-alanine supplementation on maximal-intensity exercise and endurance in female soccer players. *Journal of Human Kinetics*, *58*(1), 99–109. https://doi.org/10.1515/hukin-2017-0072

Tirkey, D., Anant, S. K., & Venugopal, R. (2021). Effect of beetroot supplementation on 10 km time trial performance of distance runners. *Research Journal of Pharmacy and Technology*, *14*(12), 6423–6429. https://doi.org/10.52711/0974-360X.2021.01111

Townsend, J. R., Hart, T. L., Haynes, J. T. 4th, Woods, C. A., Toy, A. M., Pihera, B. C., … Gonzalez, A. M. (2022). Influence of dietary nitrate supplementation on physical performance and body composition following offseason training in division I athletes. *Journal of Dietary Supplements*, *19*(4), 534–549. https://doi.org/10.1080/19390211.2021.1900482

# Appendix 3: Risk of bias of randomized clinical trials

**Figure 2:** Overall risk of bias presented as percentage of each risk of bias item across all included studies. Red = Low risk, Green = High risk, B**lue** = Some concerns.


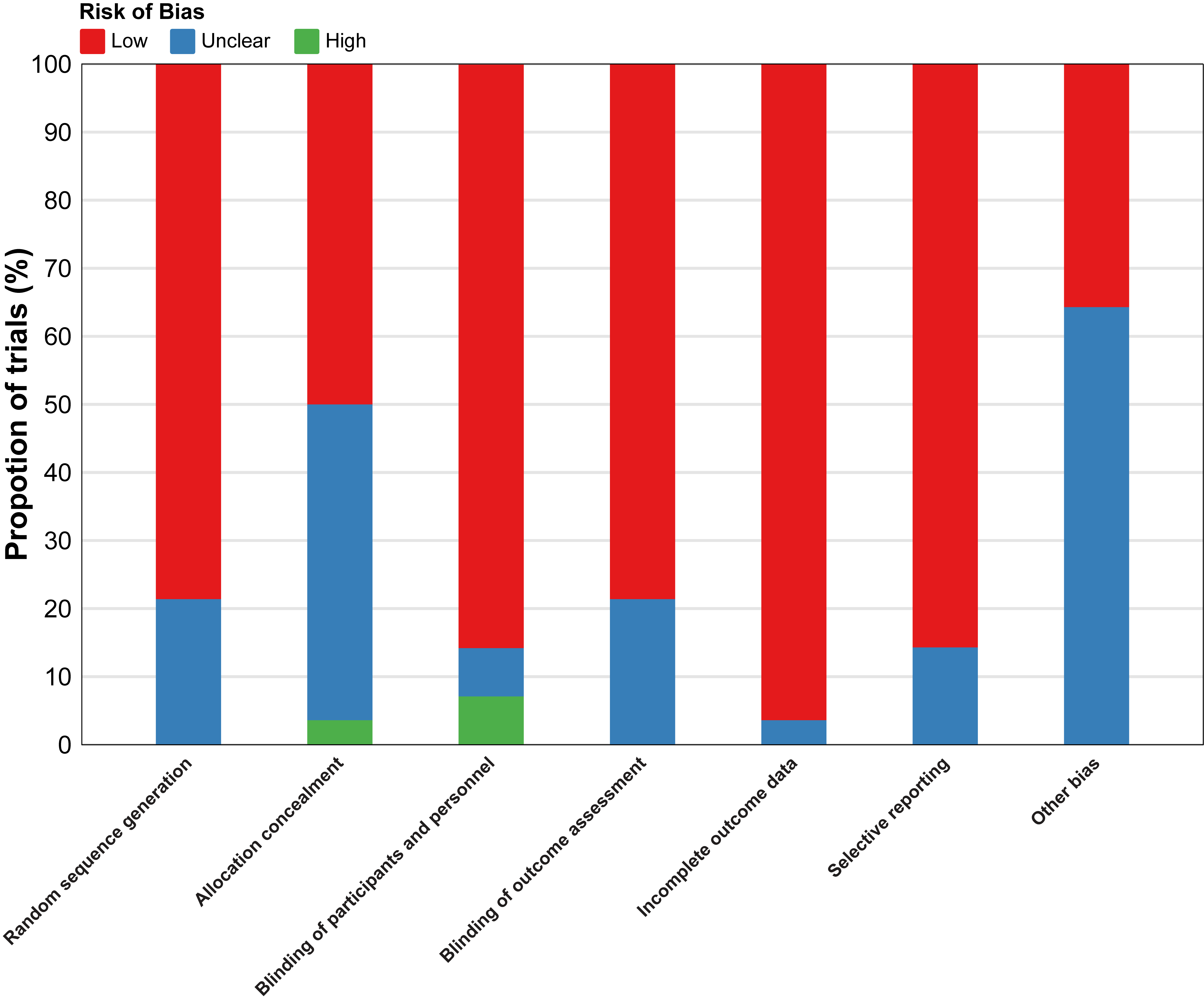


**Figure S2:** Study level risk of bias assessment using Cochrane risk of bias tool 2.0 for assessing risk of bias of randomized clinical trials.


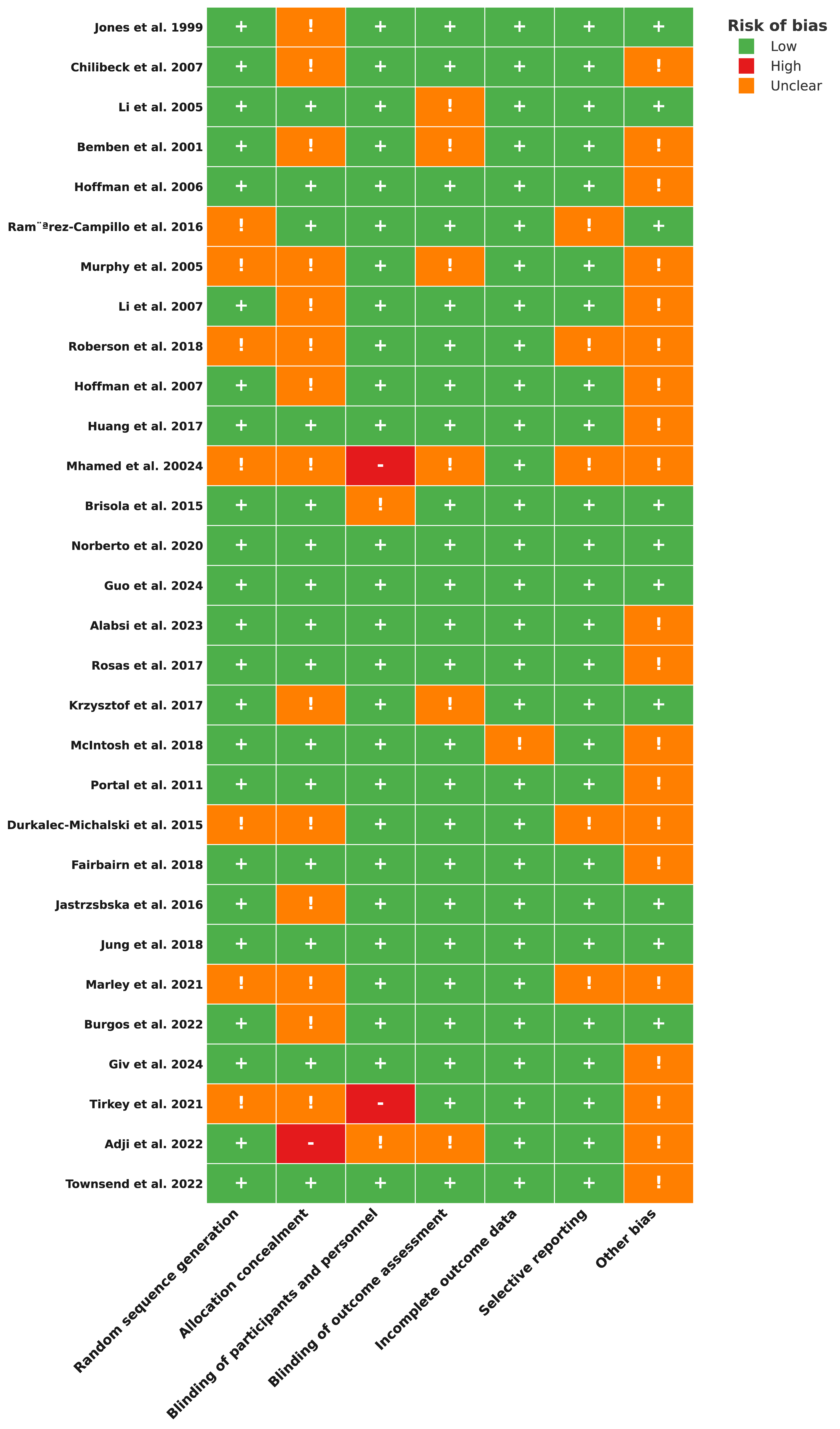


# Appendix 4: Global and Local Inconsistency Tests

**Table S4.1** Global inconsistency test (design-by-treatment interaction) of Peak power

| Outcome code | Coefficient | Std. Err. | z | P>\|z\| | 95% CI Lower | 95% CI Upper |
| --- | --- | --- | --- | --- | --- | --- |
| protein | 0.8525543 | 0.2977121 | 2.86 | 0.004 | 0.2690494 | 1.436059 |
| creatine | 0.6164216 | 0.2130901 | 2.89 | 0.004 | 0.1987727 | 1.03407 |
| β-alanine | 0.5803862 | 0.2505978 | 2.32 | 0.021 | 0.0892235 | 1.071549 |
| Vitamin D3 | 0.4034886 | 0.230249 | 1.75 | 0.08 | -0.0477912 | 0.8547684 |
| HMB | 0.6004724 | 0.175487 | 3.42 | 0.001 | 0.2565241 | 0.9444207 |
| nitrate | 0.4630167 | 0.3034146 | 1.53 | 0.127 | -0.1316649 | 1.057698 |

**Table S4.2** Local inconsistency (node-splitting analysis) of Peak power

| Comparison | Direct Coef. | Direct SE | Indirect Coef. | Indirect SE | Difference | SE (Difference) | P>\|z\| |
| --- | --- | --- | --- | --- | --- | --- | --- |
| placebo–creatine* | 0.6928268 | 0.216393 | -0.7640136 | 0.9497495 | 1.45684 | 0.9811812 | 0.138 |
| placebo–β-alanine* | 0.6118463 | 0.2713203 | 0.3282726 | 0.8603757 | 0.2835737 | 0.9098034 | 0.755 |
| creatine–β-alanine | 0.1213158 | 0.3817215 | -0.2445298 | 0.4396351 | 0.3658456 | 0.5817678 | 0.529 |

**Table S4.3** Global inconsistency test (design-by-treatment interaction) of Mean power

| Outcome code | Coefficient | Std. Err. | z | P>\|z\| | 95% CI Lower | 95% CI Upper |
| --- | --- | --- | --- | --- | --- | --- |
| protein | 0.7355042 | 0.3324668 | 2.21 | 0.027 | 0.0838812 | 1.387127 |
| creatine | 0.7383008 | 0.2373142 | 3.11 | 0.002 | 0.2731735 | 1.203428 |
| β-alanine | 0.752996 | 0.2829833 | 2.66 | 0.008 | 0.198359 | 1.307633 |
| HMB | 0.4523889 | 0.2064566 | 2.19 | 0.028 | 0.0477415 | 0.8570364 |
| nitrate | 0.2561227 | 0.3334474 | 0.77 | 0.442 | -0.3974223 | 0.9096677 |

**Table S4.4** Local inconsistency (node-splitting analysis) of Mean power

| Comparison | Direct Coef. | Direct SE | Indirect Coef. | Indirect SE | Difference | SE (Difference) | P>\|z\| |
| --- | --- | --- | --- | --- | --- | --- | --- |
| placebo–creatine* | 0.7086174 | 0.248323 | 1.388131 | 1.132728 | -0.6795138 | 1.160057 | 0.558 |
| placebo–β-alanine* | 0.716782 | 0.3045961 | 1.133051 | 0.9608191 | -0.4162689 | 1.009303 | 0.68 |
| creatine–β-alanine | -0.0024239 | 0.4286611 | 0.0400276 | 0.5178204 | -0.0424514 | 0.6722313 | 0.95 |

**Table S4.5** Global inconsistency test (design-by-treatment interaction) of VO_2_max

| Outcome code | Coefficient | Std. Err. | z | P>\|z\| | 95% CI Lower | 95% CI Upper |
| --- | --- | --- | --- | --- | --- | --- |
| creatine | 0.1052314 | 0.272895 | 0.39 | 0.7 | -0.429633 | 0.6400958 |
| β-alanine | 0.2245598 | 0.2451129 | 0.92 | 0.36 | -0.2558525 | 0.7049722 |
| HMB | 0.2811148 | 0.168982 | 1.66 | 0.096 | -0.0500838 | 0.6123134 |
| nitrate | 0.0583842 | 0.2789518 | 0.21 | 0.834 | -0.4883513 | 0.6051197 |

**Table S4.6** Local inconsistency (node-splitting analysis) of VO_2_max

| Comparison | Direct Coef. | Direct SE | Indirect Coef. | Indirect SE | Difference | SE (Difference) | P>\|z\| |
| --- | --- | --- | --- | --- | --- | --- | --- |
| placebo–creatine* | 0.1143258 | 0.2858162 | 0.009303 | 0.9368526 | 0.1050228 | 0.9811921 | 0.915 |
| placebo–β-alanine* | 0.2922703 | 0.2513012 | -1.127974 | 1.133946 | 1.420244 | 1.162563 | 0.222 |
| creatine–β-alanine | 0.0016079 | 0.3535535 | 0.4376078 | 0.5813494 | -0.4359999 | 0.6804222 | 0.522 |

**Table S4.7** Global inconsistency test (design-by-treatment interaction) of Endurance Performance

| Outcome code | Coefficient | Std. Err. | z | P>\|z\| | 95% CI Lower | 95% CI Upper |
| --- | --- | --- | --- | --- | --- | --- |
| protein | 0.9944409 | 0.4250195 | 2.34 | 0.019 | 0.1614179 | 1.827464 |
| creatine | -0.4648734 | 0.4026337 | -1.15 | 0.248 | -1.254021 | 0.3242742 |
| β-alanine | -0.0290727 | 0.4060112 | -0.07 | 0.943 | -0.8248399 | 0.7666946 |
| Vitamin D3 | 0.1426086 | 0.3350224 | 0.43 | 0.67 | -0.5140232 | 0.7992405 |
| β-hydroxy-β-methylbutyrate | 0.2941935 | 0.3745718 | 0.79 | 0.432 | -0.4399536 | 1.028341 |
| nitrate | 0.310388 | 0.409018 | 0.76 | 0.448 | -0.4912726 | 1.112049 |

# Appendix 5: Network maps and forest plots of secondary outcomes

Figure S5.1: Network map of the effect on Peak power, and forest plot of network effect sizes for compared with control. The size of the nodes was proportional to the number of participants included in the trial, and the thickness of lines between the interventions relates to the number of studies for that comparison.


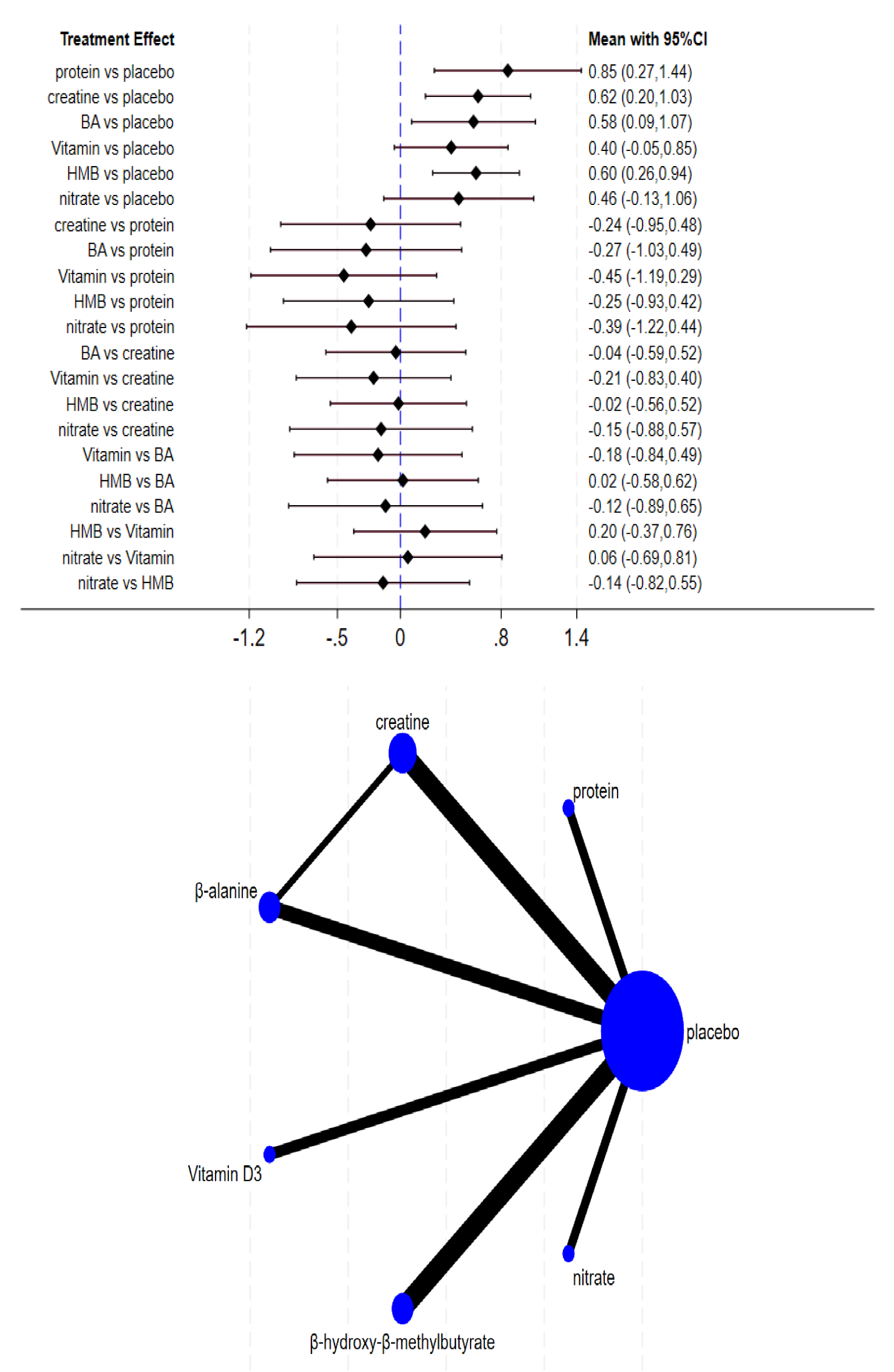


Note: BA  = β-alanine, HMB  =  β-hydroxy-β-methylbutyrate.

**Figure S5.2:** Network map of the effect on Mean Power, and forest plot of network effect sizes for compared with control. The size of the nodes was proportional to the number of participants included in the trial, and the thickness of lines between the interventions relates to the number of studies for that comparison.


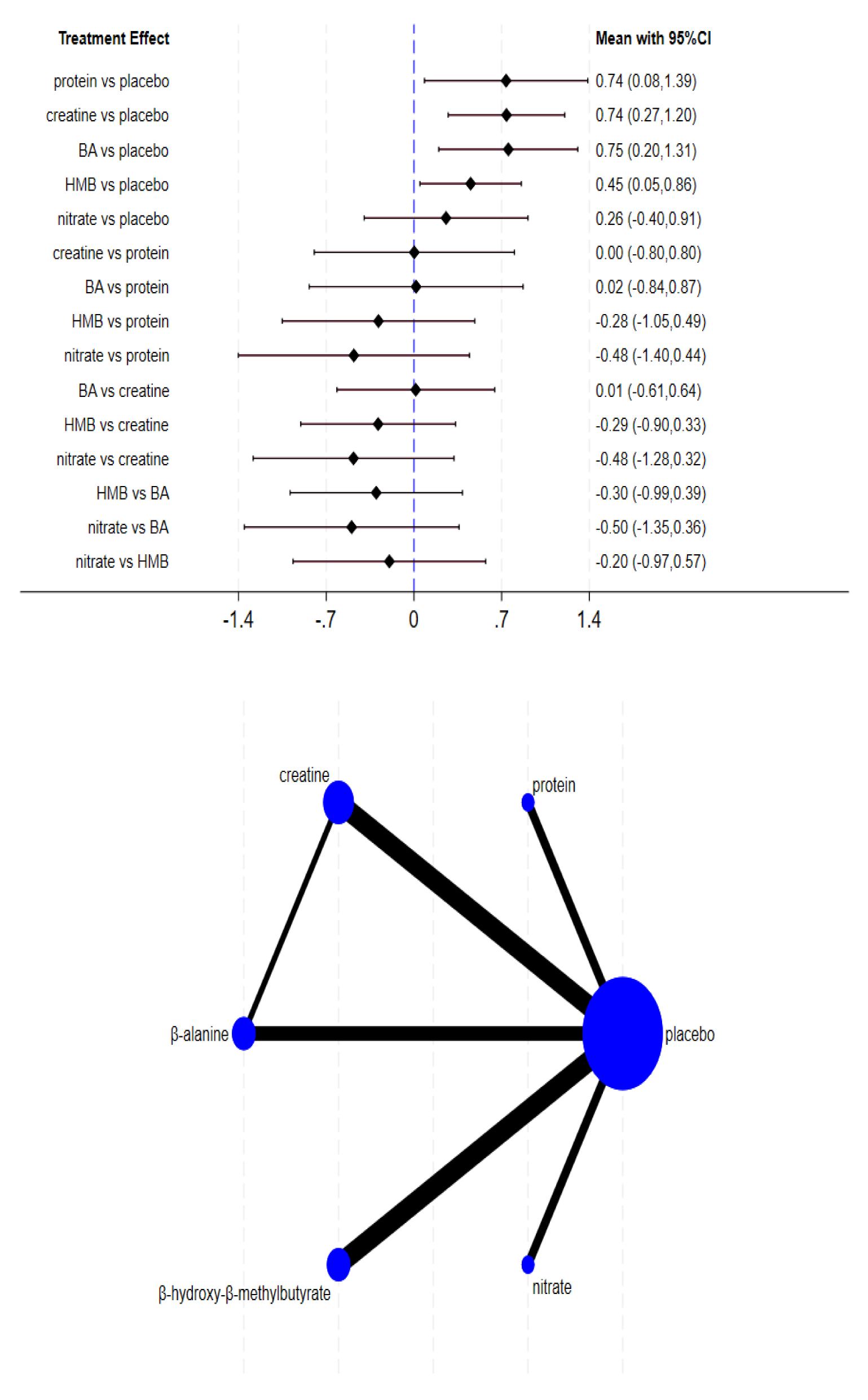


Note: BA  = β-alanine, HMB  =  β-hydroxy-β-methylbutyrate.

**Figure S5.3:** Network map of the effect on VO2max, and forest plot of network effect sizes for compared with control. The size of the nodes was proportional to the number of participants included in the trial, and the thickness of lines between the interventions relates to the number of studies for that comparison.


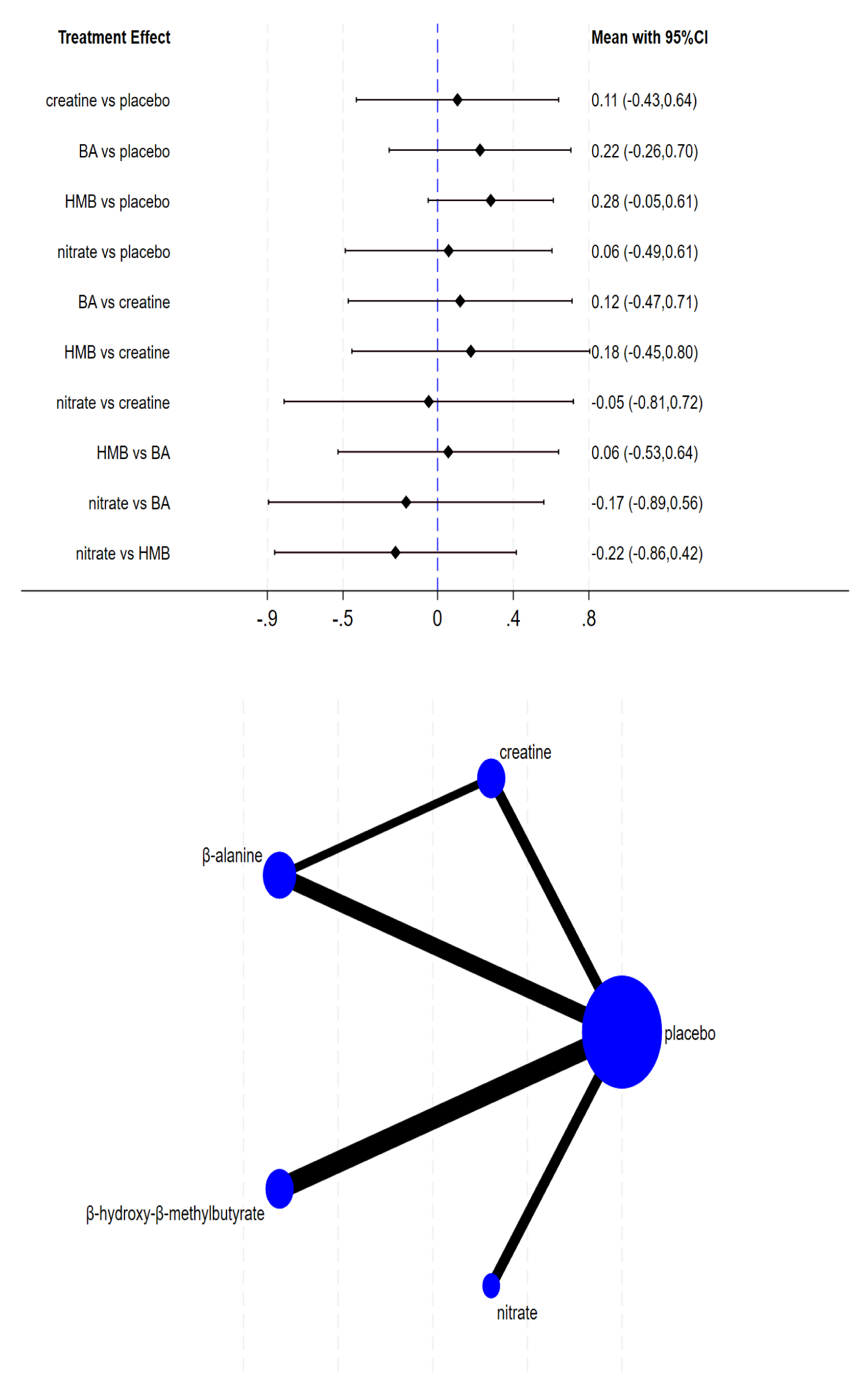


Note: BA  = β-alanine, HMB  =  β-hydroxy-β-methylbutyrate.

**Figure S5.4:** Network map of the effect on Endurance Performance, and forest plot of network effect sizes for compared with control. The size of the nodes was proportional to the number of participants included in the trial, and the thickness of lines between the interventions relates to the number of studies for that comparison.


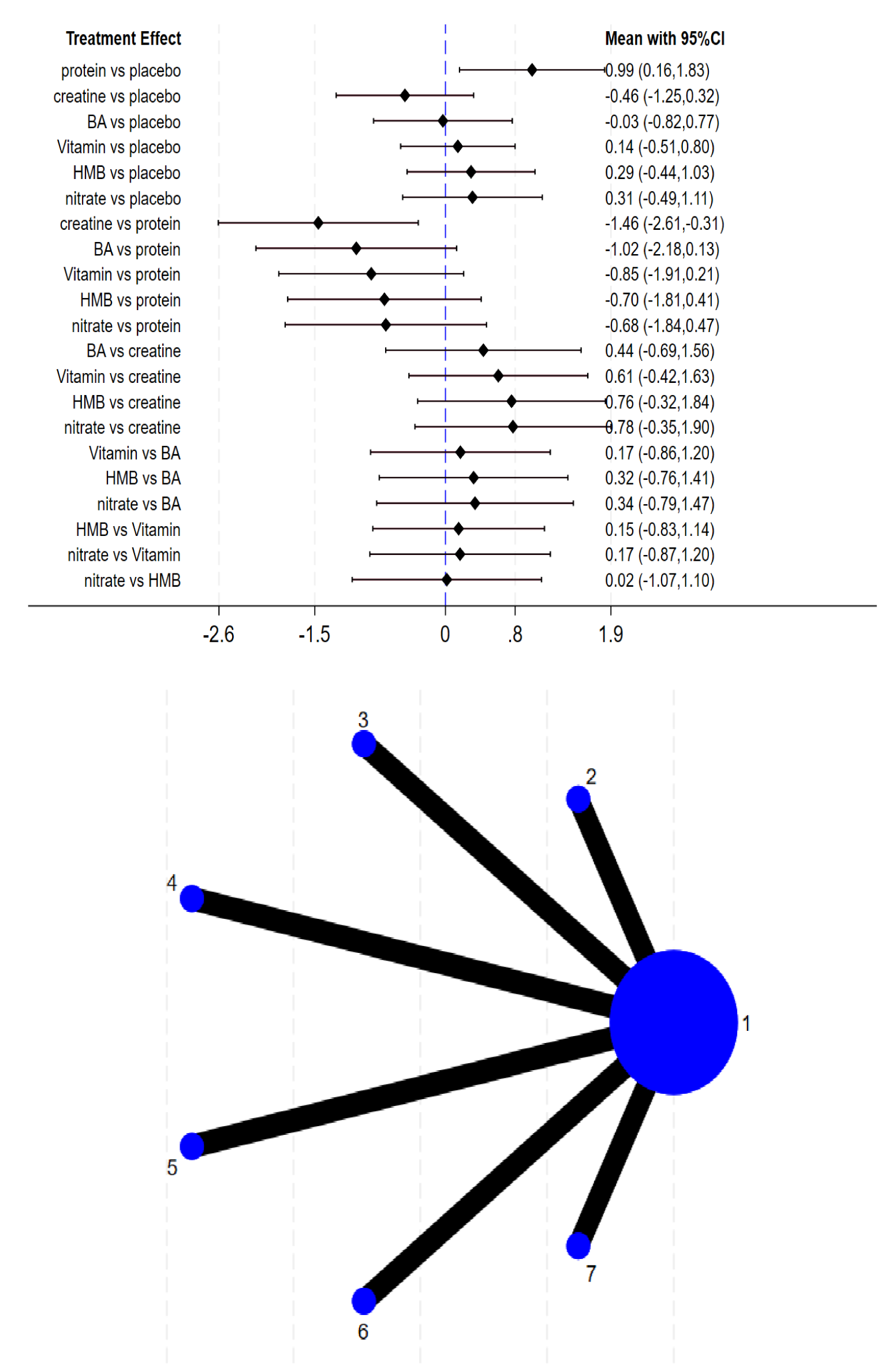


Note: BA  = β-alanine, HMB  =  β-hydroxy-β-methylbutyrate.

# Appendix 6: SUCRA and cumulative probability plots

**Figure S6.1:** The rank heat plot presents a summary of P scores (range 0-100) for each intervention across outcomes. Beta-alanine=β-alanine; HMB=β-hydroxy-β-methylbutyrate.

**Table S6.1:** SUCRA of the effects of different exercise modality on Peak power.

| **Treatment** | **SUCRA** |
| --- | --- |
| protein | 82.9 |
| creatine | 62.5 |
| placebo | 2.1 |
| Vitamin D_3_ | 39 |
| HMB | 60.7 |
| nitrate | 45.4 |
| β-alanine | 57.4 |

**Table S6.1:** SUCRA of the effects of different exercise modality on Mean Power

| **Treatment** | **SUCRA** |
| --- | --- |
| β-alanine | 74.1 |
| creatine | 73.9 |
| Vitamin D | 0 |
| placebo | 5.1 |
| protein | 71 |
| HMB | 45.8 |
| nitrate | 30.2 |

**Table S6.1:** SUCRA of the effects of different exercise modality on VO_2_max

| **Treatment** | **SUCRA** |
| --- | --- |
| β-alanine | 64.2 |
| creatine | 46.3 |
| Vitamin D | 0 |
| placebo | 25.2 |
| protein | 0 |
| HMB | 74.4 |

**Table S6.1:** SUCRA of the effects of different exercise modality on Endurance Performance

| **Treatment** | **SUCRA** |
| --- | --- |
| β-alanine | 36.2 |
| creatine | 10.7 |
| Vitamin D | 50.2 |
| placebo | 36.6 |
| protein | 94.3 |
| HMB | 60 |
| nitrate | 61.9 |

# Appendix 7: League Table of Summary Estimates for Dietary Supplementation Combined with Strength and Conditioning on Athletic Performance from on Network Meta-Analysis

**Table S7.1**: league table of Peak power

The columns represent the comparison of the row exercise modality to the column exercise modality. The rows represent the comparison of the row exercise modality to the column exercise modality. The effect estimates are expressed as mean difference and 95% confidence interval. Mean difference <0 favors the exercise modality in the column, and mean difference >0 favors the exercise modality in the row.

| **protein** |  |  |  |  |  |  |
| --- | --- | --- | --- | --- | --- | --- |
| **0.85 (0.27,1.44)** | placebo |  |  |  |  |  |
| **0.39 (-0.44,1.22)** | -0.46 (-1.06,0.13) | nitrate |  |  |  |  |
| **0.24 (-0.48,0.95)** | -0.62 (-1.03,-0.20) | -0.15 (-0.88,0.57) | creatine |  |  |  |
| **0.45 (-0.29,1.19)** | -0.40 (-0.85,0.05) | 0.06 (-0.69,0.81) | 0.21 (-0.40,0.83) | Vitamin |  |  |
| **0.25 (-0.42,0.93)** | -0.60 (-0.94,-0.26) | -0.14 (-0.82,0.55) | 0.02 (-0.52,0.56) | -0.20 (-0.76,0.37) | HMB |  |
| **0.27 (-0.49,1.03)** | -0.58 (-1.07,-0.09) | -0.12(-0.89,0.65) | 0.04 (-0.52,0.59) | -0.18 (-0.84,0.49) | 0.02 (-0.58,0.62) | β-alanine |

**Table S7.2**: league table of Mean Power

The columns represent the comparison of the row exercise modality to the column exercise modality. The rows represent the comparison of the row exercise modality to the column exercise modality. The effect estimates are expressed as mean difference and 95% confidence interval. Mean difference <0 favors the exercise modality in the column, and mean difference >0 favors the exercise modality in the row.

| **protein** |  |  |  |  |  |
| --- | --- | --- | --- | --- | --- |
| **0.74****(0.08,1.39)** | placebo |  |  |  |  |
| **0.48** **(-0.44,1.40)** | -0.26(-0.91,0.40) | nitrate |  |  |  |
| **-0.00** **(-0.80,0.80)** | -0.74 (-1.20,-0.27) | -0.48 (-1.28,0.32) | creatine |  |  |
| **0.28** **(-0.49,1.05)** | -0.45 (-0.86,-0.05) | -0.20 (-0.97,0.57) | 0.29 (-0.33,0.90) | HMB |  |
| **-0.02** **(-0.87,0.84)** | -0.75 (-1.31,-0.20) | -0.50 (-1.35,0.36) | -0.01 (-0.64,0.61) | -0.30 (-0.99,0.39) | β-alanine |

**Table S7.3**: league table of VO_2_max

The columns represent the comparison of the row exercise modality to the column exercise modality. The rows represent the comparison of the row exercise modality to the column exercise modality. The effect estimates are expressed as mean difference and 95% confidence interval. Mean difference <0 favors the exercise modality in the column, and mean difference >0 favors the exercise modality in the row.

| **placebo** |  |  |  |  |
| --- | --- | --- | --- | --- |
| **-0.06**  **(-0.61,0.49)** | nitrate |  |  |  |
| **-0.11**  **(-0.64,0.43)** | -0.05  (-0.81,0.72) | creatine |  |  |
| **-0.28**  **(-0.61,0.05)** | -0.22  (-0.86,0.42) | -0.18  (-0.80,0.45) | HMB |  |
| **-0.22**  **(-0.70,0.26)** | -0.17  (-0.89,0.56) | -0.12  (-0.71,0.47) | 0.06  (-0.53,0.64) | β-alanine |

**Table S7.3**: league table of Endurance Performance

The columns represent the comparison of the row exercise modality to the column exercise modality. The rows represent the comparison of the row exercise modality to the column exercise modality. The effect estimates are expressed as mean difference and 95% confidence interval. Mean difference <0 favors the exercise modality in the column, and mean difference >0 favors the exercise modality in the row.

| **protein** |  |  |  |  |  |  |
| --- | --- | --- | --- | --- | --- | --- |
| **0.99 (0.16,1.83)** | placebo |  |  |  |  |  |
| **0.68 (-0.47,1.84)** | -0.31 (-1.11,0.49) | nitrate |  |  |  |  |
| **1.46 (0.31,2.61)** | 0.46 (-0.32,1.25) | 0.78 (-0.35,1.90) | creatine |  |  |  |
| **0.85 (-0.21,1.91)** | -0.14 (-0.80,0.51) | 0.17 (-0.87,1.20) | -0.61 (-1.63,0.42) | Vitamin |  |  |
| **0.70 (-0.41,1.81)** | -0.29 (-1.03,0.44) | 0.02 (-1.07,1.10) | -0.76 (-1.84,0.32) | -0.15 (-1.14,0.83) | HMB |  |
| **1.02 (-0.13,2.18)** | 0.03 (-0.77,0.82) | 0.34 (-0.79,1.47) | -0.44 (-1.56,0.69) | 0.17 (-0.86,1.20) | 0.32 (-0.76,1.41) | β-alanine |

# Appendix 8: CINeMA Assessment

We use the CINeMA framework to assess evidence certainty, evaluating each network estimate based on the following criteria:

- **Within study bias:** We classified the overall risk of bias for each study as low risk of bias, the risk of bias as moderate when none of the four assessed risk of bias items were rated as high risk, and the risk of bias as high when one or both items were rated as high risk. See **Appendix 3** for the bias assessment.
- **Reporting bias:** We judged it visually by a funnel plot **(Appendix 9)**.
- **Indirectness:** Indirectness was judged low when all included studies directly matched our predefined PICO framework in terms of patient population, interventions, comparators, and outcome measurements, with no substantive deviations from the protocol.
- **Imprecision:** We use the CINeMA website to grade the accuracy of each comparison.
- **Heterogeneity:** We assessed the degree of worry by comparing clinical reasoning based on 95% confidence intervals (CIs) while applying the same clinical reasoning framework as for inaccuracy. In particular, we judged the consistency of our findings based on the confidence and prediction intervals associated with clinically important effect sizes. And we used the same thresholds of clinical significance as described above and followed the recommendations automatically provided by CINeMA (https://cinema.ispm.unibe.ch/).
- **Inconsistency:** For inconsistency, we looked at the results for node splitting (Appendix 4).

**Figure S8.1:** Risk of bias contribution by intervention group in Peak power


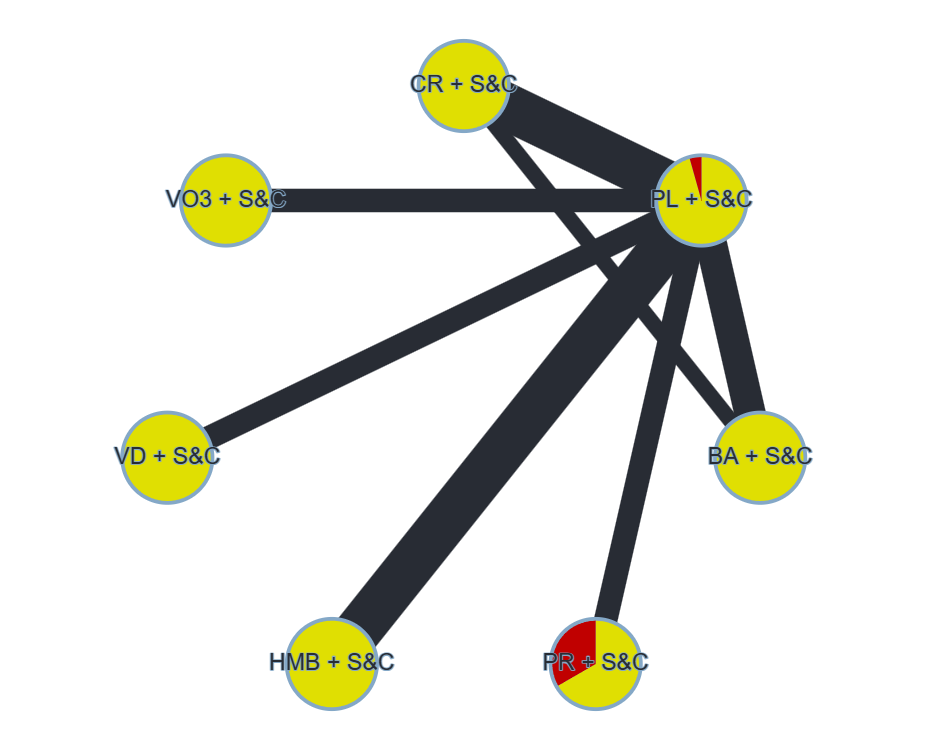


Note. PR = protein; CR = creatine; BA = β-alanine; HMB = β-hydroxy-β-methylbutyrate; VD = vitamin D₃; VO₃ = nitrate; PL = placebo; S&C = strength and conditioning.

**Figure S8.2:** Overall risk of bias by treatment comparison in Peak power

#
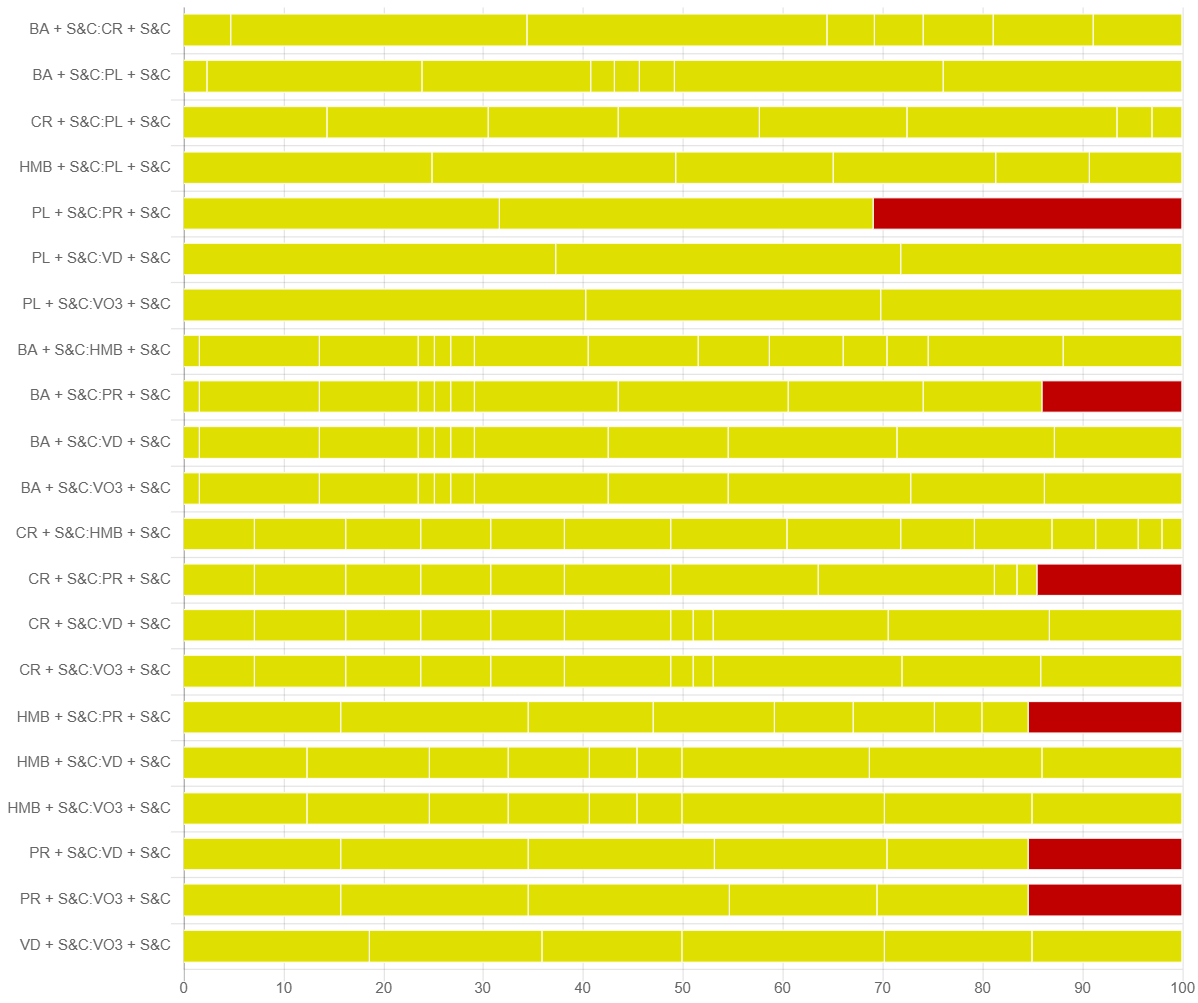


**Figure S8.3:** Risk of bias contribution by intervention group in Mean Power


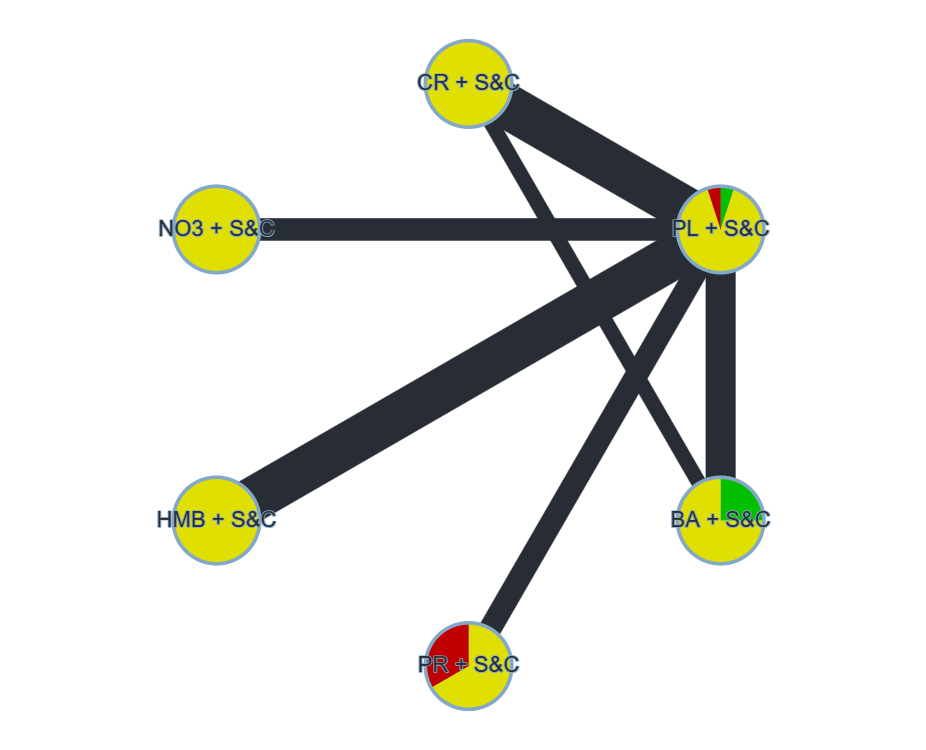


Note: PR = protein; CR = creatine; BA = β-alanine; HMB = β-hydroxy-β-methylbutyrate; VD = vitamin D₃; VO₃ = nitrate; PL = placebo; S&C = strength and conditioning.

**Figure S8.4:** Overall risk of bias by treatment comparison in Mean Power


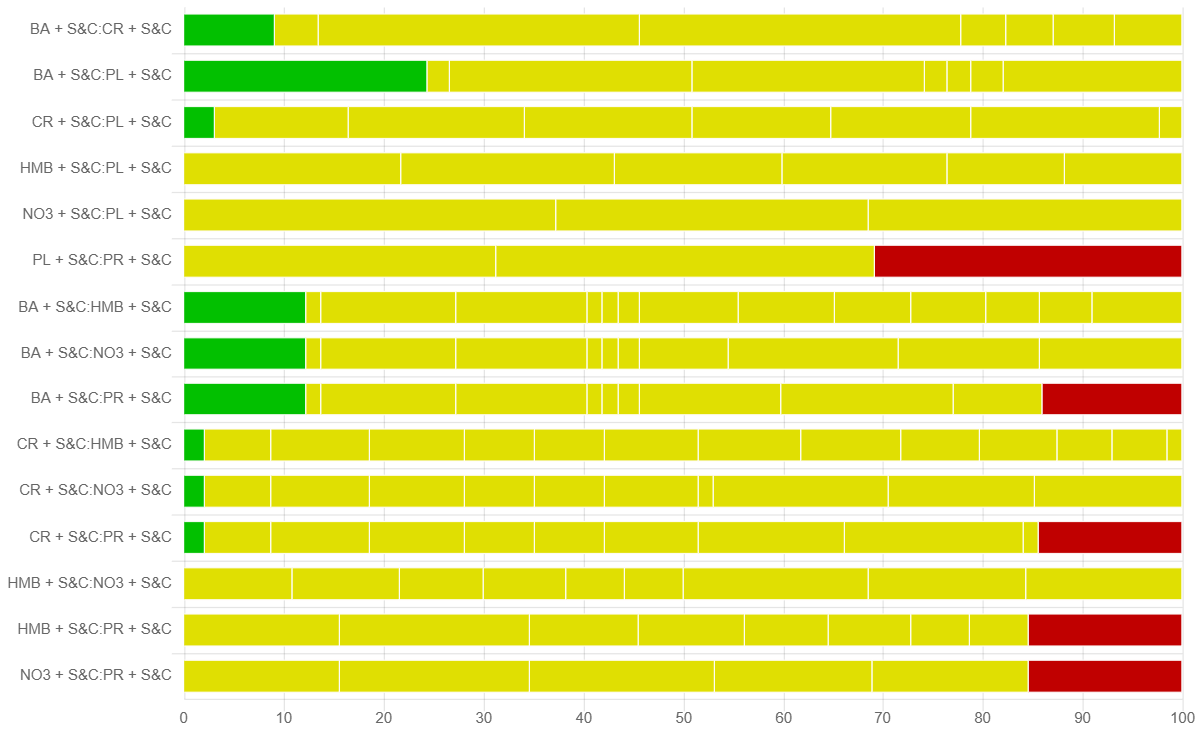


**Figure S8.5**: Risk of bias contribution by intervention group in VO_2_max


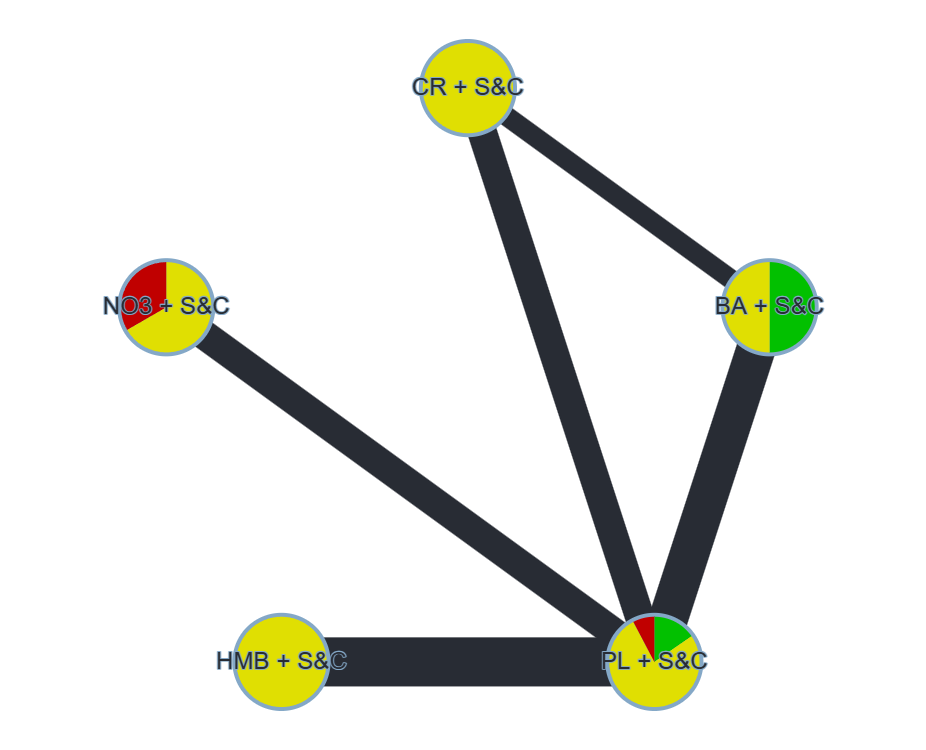


Note: PR = protein; CR = creatine; BA = β-alanine; HMB = β-hydroxy-β-methylbutyrate; VD = vitamin D₃; VO₃ = nitrate; PL = placebo; S&C = strength and conditioning.

**Figure S8.6:** Overall risk of bias by treatment comparison in VO_2_max


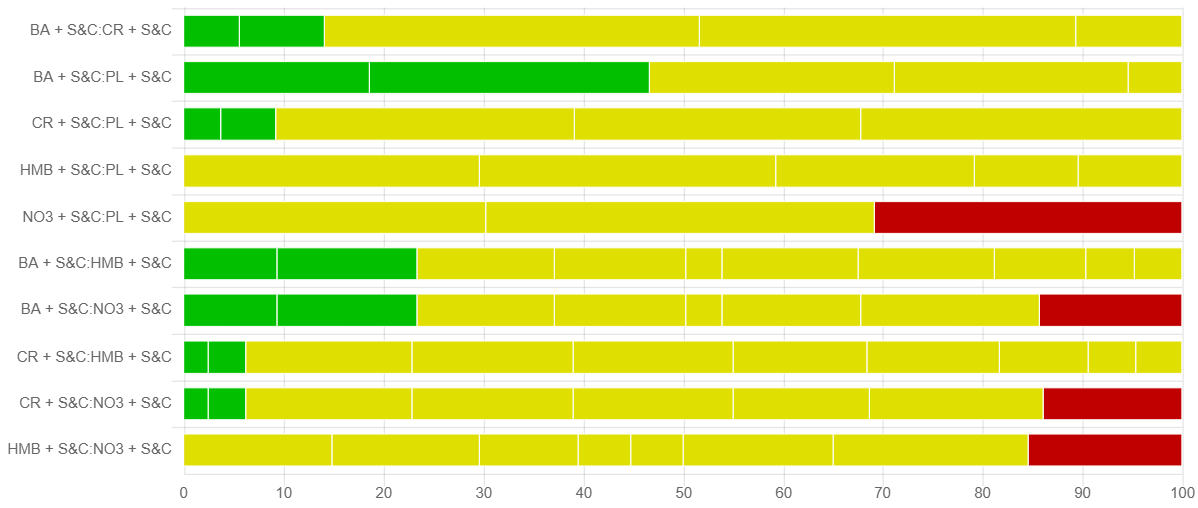


**Figure S8.7:** Risk of bias contribution by intervention group in Endurance Performance


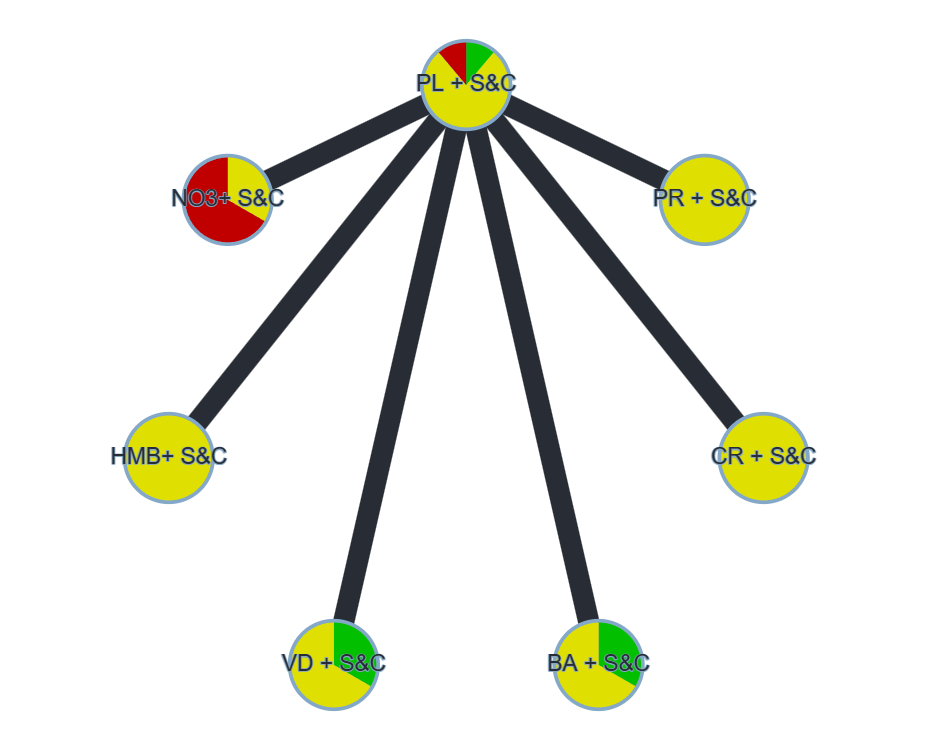


Note: PR = protein; CR = creatine; BA = β-alanine; HMB = β-hydroxy-β-methylbutyrate; VD = vitamin D₃; VO₃ = nitrate; PL = placebo; S&C = strength and conditioning.

**Figure S8.8:** Overall risk of bias by treatment comparison in Endurance Performance


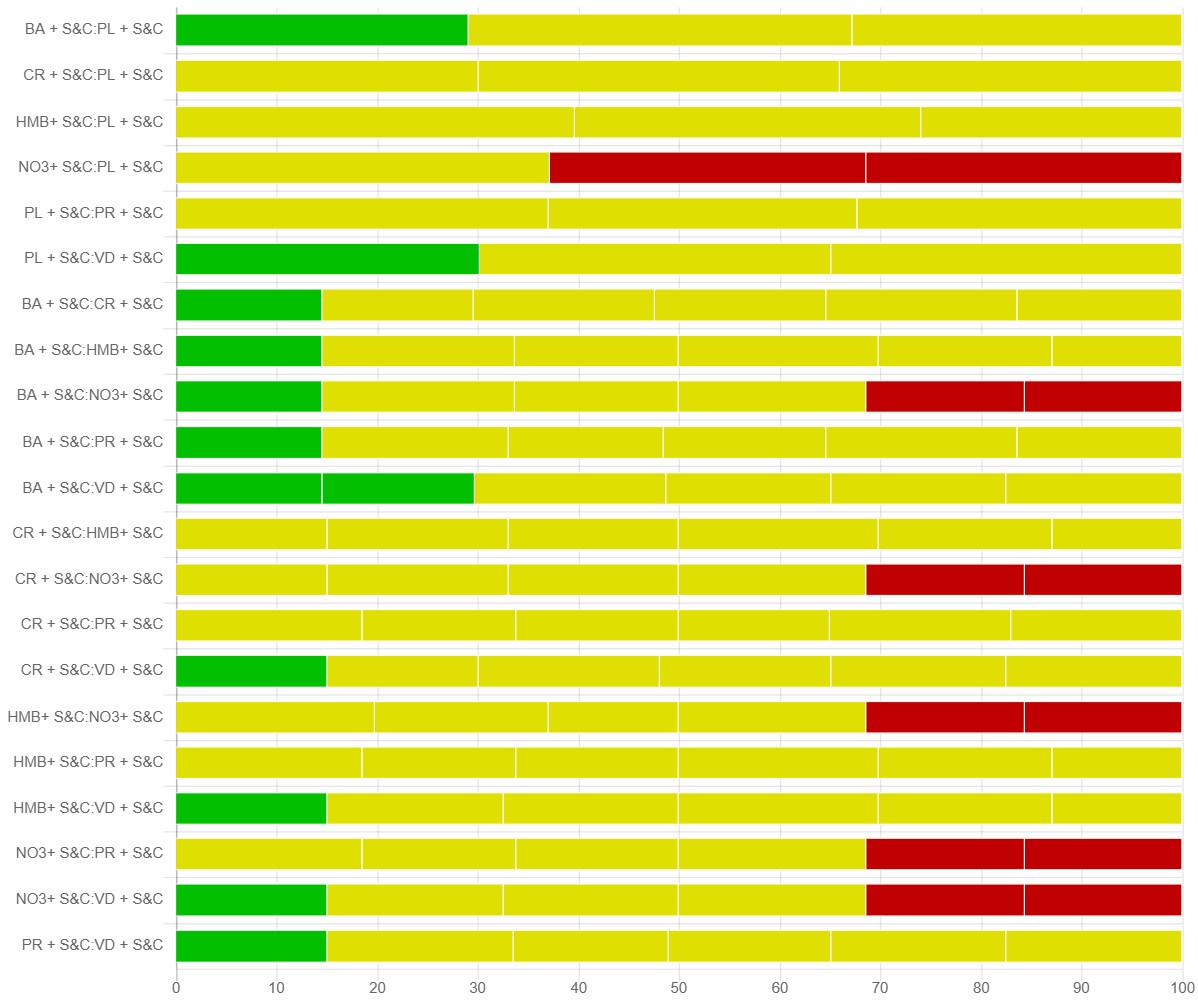


**Table S8.1:** CINeMA Results of muscle strength

| Comparison | Within-study bias | Reporting bias | Indirectness | Imprecision | Heterogeneity | Incoherence | Confidence rating |
| --- | --- | --- | --- | --- | --- | --- | --- |
| BA + S&C:CR + S&C | Some concerns | Low risk | No concerns | Major concerns | No concerns | No concerns | Very low |
| BA + S&C:PL + S&C | Some concerns | Low risk | No concerns | No concerns | Major concerns | No concerns | Very low |
| CR + S&C:PL + S&C | Some concerns | Low risk | No concerns | No concerns | No concerns | No concerns | Moderate |
| HMB + S&C:PL + S&C | Some concerns | Low risk | No concerns | No concerns | No concerns | No concerns | Moderate |
| PL + S&C:PR + S&C | Some concerns | Low risk | No concerns | No concerns | No concerns | No concerns | Moderate |
| PL + S&C:VD + S&C | Some concerns | Low risk | No concerns | Major concerns | No concerns | No concerns | Very low |
| PL + S&C:VO3 + S&C | Some concerns | Low risk | No concerns | Major concerns | No concerns | No concerns | Very low |
| BA + S&C:HMB + S&C | Some concerns | Low risk | No concerns | Major concerns | No concerns | No concerns | Very low |
| BA + S&C:PR + S&C | Some concerns | Low risk | No concerns | Major concerns | No concerns | No concerns | Very low |
| BA + S&C:VD + S&C | Some concerns | Low risk | No concerns | Major concerns | No concerns | No concerns | Very low |
| BA + S&C:VO3 + S&C | Some concerns | Low risk | No concerns | Major concerns | No concerns | No concerns | Very low |
| CR + S&C:HMB + S&C | Some concerns | Low risk | No concerns | Major concerns | No concerns | No concerns | Very low |
| CR + S&C:PR + S&C | Some concerns | Low risk | No concerns | Major concerns | No concerns | No concerns | Very low |
| CR + S&C:VD + S&C | Some concerns | Low risk | No concerns | Major concerns | No concerns | No concerns | Very low |
| CR + S&C:VO3 + S&C | Some concerns | Low risk | No concerns | Major concerns | No concerns | No concerns | Very low |
| HMB + S&C:PR + S&C | Some concerns | Low risk | No concerns | Major concerns | No concerns | No concerns | Very low |
| HMB + S&C:VD + S&C | Some concerns | Low risk | No concerns | Major concerns | No concerns | No concerns | Very low |
| HMB + S&C:VO3 + S&C | Some concerns | Low risk | No concerns | Major concerns | No concerns | No concerns | Very low |
| PR + S&C:VD + S&C | Some concerns | Low risk | No concerns | No concerns | Major concerns | No concerns | Very low |
| PR + S&C:VO3 + S&C | Some concerns | Low risk | No concerns | Major concerns | No concerns | No concerns | Very low |
| VD + S&C:VO3 + S&C | Some concerns | Low risk | No concerns | Major concerns | No concerns | No concerns | Very low |
| BA + S&C:VO3 + S&C | Some concerns | Low risk | No concerns | Major concerns | No concerns | No concerns | Very low |
| CR + S&C:HMB + S&C | Some concerns | Low risk | No concerns | Major concerns | No concerns | No concerns | Very low |

**Table S8.2:** CINeMA Results of jumping performance

| Comparison | Within-study bias | Reporting bias | Indirectness | Imprecision | Heterogeneity | Incoherence | Confidence rating |
| --- | --- | --- | --- | --- | --- | --- | --- |
| BA + S&C:CR + S&C | Some concerns | Low risk | No concerns | Major concerns | No concerns | No concerns | Very low |
| BA + S&C:PL + S&C | Some concerns | Low risk | No concerns | No concerns | Major concerns | No concerns | Very low |
| CR + S&C:PL + S&C | Some concerns | Low risk | No concerns | No concerns | Major concerns | No concerns | Very low |
| HMB + S&C:PL + S&C | Some concerns | Low risk | No concerns | No concerns | Major concerns | No concerns | Very low |
| NO3 + S&C:PL + S&C | Some concerns | Low risk | No concerns | Major concerns | No concerns | No concerns | Very low |
| PL + S&C:PR + S&C | Some concerns | Low risk | No concerns | No concerns | Major concerns | No concerns | Very low |
| BA + S&C:HMB + S&C | Some concerns | Low risk | No concerns | Major concerns | No concerns | No concerns | Very low |
| BA + S&C:NO3 + S&C | Some concerns | Low risk | No concerns | Major concerns | No concerns | No concerns | Very low |
| BA + S&C:PR + S&C | Some concerns | Low risk | No concerns | Major concerns | No concerns | No concerns | Very low |
| CR + S&C:HMB + S&C | Some concerns | Low risk | No concerns | Major concerns | No concerns | No concerns | Very low |
| CR + S&C:NO3 + S&C | Some concerns | Low risk | No concerns | Major concerns | No concerns | No concerns | Very low |
| CR + S&C:PR + S&C | Some concerns | Low risk | No concerns | Major concerns | No concerns | No concerns | Very low |
| HMB + S&C:NO3 + S&C | Some concerns | Low risk | No concerns | Major concerns | No concerns | No concerns | Very low |
| HMB + S&C:PR + S&C | Some concerns | Low risk | No concerns | Major concerns | No concerns | No concerns | Very low |
| NO3 + S&C:PR + S&C | Some concerns | Low risk | No concerns | Major concerns | No concerns | No concerns | Very low |

**Table S8.3:** CINeMA Results of sprinting speed

| Comparison | Within-study bias | Reporting bias | Indirectness | Imprecision | Heterogeneity | Incoherence | Confidence rating |
| --- | --- | --- | --- | --- | --- | --- | --- |
| BA + S&C:CR + S&C | Some concerns | Low risk | No concerns | Major concerns | No concerns | No concerns | Very low |
| BA + S&C:PL + S&C | Some concerns | Low risk | No concerns | Major concerns | No concerns | No concerns | Very low |
| CR + S&C:PL + S&C | Some concerns | Low risk | No concerns | Major concerns | No concerns | No concerns | Very low |
| HMB + S&C:PL + S&C | Some concerns | Low risk | No concerns | Major concerns | No concerns | No concerns | Very low |
| NO3 + S&C:PL + S&C | Some concerns | Low risk | No concerns | Major concerns | No concerns | No concerns | Very low |
| BA + S&C:HMB + S&C | Some concerns | Low risk | No concerns | Major concerns | No concerns | No concerns | Very low |
| BA + S&C:NO3 + S&C | Some concerns | Low risk | No concerns | Major concerns | No concerns | No concerns | Very low |
| CR + S&C:HMB + S&C | Some concerns | Low risk | No concerns | Major concerns | No concerns | No concerns | Very low |
| CR + S&C:NO3 + S&C | Some concerns | Low risk | No concerns | Major concerns | No concerns | No concerns | Very low |
| HMB + S&C:NO3 + S&C | Some concerns | Low risk | No concerns | Major concerns | No concerns | No concerns | Very low |
| BA + S&C:CR + S&C | Some concerns | Low risk | No concerns | Major concerns | No concerns | No concerns | Very low |

**Table S8.4:** CINeMA Results of muscle mass

| Comparison | Within-study bias | Reporting bias | Indirectness | Imprecision | Heterogeneity | Incoherence | Confidence rating |
| --- | --- | --- | --- | --- | --- | --- | --- |
| BA + S&C:PL + S&C | Some concerns | Low risk | No concerns | Major concerns | No concerns | Major concerns | Very low |
| CR + S&C:PL + S&C | Some concerns | Low risk | No concerns | Major concerns | No concerns | Major concerns | Very low |
| HMB+ S&C:PL + S&C | Some concerns | Low risk | No concerns | Major concerns | No concerns | Major concerns | Very low |
| NO3+ S&C:PL + S&C | Major concerns | Low risk | No concerns | Major concerns | No concerns | Major concerns | Very low |
| PL + S&C:PR + S&C | Some concerns | Low risk | No concerns | No concerns | Major concerns | Major concerns | Very low |
| PL + S&C:VD + S&C | Some concerns | Low risk | No concerns | Major concerns | No concerns | Major concerns | Very low |
| BA + S&C:CR + S&C | Some concerns | Low risk | No concerns | Major concerns | No concerns | Major concerns | Very low |
| BA + S&C:HMB+ S&C | Some concerns | Low risk | No concerns | Major concerns | No concerns | Major concerns | Very low |
| BA + S&C:NO3+ S&C | Some concerns | Low risk | No concerns | Major concerns | No concerns | Major concerns | Very low |
| BA + S&C:PR + S&C | Some concerns | Low risk | No concerns | Major concerns | No concerns | Major concerns | Very low |
| BA + S&C:VD + S&C | Some concerns | Low risk | No concerns | Major concerns | No concerns | Major concerns | Very low |
| CR + S&C:HMB+ S&C | Some concerns | Low risk | No concerns | Major concerns | No concerns | Major concerns | Very low |
| CR + S&C:NO3+ S&C | Some concerns | Low risk | No concerns | Major concerns | No concerns | Major concerns | Very low |
| CR + S&C:PR + S&C | Some concerns | Low risk | No concerns | No concerns | Major concerns | Major concerns | Very low |
| CR + S&C:VD + S&C | Some concerns | Low risk | No concerns | Major concerns | No concerns | Major concerns | Very low |
| HMB+ S&C:NO3+ S&C | Some concerns | Low risk | No concerns | Major concerns | No concerns | Major concerns | Very low |
| HMB+ S&C:PR + S&C | Some concerns | Low risk | No concerns | Major concerns | No concerns | Major concerns | Very low |
| HMB+ S&C:VD + S&C | Some concerns | Low risk | No concerns | Major concerns | No concerns | Major concerns | Very low |
| NO3+ S&C:PR + S&C | Some concerns | Low risk | No concerns | Major concerns | No concerns | Major concerns | Very low |
| NO3+ S&C:VD + S&C | Some concerns | Low risk | No concerns | Major concerns | No concerns | Major concerns | Very low |
| PR + S&C:VD + S&C | Some concerns | Low risk | No concerns | Major concerns | No concerns | Major concerns | Very low |

# Appendix 9: Funnel plots

**Figure S9.1:** Funnel plot of Peak power


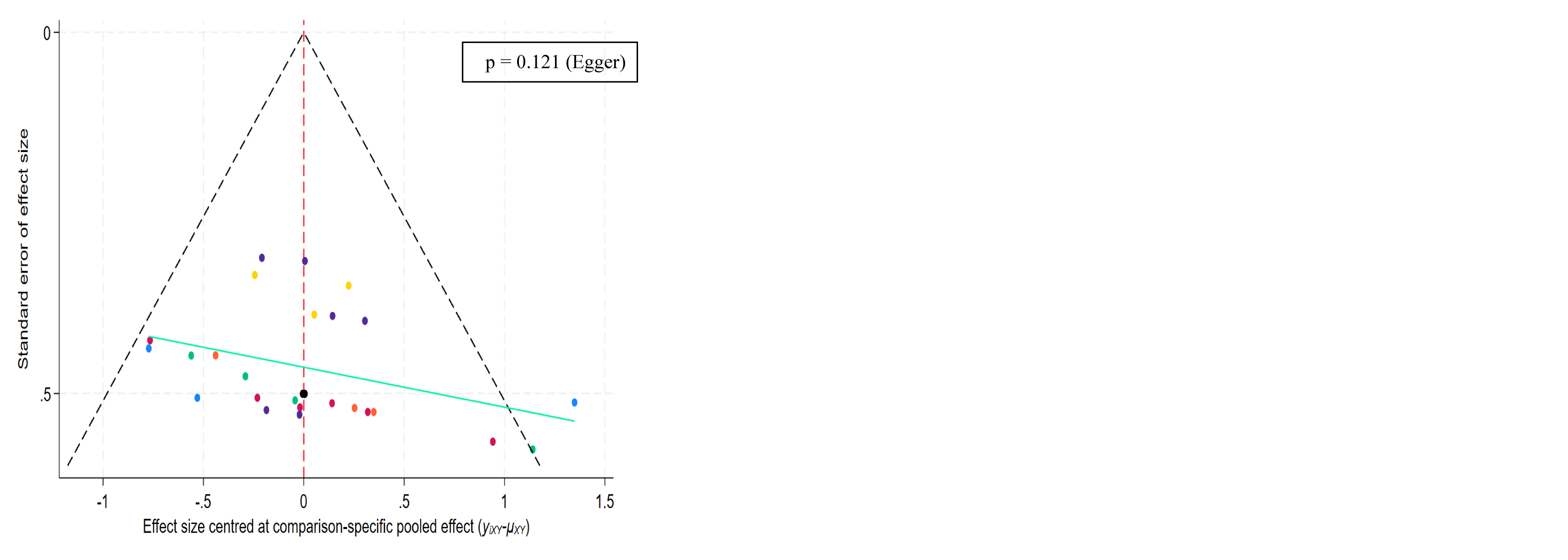


**Figure S9.2:** Funnel plot of Mean Power


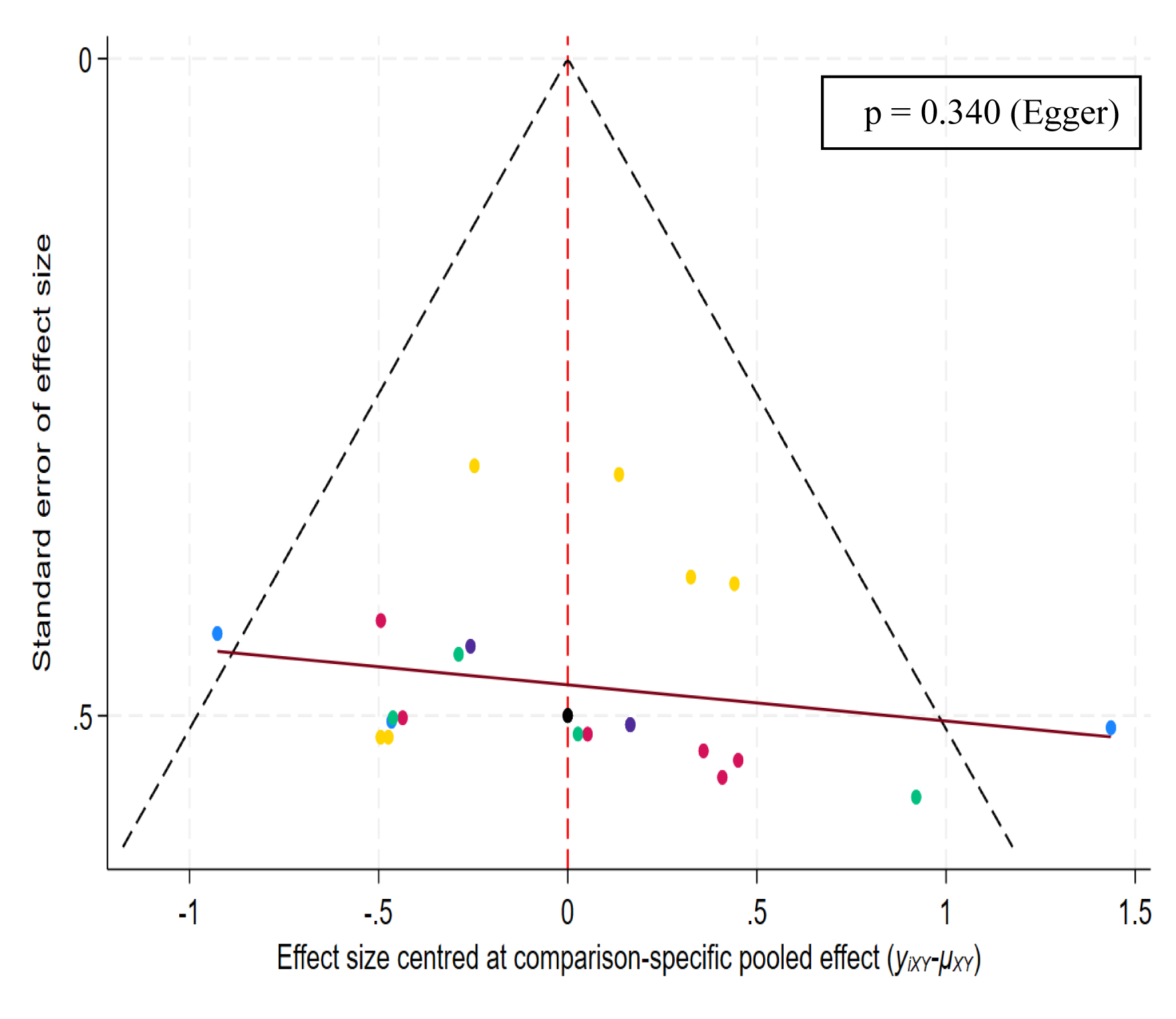


**Figure S9.3:** Funnel plot of VO_2_max


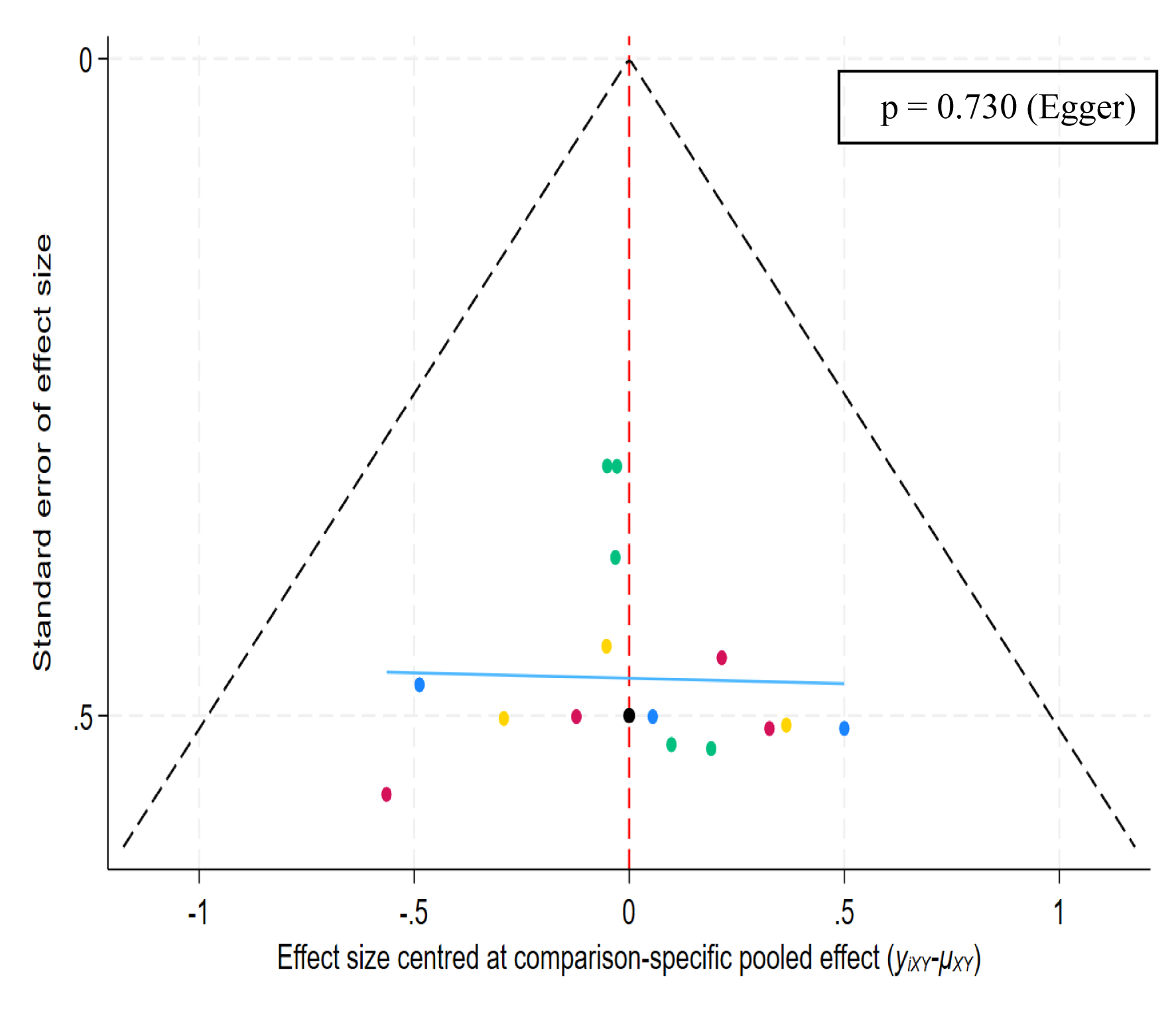


**Figure S9.4:** Endurance Performance


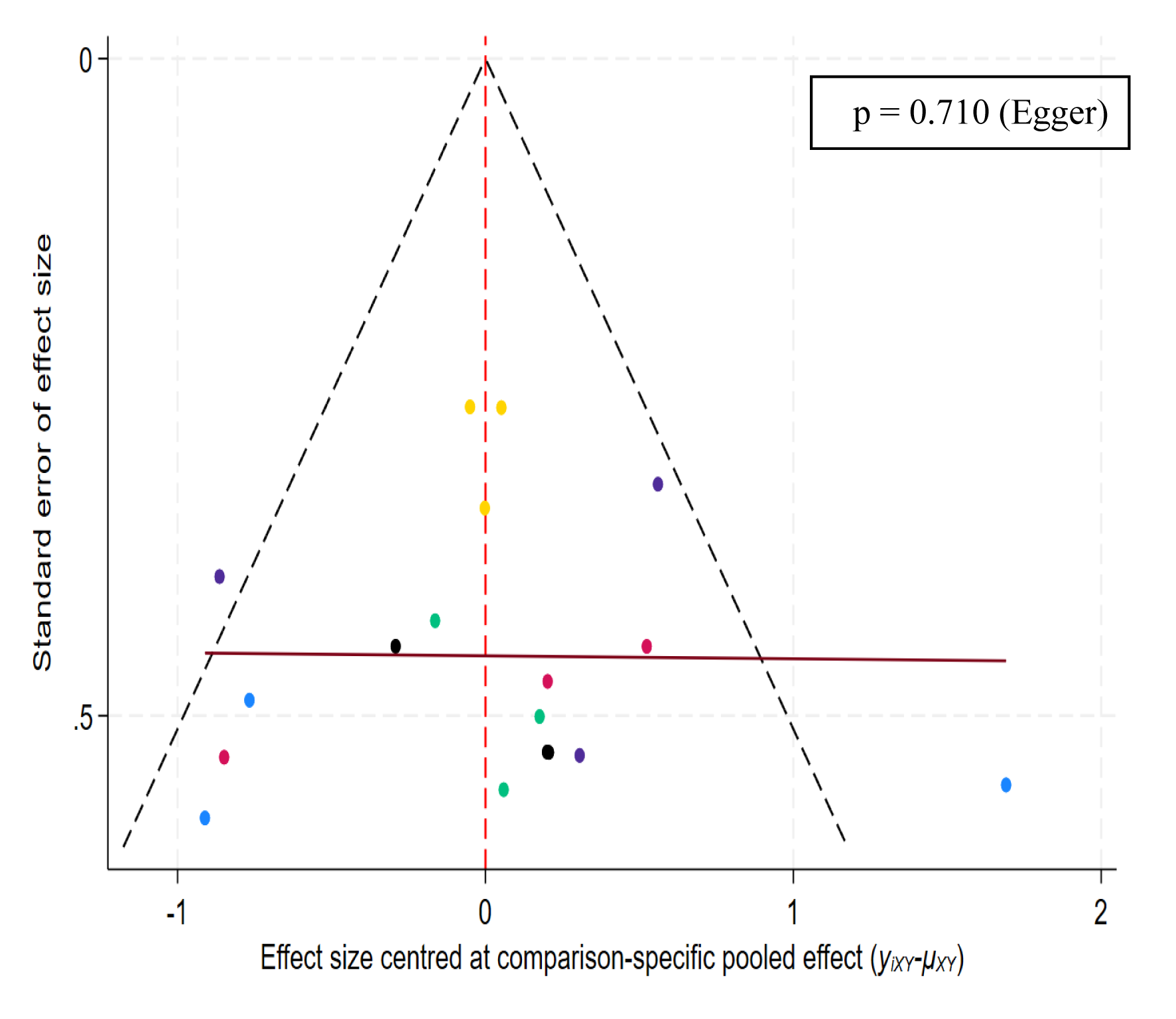

Supplement: Supplementary file 1 — Appendixes S1–S9: fsn371243‐sup‐0001‐AppendixesS1‐S9.docx. [file FSN3-13-e71243-s001.docx]
